# Cell contacts and pericellular matrix in the *Xenopus* gastrula chordamesoderm

**DOI:** 10.1371/journal.pone.0297420

**Published:** 2024-02-12

**Authors:** Olivia Luu, Debanjan Barua, Rudolf Winklbauer

**Affiliations:** Department of Cell and Systems Biology, University of Toronto, Toronto, Ontario, Canada; Laboratoire de Biologie du Développement de Villefranche-sur-Mer, FRANCE

## Abstract

Convergent extension of the chordamesoderm is the best-examined gastrulation movement in *Xenopus*. Here we study general features of cell-cell contacts in this tissue by combining depletion of adhesion factors C-cadherin, Syndecan-4, fibronectin, and hyaluronic acid, the analysis of respective contact width spectra and contact angles, and La^3+^ staining of the pericellular matrix. We provide evidence that like in other gastrula tissues, cell-cell adhesion in the chordamesoderm is largely mediated by different types of pericellular matrix. Specific glycocalyx structures previously identified in *Xenopus* gastrula tissues are absent in chordamesoderm but other contact types like 10–20 nm wide La^3+^ stained structures are present instead. Knockdown of any of the adhesion factors reduces the abundance of cell contacts but not the average relative adhesiveness of the remaining ones: a decrease of adhesiveness at low contact widths is compensated by an increase of contact widths and an increase of adhesiveness proportional to width. From the adhesiveness-width relationship, we derive a model of chordamesoderm cell adhesion that involves the interdigitation of distinct pericellular matrix units. Quantitative description of pericellular matrix deployment suggests that reduced contact abundance upon adhesion factor depletion is correlated with excessive accumulation of matrix material in non-adhesive gaps and the loss of some contact types.

## Introduction

The blastocoel wall of the *Xenopus* embryo consists of an outer epithelial sheet that coats layers of deep cells. For the epithelial layer, subapical adherens and tight junctions are characteristic [1–3] while deep cell adhesive contacts appear amorphous and are interspersed between non-adhesive interstitial gaps. We recently showed that such contacts can be characterized by their width spectra—the frequency distributions of membrane-membrane distances—and by the modifications of the spectra when adhesion factors are depleted [4, 5]. From the requirement for factors such as fibronectin (FN) or hyaluronic acid (HA), and from the large widths of contacts we concluded that gastrula cell-cell adhesion is largely mediated by the pericellular matrix (PCM) [4, 6]. In the *Xenopus* early embryo, a fraction of the PCM contains La^3+^ staining material (LSM) [7], which can be used to further characterize contact types. Thus, we found that some LSM patches resembled known endothelial glycocalyx variants which we termed glycocalyx I, II and III, and which mediated cell adhesion but occurred also on the free cell surfaces at interstitial gaps [4].

Previously, we analyzed ectoderm and prechordal mesoderm, which represent different germ layers and morphogenetic behaviors. Whereas ectoderm is stretched during epiboly, prechordal mesoderm performs active, directional cell-on-cell migration [8]. Despite the differences, contact types are largely shared between these tissues [4, 5]. Here, we analyze cell contacts in the chordamesoderm (CM), which is continuous with the ectoderm at its posterior and the prechordal mesoderm at its anterior end. It elongates by convergent extension, a cell intercalation process driven by protrusive activity at the ends of mediolaterally oriented bipolar cells, and by junction remodelling at antero-posterior cell-cell contacts [9–11].

Convergent extension depends on CM cell adhesion via the main cadherin of the *Xenopus* gastrula, C-cadherin (C-cad) [10, 12] and on the extracellular matrix protein FN [13], and we depleted these factors using morpholino antisense oligonucleotides. We also knocked down the small transmembrane/extracellular heparan sulfate proteoglycan Syndecan-4 (Syn-4) [4, 14], and we impeded the synthesis of the large glycosaminoglycan, HA, by knocking down HA synthases Has1 or Has2 [15]. Combined with the analysis of contact width spectra and La^3+^ staining, the knockdown experiments identified new contact types and showed that the CM contact pattern differs from both ectoderm and prechordal mesoderm. In all morphants the abundance of cell-cell contacts was reduced, but this was not paralleled by reduced average adhesiveness of the remaining contacts: a decrease of adhesiveness at low contact widths was compensated by the massive addition of wide contacts and an increase of adhesiveness proportional to width. From the latter observation we derive a model of CM cell-cell adhesion which is based on the interdigitation of PCM units from opposite membranes.

## Materials and methods

### Embryo manipulations

Adult *Xenopus laevis* were maintained in accordance with University of Toronto Animal Use Protocol (20011765). Eggs were fertilized in-vitro, de-jellied using 2% cysteine in 1/10 Modified Barth’s Solution (MBS; 88 nM NaCl, 1 mM KCl, 2.4 mM NaHCO_3_, 0.82 mM MgSO_4_, 0.33 mM Ca(NO_3_)_2_, 0.41 mM CaCl_2_, 10 mM Hepes (+NaOH), 1% streptomycin, 1% penicillin (pH 7.4) and kept in 1/10 MBS until stage 11. Morpholino antisense oligonucleotides (Gene Tools) were injected at the two-cell stage in 4% ficoll solution and embryos were incubated in 1/10 MBS at 15°C until stage 11. The following previously characterized morpholinos were used, at the efficiencies as percent reduction of protein levels indicated.

**Table:** 

**Morpholino**	**Sequence (5’–3’)**	**ng injected per blastomere**	**Efficiency (% reduction of protein)**
C-cadherin [16, 17]	CCACCGTCCCGAACAGAAGCCTCAT	20	65
Fibronectin (xFN1) [13, 18]	CGCTCTGGAGACTATAAAAGCCAAT	20	63
Fibronectin (xFN2) [13, 18]	CGCATTTTTCAAACGCTCTGAAGAC	20	63
xHas-1 [19, 20]	GTTGCCGAATGAAGAGGCCCCAAGA	27	*Unknown*
xHas-2 [19, 20]	TGCATATAAACCGTTCACAGTGCAT	30	*Unknown*
xSyn-4.1 [14, 21–23]	GCACAAACAGCAGGGTCGGACTCAT	20	75
xSyn-4.2 [14, 21–23]	CTAAAAGCAGCAGGAGGCGATTCAT	20	75

### Transmission electron microscopy (TEM)

For the TEM pictures, 4% paraformaldehyde and 2.5% glutaraldehyde in 0.05M cacodylate buffer at pH 7.0 were used to fix stage 11 gastrulae. Bisected samples were rinsed in 0.1M cacodylate and then fix in 0.1M cacodylate containing osmium tetroxide (1%). To visualize the glycocalyx, 1% lanthanum nitrate (Sigma-Aldrich Canada) was added to the fixatives. Samples were washed with 0.1M cacodylate and dehydrated in a series of graded ethanol solutions before embedding in Spurr’s resin. Semi-thin and ultrathin sections were obtained using a Leica EM UC6 microtome. Sections were stained with 3% uranyl acetate in methanol for 1 hour followed by 10 minutes in Reynold’s lead citrate. Images were taken with a Hitachi HT7700 microscope.

### Analysis of TEM images

Cell contacts were analysed as previously described [4]. In short, differences in cell and yolk platelet size were used to identify morphant gastrula tissues in TEM images from 3 embryos from different egg batches for each condition [4]. Stretches of cell perimeter were treated as contacts when the contours of adjacent cells followed each other. Their abrupt divergence indicated the end of a contact at a non-adhesive gap. Contact angles between cells were measured at the transitions between contacts and interstitial gaps. Contact width was measured as separation distance between membranes, distances were binned in 50 nm wide steps. LSM height in gaps was similarly measured. All contacts or angles in a TEM image were measured; for number of TEM images per treatment (see Figure legends). The error due to random tilting of the sectioning plane relative to the plane of a contact, as estimated from the width variation of tight junctions, can amount to an up to 1.5-fold apparent increase in contact width [4]. The respective distortion of contact angles broadens the angle distribution by a factor *k*, by making the narrowest angles appear even narrower and widening the widest angles, depending on the inclination of the sectioning plane [24, 25]. The factor *k* depends on the shape and size of the objects sectioned at random and the angle distribution. It is difficult to derive theoretically, given that the gaps in the tissues examined here are a heterogeneous mixture of differently sized bubbles and 3-to-multisided gaps. However, we found that when setting *k* ≥ 1.5 the corrected maximal and minimal boundary lines would intersect within the observed width range, whereas at a slightly lower *k* = 1.33 the lines would meet only beyond this range (S4 Fig). Variances between treatments were statistically analyzed using one-way ANOVA. Data visualization and statistical analyses were performed using Graphpad Prism 7 2017 v7.0.3. Vector graphics and figures were assembled using Inkscape v0.92 and v1.0.

## Results

### Abundance of cell contacts but not their adhesiveness is reduced in adhesion factor morphants

**Fig 1:**
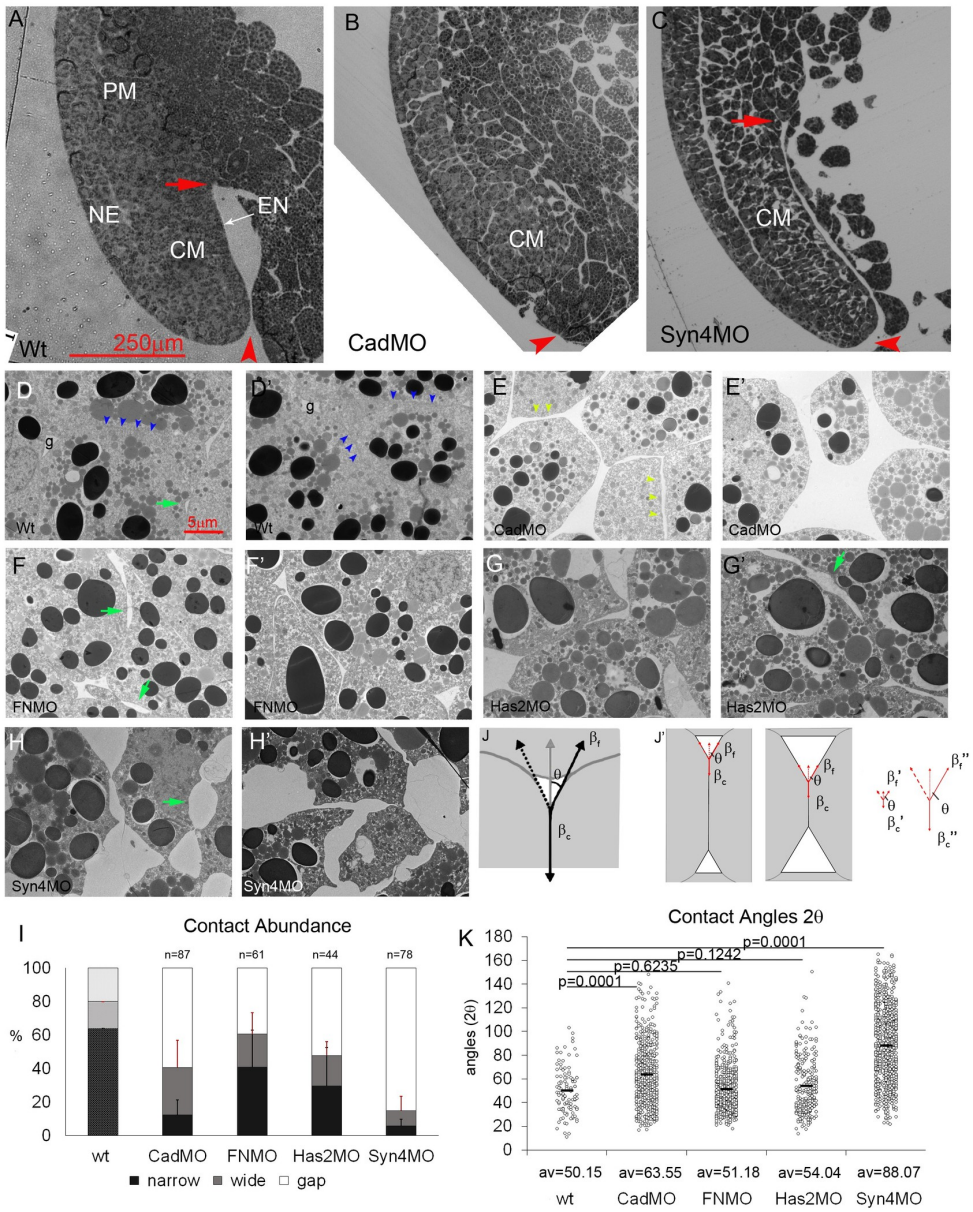
Abundance of cell contacts but not relative adhesiveness is reduced in adhesion factor morphants. (A–C) Dorsal side of normal (A) and C-cad depleted stage 11 gastrulae (B). In Syn-4 depleted embryos (C) convergent extension appears slightly accelerated in 6 out of 7 gastrulae. CM, chordamesoderm; NE, neural ectoderm; PCM, prechordal mesoderm; EN, suprablastoporal endodermal epithelium; red arrow, tip of archenteron; red arrowhead, position of blastopore. (D–H’) Cell packing in normal (wt) and morphant (MO) stage 11 chordamesoderm. Blue arrowheads, narrow contacts between cells; green arrows, two-sided gaps (“bubbles”) between two cells; light green arrowheads, wide contacts between cells; g, gaps at 3- or 4-cell junctions. (I) Abundances of narrow (< 50 nm) and wide (> 50 nm) contacts, and interstitial gaps. Bars, standard deviations. (wt) is from Barua et al. [4]. n, number of TEM images analyzed. (J) Tensions at 3-sided gap. For tension *β*_*f*_ at free gap surface to balance tension per cell *β*_*c*_ at contact interface it must act at a contact angle *θ*. (J’) Same contact angle *θ* and thus relative adhesiveness *α* can combine with large (left) or small (middle) contacts (small or large gaps, respectively). Same *θ* can be generated by smaller ($\beta_{f}^{\prime }$ and $\beta_{c}^{\prime }$) or larger ($\beta_{f}^{\prime \prime }$ and $\beta_{c}^{\prime \prime }$) tensions, provided that their ratio is retained. (K) Each dot represents a measured angle 2*θ* in normal and morphant CM; av., average.

At the stage 11 middle gastrula, the blastopore has invaginated to form the archenteron, and the CM has involuted at the blastopore lip to elongate in the animal-vegetal direction between the endodermal archenteron lining and the neural ectoderm (Fig 1A). Depletion of any of four select adhesion factors—C-cad, FN, HA and Syn-4—by morpholino antisense oligonucleotides affects the CM [4]. For example, knockdown of C-cad arrests CM involution, elongation, and archenteron formation (Fig 1B), like that of FN [4], and the depletion of Syn-4 severely alters cell packing and shape but surprisingly, preserves CM movements including convergent extension (Fig 1C).

CM cells are tightly packed, with occasional interstitial gaps at 3-cell junctions and interstitial “bubbles” between two cells (Fig 1D and 1D’). C-cad knockdown increases the number and size of interstitial gaps, and evenly spaced wide cell-cell contacts become prominent (Fig 1E and 1E’). FN depletion increases interstitial gaps moderately and widens cell-cell contacts or separates cells (Fig 1F and 1F’). Knockdown of Has-2 also increases gaps and contact widths (Fig 1G and 1G’), suggesting an adhesive role of HA. Contact loss is most severe in Syn-4 morphants, where cells remain connected only through thin processes. The cell surface is often concave, generating extensive interstitial space and a serrate cell outline (Fig 1H and 1H’). Overall, all four factors normally contribute to the dense packing of the CM cells.

To quantify the abundance of adhesive cell contacts (Fig 1I), we measured the lengths of stretches where the membranes of adjacent cells ran parallel until they diverged abruptly at interstitial gaps [4]. We distinguished between narrow contacts with membrane distances < 50 nm, and the remaining, highly variable wide contacts. Non-attached free surfaces are present at interstitial gaps [4]. In CM cells, 4/5 of the surface is normally engaged in adhesive contacts. Narrow contacts occupy almost 2/3 of the cell surface, and 80–90% of these depend on both Syn-4 and C-cad. Unexpectedly, half of the narrow contacts also require the large HA and FN molecules. As in ectoderm or prechordal mesoderm [4], Syn-4 depletion has the strongest effects, suggesting that virtually all narrow contacts and 1/3 of wide contacts require the presence of Syn-4 (Fig 1I). All treatments increase the abundance of non-adhesive gaps.

The abundances of contact widths were collected in 50 nm bins to generate width spectra; changes due to experimental interference are best seen in difference spectra where values of untreated samples are subtracted from experimental ones [4]. In CM, width abundances decrease sharply after a maximum at < 50 nm and vanish beyond 700 nm [4] (S1 Fig). C-cad, FN, Has-2 and Syn-4 depletion strongly reduce abundances of narrower contacts, increase the frequency of wider ones, and generate contacts beyond the normal width range (S1 Fig). Notably, Has-2 knockdown does not produce the signature glycocalyx I difference spectrum—a distinct decrease at 50–100 nm and increase at < 50 nm that is induced by this treatment in prechordal mesoderm or ectoderm [4]. In fact, compared to the ectoderm [4], the 50–100 nm width abundance is lowered in the CM spectrum, reproducing the glycocalyx I depletion signature in the CM-ectoderm difference spectrum (S1 Fig) and suggesting a lack of this structure in the CM.

The adhesive strength of contacts depends on the difference in surface energy per area between free and contacting cell surfaces at gaps, *β*_*f*_ − *β*_*c*_, i.e., on how much tension at contacts is reduced relative to the free surface, for example by the release of adhesion factor binding energy. The ratio of the tensions is *β*_*c*_/*β*_*f*_ = cos *θ* with *θ* the contact angle between adjacent cells. The less the tension is reduced at contacts, the smaller would be the adhesion strength at these contacts and hence *θ* (Fig 1J) [16, 26]. On the other hand, smaller or larger contact area and correspondingly increased or decreased gap size is compatible with the same *θ*, the same tension magnitudes, and hence the same adhesion strength in contacts (Fig 1J’). However, all tensions being altered proportionally would change adhesion strength, also without affecting *θ* (Fig 1J’). In the CM, angles remain the same upon FN- and Has-2 knockdown and are increased by C-cad and Syn-4 depletion (Fig 1K). Thus, while adhesive contact abundances are diminished in morphants, the relative reduction of tension at the residual contacts—their relative adhesiveness—is not decreased. This indicates that the abundance and the adhesiveness of contacts can be decoupled experimentally, and both parameters must be determined independently. If absolute adhesion strength, measured as surface energy per area, were changed in the morphants while relative adhesiveness and hence contact angles were retained, this would still be compatible with large or small gaps (Fig 1J’). The case that both relative adhesiveness—the ratio of tensions—and absolute adhesion strength—the difference between tensions—are altered at the same time is analyzed in detail in the Discussion section. Generally, however, diminished cell packing density is not necessarily linked to reduced adhesion strength.

### La^3+^ staining and adhesion factor depletion identify contact types

**Fig 2:**
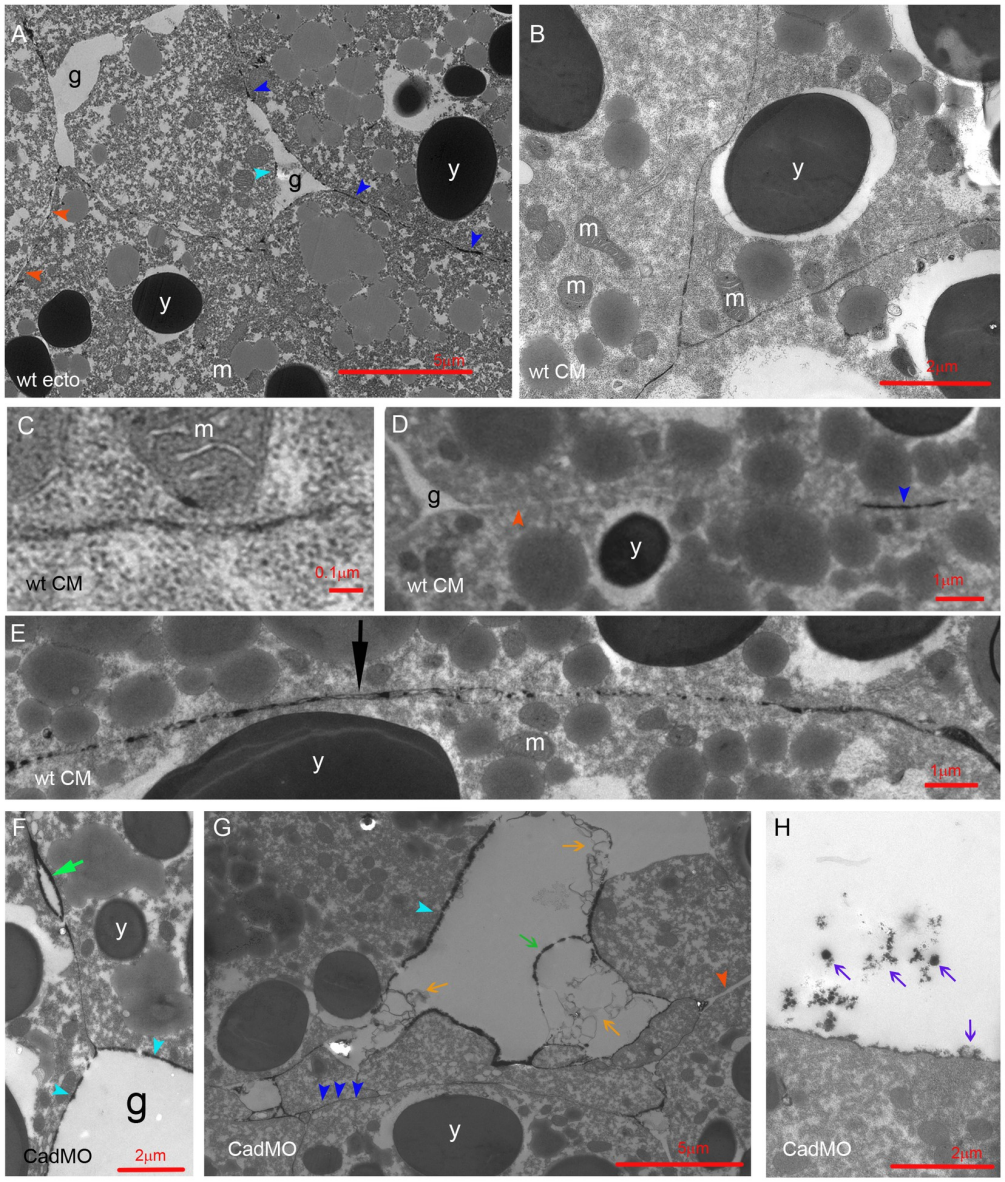
LSM in normal and C-cad depleted CM. (A) LSM in ectoderm, for comparison. (B–E) LSM in normal CM. (F–H) C-cad morphant CM. g, interstitial gaps; y, yolk platelets; m, mitochondria; dark blue arrowheads, LSM plaques in contacts; light blue arrowheads, plaques on gap surfaces; red arrowheads, LSM-free contacts; black arrow, triple-layered contact; light green arrow, bubble; dark green arrow, shed plaque; orange arrows, lightly stained shed ribbons; purple arrows, darkly stained shed LSM.

La^3+^ stains sections of the adhesive contacts, and together with the knockdown of adhesion factors, this can be used to identify contact types. In the ectoderm, isolated LSM plaques occur in contacts and on the surface of interstitial gaps (Fig 2A) [4]. Cells in the more densely packed CM are often outlined by delicate lines of La^3+^ staining, which are occasionally interrupted, meet at gap-less 3-cell junctions (Fig 2B), can be as narrow as 10 nm (Fig 2C). Some contacts and rare gaps at 3-cell junctions are unlabeled (Fig 2D). In triple-layered contacts, La^3+^ resolves into two parallel lines with or without small LSM dots between (Fig 2E). No bush-like glycocalyx II or brush-like glycocalyx III structures [4] were observed.

**Fig 3:**
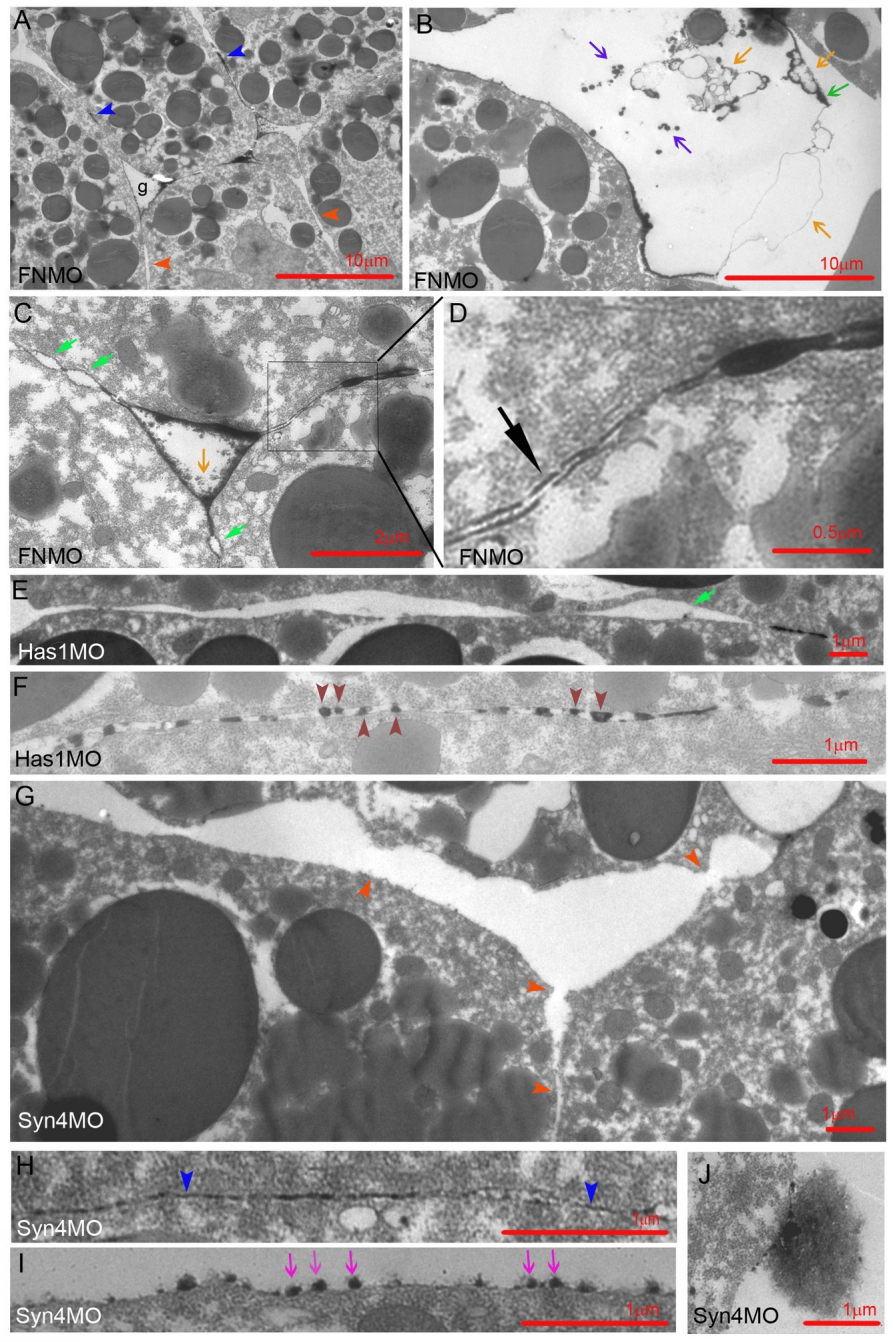
LSM in FN, Has1 and Syn-4 depleted CM. Dark red arrowheads, semi-drop-like LSM sitting on upper or lower membrane; dark blue arrowheads, LSM plaques in contacts; light blue arrowheads, plaques on gap surfaces; red arrowheads, LSM-free contacts; black arrow, triple-layered contact; light green arrow, bubble; dark green arrow, shed plaque; orange arrows, lightly stained shed ribbons; purple arrows, darkly stained shed LSM; magenta arrows, LSM dots.

Upon C-cad depletion, LSM becomes concentrated in plaques on the surfaces of interstitial gaps (Fig 2F). FN morphants show a similar pattern (Fig 3A and 3C), and moreover, parallel lines of strong staining can enclose a less stained central layer in contacts (Fig 3C and 3D). Depletion of HA generates long unlabeled contacts (Fig 3E) or breaks LSM lines up into short plaques or rows of droplets that sit with a broad base on one cell surface and touch with their apex the opposite cell (Fig 3F). In Syn-4 morphants, cell surfaces are mostly devoid of La^3+^ staining (Fig 3G), but 10–20 nm LSM contacts are preserved (Fig 3H), and some surfaces are coated with small, sparse LSM dots (Fig 3I). Three types of LSM shedding occur. In gaps of C-cad or FN morphants, complete plaques and long, faintly stained ribbons detach (Figs 2G and 3B); groups of small, dense LSM flakes are also shed (Figs 2H and 3B); and in Syn-4 morphants, hairballs of fine fibrils (Fig 3J) consist probably of HA [4]. Thus, as in ectoderm or prechordal mesoderm, Syn-4 is the main contributor to LSM formation. Syn-4 and HA seem to generate extended LSM plaques, which are prone to shedding though in the absence of C-cad or FN.

**Fig 4:**
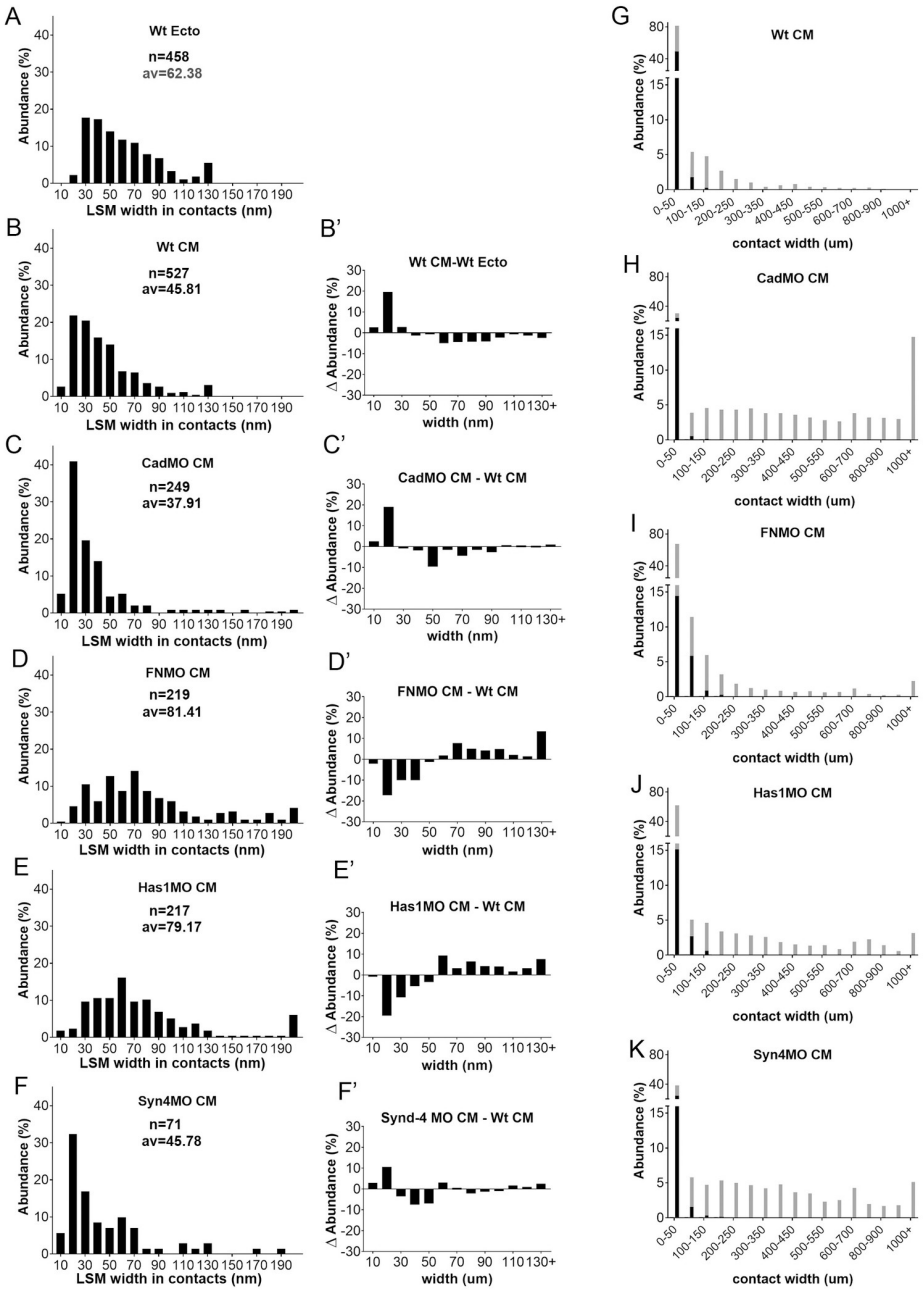
LSM widths in contacts. (A–F) Width frequency distributions in ectoderm (A) for comparison, in CM (B) and in various CM morphants (C–F). n, number of width measurements from 18, 4, 6, 8, 3 TEM images, respectively; av., average. (B’–F’) Corresponding difference (ΔAbundance) spectra comparing width distributions of CM to ectoderm (B’), and of morphants to normal CM (C’–F’). (G–K) Comparison of widths of LSM-containing (black parts of bars) and LSM-free (grey parts of bars) contacts, using the data from (B–F) and S1 Fig.

LSM-filled contacts were further characterized by their width spectra. In ectoderm, width abundances decrease from a maximum at 20–30 nm (Fig 4A). In the CM (Fig 4B and 4B’), widths decrease first gradually from a maximum at 10–20 nm, then abruptly at 60 nm, and abundances remain low in the 60–120 nm range which in ectoderm harbors glycocalyx I, consistent with this glycocalyx type being diminished in the CM. A new CM peak at 10–20 nm is apparent in the CM-ectoderm difference spectrum (Fig 4B’), and C-cad-MO (Fig 4C and 4C’) and Syn-4-MO (see Fig 4F and 4F’) spectra confirm the subdivision of narrow LSM contacts into C-cad/Syn-4-independent 10–20 nm and -dependent 20–50 nm contacts. In FN and Has1 morphants, all LSM contacts < 50 nm are strongly diminished, and 50–100 nm contacts increased. HA and FN are particularly required in 10–20 nm contacts (Fig 4D–4E’). In C-cad and Syn-4 morphants, triple-layered contacts (see Fig 2E) are absent, and in Has-1 and FN morphants, their peak is shifted from 30 to 70 nm (S2 Fig). Thus, C-cad and Syn-4 are essential for these contacts while HA and FN control their width.

A large part of the PCM is not stained by La^3+^ and respective contacts are less well defined (Fig 4G–4K). Almost half of narrow < 50 nm contacts are unlabeled in the CM. They are essentially removed by C-cad and Syn-4 knockdown while wider than normal contacts appear, consistent with a widening of the original contacts. In FN and Has1 morphants, unlabeled contacts are little affected. Thus, narrow C-cad- and Syn-4-dependent unlabeled PCM contacts form part of the CM contact complement. Of note, LSM is restricted to widths below 200 nm, leaving the wider contacts, predominant in morphants, unlabeled.

### PCM distribution in contacts and in non-adhesive gaps

**Fig 5:**
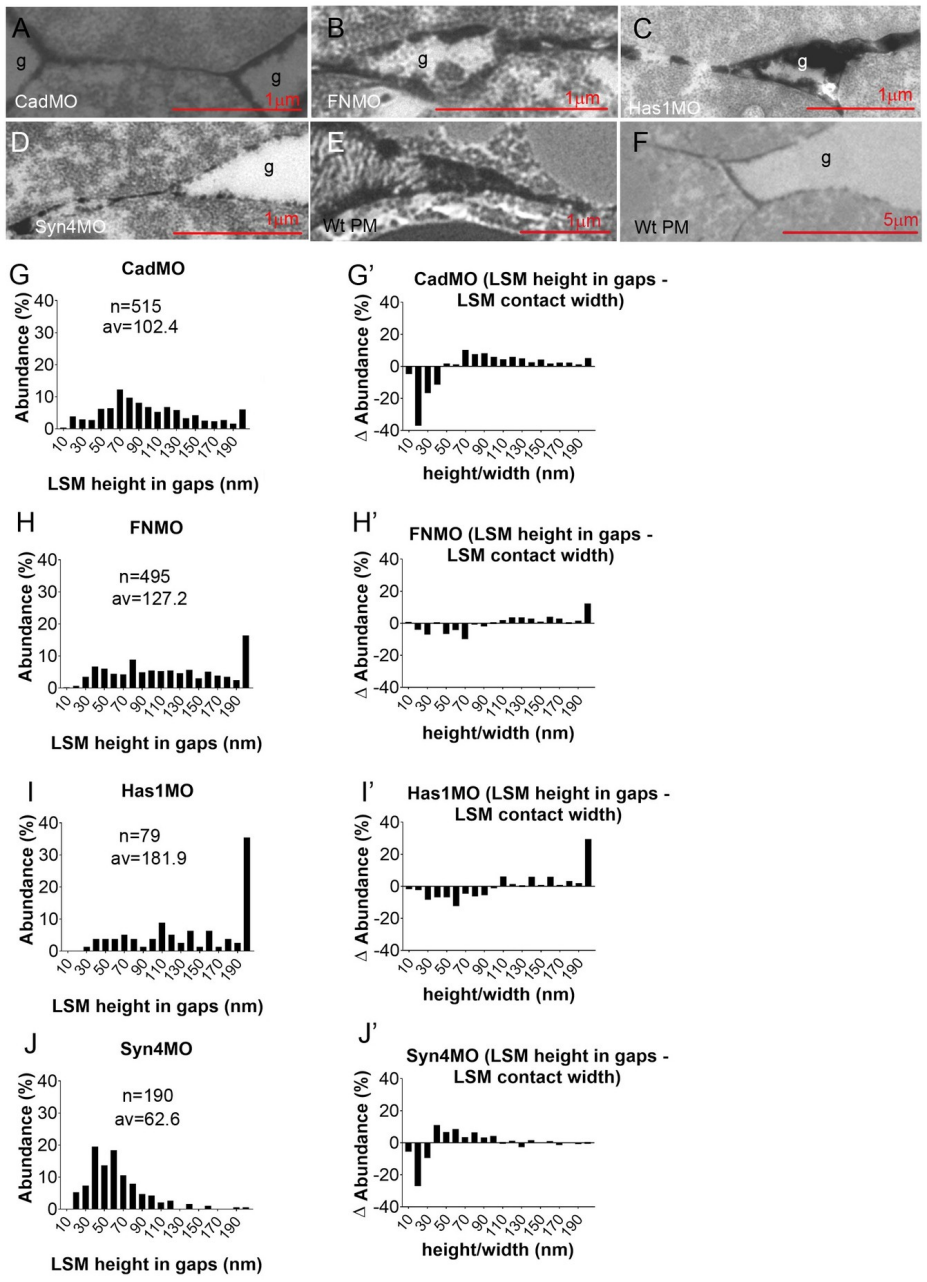
LSM in gaps. (A–F) Examples of LSM at transitions from gaps (g) to cell-cell contacts. PM, prechordal mesoderm. (G–J) Frequency distributions of LSM height in gaps (corresponding to LSM width in contacts). n, number of measurements from 5, 6, 3, 7 TEM images, respectively; av., average. (G’–J’) Difference (ΔAbundance) spectra corresponding to (G–J), comparing LSM height in gaps to LSM width in contacts.

LSM is typically more prominent in non-adhesive gaps than in contacts (Fig 5A–5F). Thick layers of LSM can accumulate symmetrically on neighboring sides of a gap in C-cad, FN and Has morphants while the width of an adjoining LSM contact is much thinner than the height of the gap surface LSM (Fig 5A–5D). Examples from the more frequent gaps in prechordal mesoderm show that this is also true for normal tissue (Fig 5E and 5F). Notably, when the LSM forms distinguishable units, these seem to interdigitate in contacts (e.g. Fig 5B and 5E; see also [4]) to generate contacts only as wide as the units are high in each single layer. Compared quantitatively to LSM contact widths in normal CM (see Fig 4), even single LSM layers are higher in gaps in the morphants (Fig 5G–5J), and the respective frequency distributions indicate 2.2-fold, 2.8-fold, 4.0-fold and 1.4-fold increases in gaps of C-cad, FN, Has1 and Syn-4 morphants, respectively. Gap-contact difference spectra confirm reduced abundances of narrow LSM layers and an increase of thicker layers, consistent with the width of contacts not being derived from adding up the heights of LSM layers in gaps.

**Fig 6:**
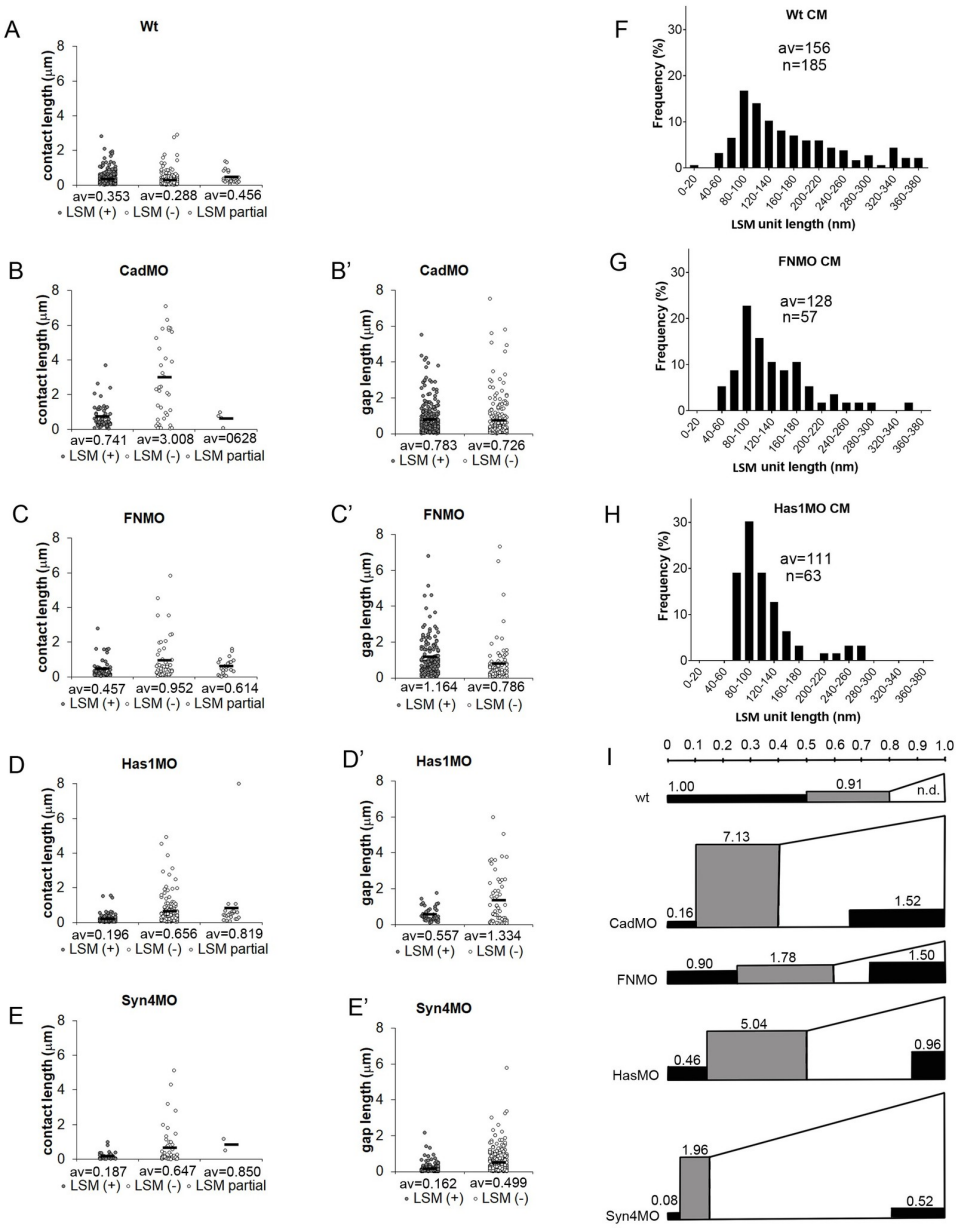
Lengths of PCM stretches. (A–E) Lengths of continuous stretches of LSM-containing (LSM(+)) contacts (plaques), and of LSM-free (LSM(-)) and triple-layered (LSM partial) contacts. (B’–E’) Lengths of continuous stretches of LSM (plaques) (LSM(+)) on gap surfaces or LSM-free surfaces (LSM(-)). Data from 4, 5, 9, 8, 6 TEM images, respectively; av., averages. (F–H) Lengths of shortest LSM units discernible in normal CM contacts (F), and in contacts and gaps of morphants (G,H). n, number of unit LSM structures measured, from 4, 7, 5 TEM images, respectively. (I) Summary diagram integrating LSM (black areas) height and length data for contacts (left, rectangular) and gaps (right, wedged), and non-labeled contact height and length data for contacts (grey, rectangular). Area size relative to LSM in normal CM contacts (1.00) is indicated. Scale on top, relative average lengths of contact types. Height of rectangles, relative average widths of respective structures. Areas are proportional to respective PCM volumes.

In CM contacts, the lengths of continuous LSM, non-stained, or triple-layered stretches are similar (Fig 6A), and similar to those of LSM plaques in prechordal mesoderm [4]. In morphants, LSM stretches are lengthened or shortened moderately while the lengths of non-stained stretches are always increased, in C-cad-MO embryos by 10-fold (Fig 6B–6E). Random removal of some LSM plaques interspersed in non-labeled contact stretches would cause this effect. LSM plaque length in gaps is comparable to that in contacts in C-cad and Syn-4 morphants and increased in FN and Has1 morphants (Fig 6B’–6E’). The lengths of unlabeled stretches in gaps (Fig 6B’–6E’) suggest also random removal or addition of LSM stretches in non-labeled regions.

To see whether large plaques could be composed of smaller units, we measured the lengths of the smallest discrete LSM structures in normal CM contacts (e.g. Fig 2E), in contacts and gaps of FN morphants (e.g. Fig 5B), and the LSM droplets in HA depleted CM (e.g. Fig 3F). We consistently found a peak at 100 nm with a slow decrease towards higher values (Fig 6F–6H). In the plaque size distribution of CM (Fig 6A), these unit structures would occupy the lower margin. This is consistent with larger plaques being assembled from small units, and combinations of different LSM and non-LSM units generating patterns of alternating contact types.

The effects of adhesion factor depletion on PCM distribution are summarized in Fig 6I. In CM, more than half of adhesive contact length is La^3+^-stained. In morphants, overall contact length is reduced and LSM contacts shrink disproportionally, from half of the total cell surface in normal CM to 1/4^th^ or 1/5^th^ in FN, C-cad and Has2 morphants, and less than 1/10^th^ upon Syn-4 depletion. Non-labeled contacts remain at normal lengths except in Syn-4 morphants. In randomly sliced samples, volumes of object are proportional to their averaged cross-section areas, and for the volume of LSM or unlabeled PCM in contacts, relative contact length was multiplied by relative width (Fig 6I). C-cad or Syn-4 depletion reduces the contact volume of LSM by an order of magnitude. HA and FN depletion combine shortening of LSM contacts with their widening. Non-labeled contacts, although shorter, are wider in normal CM and their volume equals that of the LSM contacts (Fig 6I). In C-cad morphants, width is dramatically increased, and the volume of non-labeled contact augmented by almost 8-fold. Width increases lead also to 2–5.5-fold higher contact volumes in the other morphants (Fig 6I). The volumes of LSM and non-labeled contacts combined increase between 1.4- and 3.8-fold in the morphants, except for Syn-4 knockdown where the total is unchanged.

In gaps (Fig 6I), more than half of the cell surface is coated with LSM in FN and C-cad morphants, and almost a quarter in HA or Syn-4 depleted CM. LSM layer thickness is increased compared to contacts, and together with the increase in overall gap size in all morphants, this amounts to a large LSM volume in gaps which dominates the total volume of LSM. In FN morphants, total LSM volume exceeds that of untreated CM by 2.5-fold, with the excess being accumulated in the gaps. In C-cad and Has1 morphants, a reduced LSM volume in contacts combines with increased LSM in gaps, as if material were redistributed with only a modest increase in total volume. In Syn-4 morphants total LSM volume is reduced by half, suggesting that Syn-4 promotes LSM deposition or is itself a main part of the LSM [4]. The amount of non-labeled PCM in gaps remains unknown, but even if it were completely absent, adhesion factor depletion causes a considerable increase of total PCM volume. This non-intuitive effect of adhesion factor loss could be due to the disregulated, excessive production of PCM material, or to the swelling of existing PCM for example in response to reduced PCM cross-linking, or to both.

### Relative adhesiveness is a function of contact width

**Fig 7:**
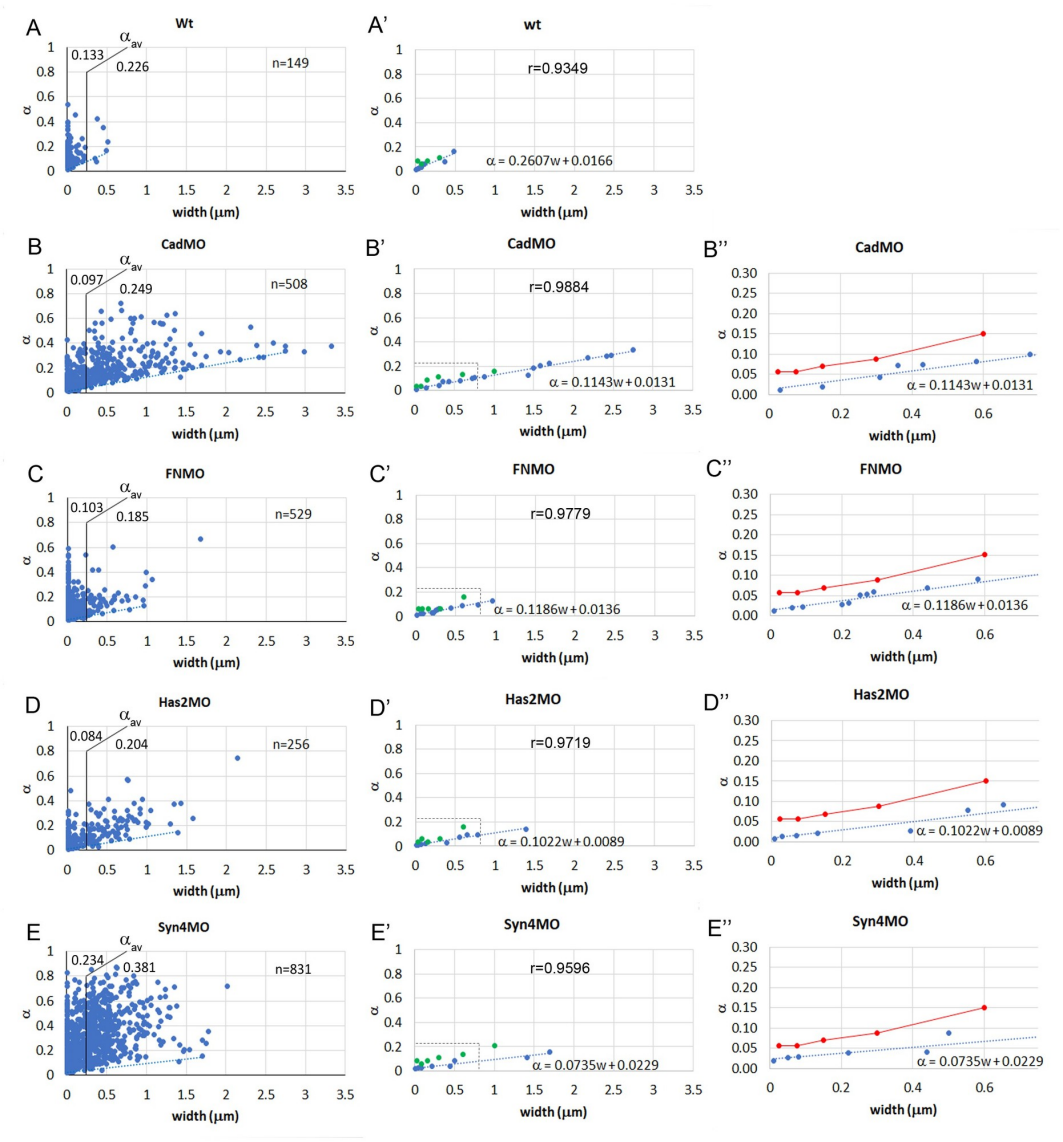
Relationship between relative adhesiveness and contact width. (A–E) Relative adhesiveness *α* was determined from contact angles and plotted as a function of contact width *w* for each gap-contact transition. The average value of *α*, *α*_*av*_, is indicated for *w* smaller or larger than 250 nm (see S1 Table). n, number of transitions measured in 26, 86, 59, 41, 80 TEM images, respectively. (A’–E’) A linear regression line (dotted blue line) was fit to the lowest values in each plot (blue dots). The slope Δ*α*/Δ*w* and the *α*-axis intercept *α*_0_ of the regression line *α* = (Δ*α*/Δ*w*)*w* + *α*_0_ are indicated. r, correlation coefficient for regression line. Green dots, values for *α* frequency peaks (see Fig 8 and S3 Table). (B’’–E’’) Higher magnification of (B’–E’) focusing on small *w*. *α* frequency peak data in (B’–E’) were pooled (red dots and lines) (see S3 Table) and compared to lower-boundary regression lines (blue).

Contact angles and contact width are both spread over a wide range in normal and morphant CM, and we asked whether the two parameters are correlated. Importantly, the contact angle *θ* between cells is related to the dimensionless relative adhesion strength *α*, i.e. at gaps to the adhesion strength *σ*_*i*_ = *β*_*f*_ − *β*_*c*_ normalized to the tension at free surfaces, *α* = *σ*_*i*_/*β*_*f*_ = 1 − *β*_*c*_/*β*_*f*_ = 1 − cos *θ* (Fig 1J; see also Fig 9) with 0 (no adhesion) ≤*α* ≤ 1 (maximal adhesion) [16, 26]. Overall, *α* increases with width *w* in normal and in morphant CM (Fig 7A–7E). In respective scatter plots, *α* values are concentrated above a lower boundary but become more dispersed farther above and with increasing *w*. In normal CM, most *α* values reside at *w* < 250 nm, and their average in this range is significantly lower in C-cad, FN, and Has morphants compared to normal CM (S1 Table), as perhaps expected from a removal of adhesion factors. However, for *w* > 250 nm, *α* is strongly increased, and the expanded width ranges in the morphants allow to compensate their reduced adhesiveness at *w* < 250 nm such that overall relative adhesion strength remains at a normal, high level. Syn-4 morphants differ, showing increased average *α* at both width ranges.

The *α*-*w* relationship is conveniently analyzed for the lower boundary of *α* values. We constructed it by selecting the data points on the enveloping curve, proceeding from one such point to the next point which is higher but does not require moving lower again subsequently. This was repeated up to where points became sparse (Fig 7A’–7E’). A linear regression line was then fitted through the enveloping points as *α* = (Δ*α*/Δ*w*)*w* + *α*_0_ with Δ*α*/Δ*w* the slope of the line and *α*_0_ its extrapolated value at *w* = 0 (Fig 7A–7E’). These parameters were similarly decreased in C-cad, FN and Has morphants compared to normal CM. Syn-4 morphants had increased values (see S2 Table).

**Fig 8:**
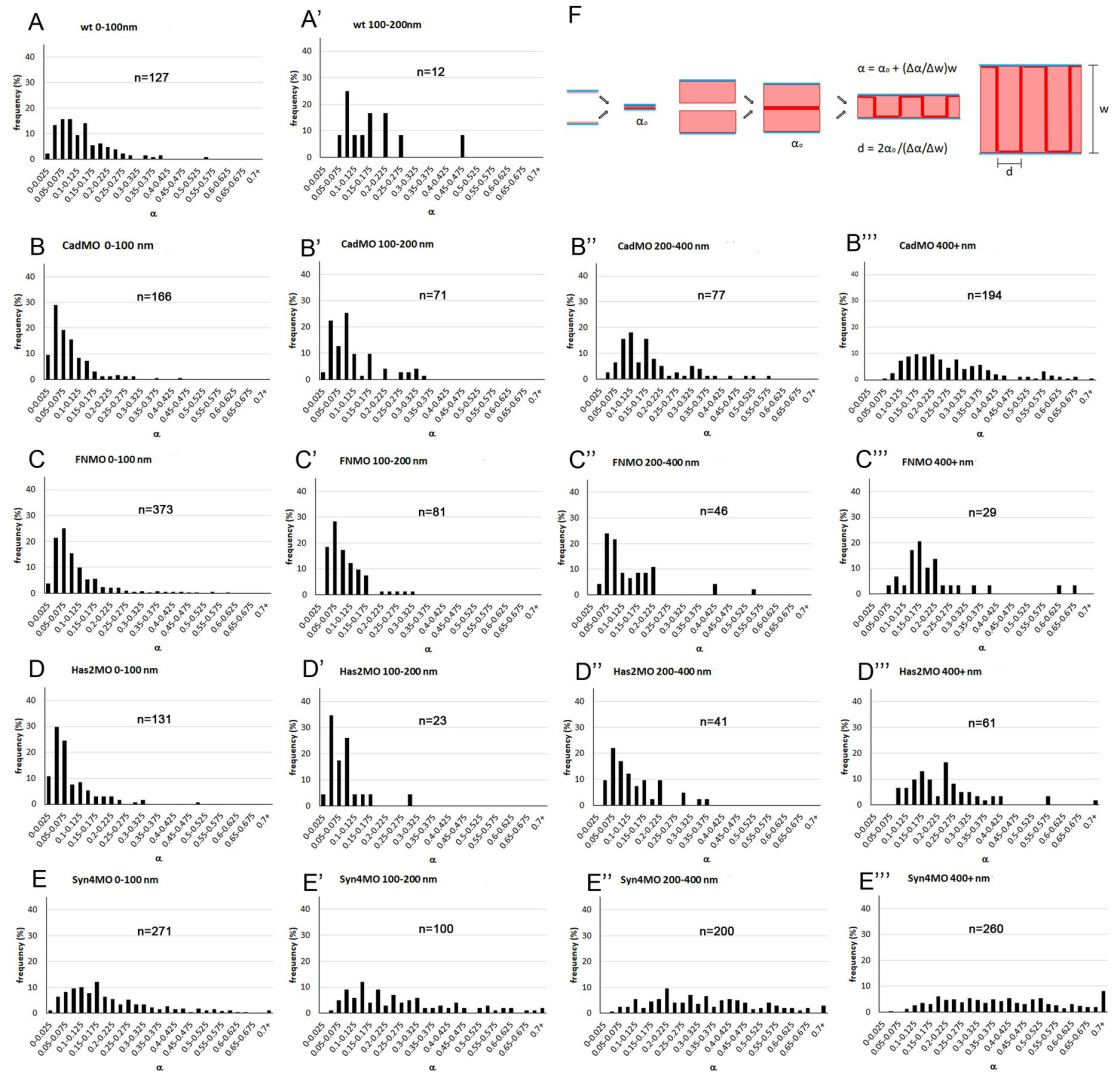
Frequency distributions of *α* values at different widths. (A-E’’’) Different treatments are arranged vertically and width brackets horizontally, as indicated on top of each diagram. n, number of *α-w* data points. Additional width brackets are shown in S3 Fig (F) Model of cell-cell adhesion by PCM interdigitation. Adhesion between two thin or thick PCM layers (light red) on cell membranes (blue) occurs in principle through a narrow interaction zone (deep red), yielding in each case the basic adhesiveness *α*_0_ (left). Interdigitation of the two apposed PCMs corresponds to a folding of the interaction surface which increases linearly with PCM height *w* at constant interdigitation distance *d* (right).

To examine the *α*-*w* relationship above the lower boundary, we determined the frequency distributions of *α* values for consecutive width brackets. Overall, the distributions are skewed with a first peak near the lower boundary and a long tail tapering off irregularly at higher values (Fig 8A–8E and S3 Fig). Except for Syn-4 morphants, this first peak is usually the main peak of the distribution. It is not shifting noticeably to higher values over the first two width brackets, centered around 25 and 75 nm (S3 Fig), which were thus combined in Fig 8A–8E. As distributions shift to higher *α* values with widths above 200 nm, minor peaks or shoulders appear or become more prominent, and differences between treatments more obvious (Fig 8A–8E). Particularly, in Syn-4 morphants, frequencies are spread out almost evenly over a large *α* range (Fig 8E).

The frequency of *α* values declines rapidly with increasing *w* (Fig 7A–7E), and with the heavily skewed *α* frequency distributions (Fig 8), large *α* values disappear preferentially at higher *w*, artificially lowering the nominal average of *α*. Instead, the first peak of the *α* frequency distributions can be followed to analyze the *α*-*w* relationship above the lower boundary (Fig 7A’–7E’’). Only few points, at low resolution, are obtained (Fig 7A’–7E’), but the values for all morphants are similar (S3 Table) and are thus pooled. The line connecting the averages increases linearly for *w* > 100 nm, but at a steeper slope than the lower-boundary line. For smaller widths, it seems to remain constant, in contrast to the lower boundary (Fig 7B’’–7E’’).

Contact-gap transitions are sectioned at randomly oriented planes and distortion of contact angles broadens their distribution, letting narrow angles appear even narrower and wide angles even wider [25]. The variation of *α* values is broadened accordingly by the factor *k*, and we estimated *k* ≈ 4/3 (see Materials and methods). Correction by this factor tilts lower boundary and peak lines upward (S4 Fig). The effect should be the same for all treatment conditions, and the span of the different *α* frequency distributions is also of the same order of magnitude. It is the range of contact widths *w* that differs several-fold between treatments, and together with *α* ∼ *w*, this determines the overall relative adhesiveness of normal and morphant CM.

## Discussion

### Cell-cell contact types in the chordamesoderm

In terms of cell packing density, in vitro cell motility, and contact spectra, the CM resembles the ectoderm more than the prechordal mesoderm [4, 27]. However, ectoderm and prechordal mesoderm share contact types while the CM, located between and linking these two regions, is different. In prechordal mesoderm and ectoderm, a glycocalyx I is identified via its difference spectrum characteristics, and glycocalyx II and III are recognized by their morphology [4]. None of these structures are seen in the CM. Instead, a 30 nm wide, triple-layered contact type with a non-labeled layer between two LSM sheets is present in the CM. Another, 10–20 nm wide LSM contact in the CM is narrower than adherens junctions and insensitive to C-cad depletion but depends on the large HA and FN molecules. Despite their size, these factors can reside in narrow spaces. CNS synapses 20 nm wide harbor HA and its CD44 receptor [28, 29], and the string-like FN protein likewise occurs in synaptic clefts [30]. HA chains thousands of nm long occupy large volumes when randomly coiled, but when attached to a surface can form 0.3 nm thin layers [31]. Thus, HA and FN could directly build the 10–20 nm contacts. Non-LSM contacts are prominent in the gastrula but due to the lack of respective staining are less well characterized. In the CM, some narrow < 50 nm non-LSM contacts depend on C-cad, consistent with the presence of adherens junctions [1].

### Pleiotropic and polygenic control of contacts by adhesion factors

The CM results confirm our previous findings that adhesion factors are pleiotropic, each affecting various contact types, while contact types in turn are each controlled by several adhesion factors [4, 5]. As an example for pleiotropic functions, HA acts together with Syn-4 and other factors in ectoderm and prechordal mesoderm to build a 50–130 nm wide glycocalyx I, and in prechordal mesoderm a micron-wide brush-like glycocalyx III [4]. In the CM, HA supports instead extremely narrow 10–20 nm LSM contacts. On the other hand, glycocalyx I is the prime example for a structure that depends on multiple adhesion factors, in the prechordal mesoderm at least on HA, Syn-4, Glypican-4, PAPC, ephrinB3 and EphB4 [4, 5]. The CM provides additional examples. Among only 4 factors tested, the 10–20 nm contacts require HA and FN, some 30–50 nm contacts C-cad and Syn-4, and triple-layered contacts all four factors.

The picture emerges that different adhesion factors, in variable combinations, build a mosaic of adhesive contact complexes whose molecular and mechanical details are yet to be studied. Depletion of a factor often modifies a contact. For example, upon depletion of HA, 10–20 nm LSM contacts disappear and wider, beaded LSM contacts appear, suggesting a change in width and structure, but not in adhesive role. Likewise, triple layered LSM contacts become wider in FN morphants, and non-LSM contacts apparently in all morphants. Modification, not dismantling was also observed in endothelial glycocalyx when HA or heparan sulfate were enzymatically removed [32]. Modification of a contact type could include becoming non-adhesive. Thus, the apparent redistribution of LSM from contacts to gaps could be due to the accumulation in gaps of LSM rendered non-adhesive. The overall reduction of LSM upon Syn-4 depletion and the absence of triple layered contacts in C-cad and Syn-4 morphants suggest that contact types can also be lost. Quantitatively similar *α*-*w* curves in different morphants and across contact widths ranges suggest similar adhesiveness of the different residual adhesion types. Predominantly non-specific adhesion between PCMs by van der Waals forces, hydrogen bonds, Ca^2+^ bridges, or chain entanglement [33–37] would contribute to this, while specific interactions of adhesion factors could play mostly structural roles in the PCM.

### Adhesion strength in the chordamesoderm

**Fig 9:**
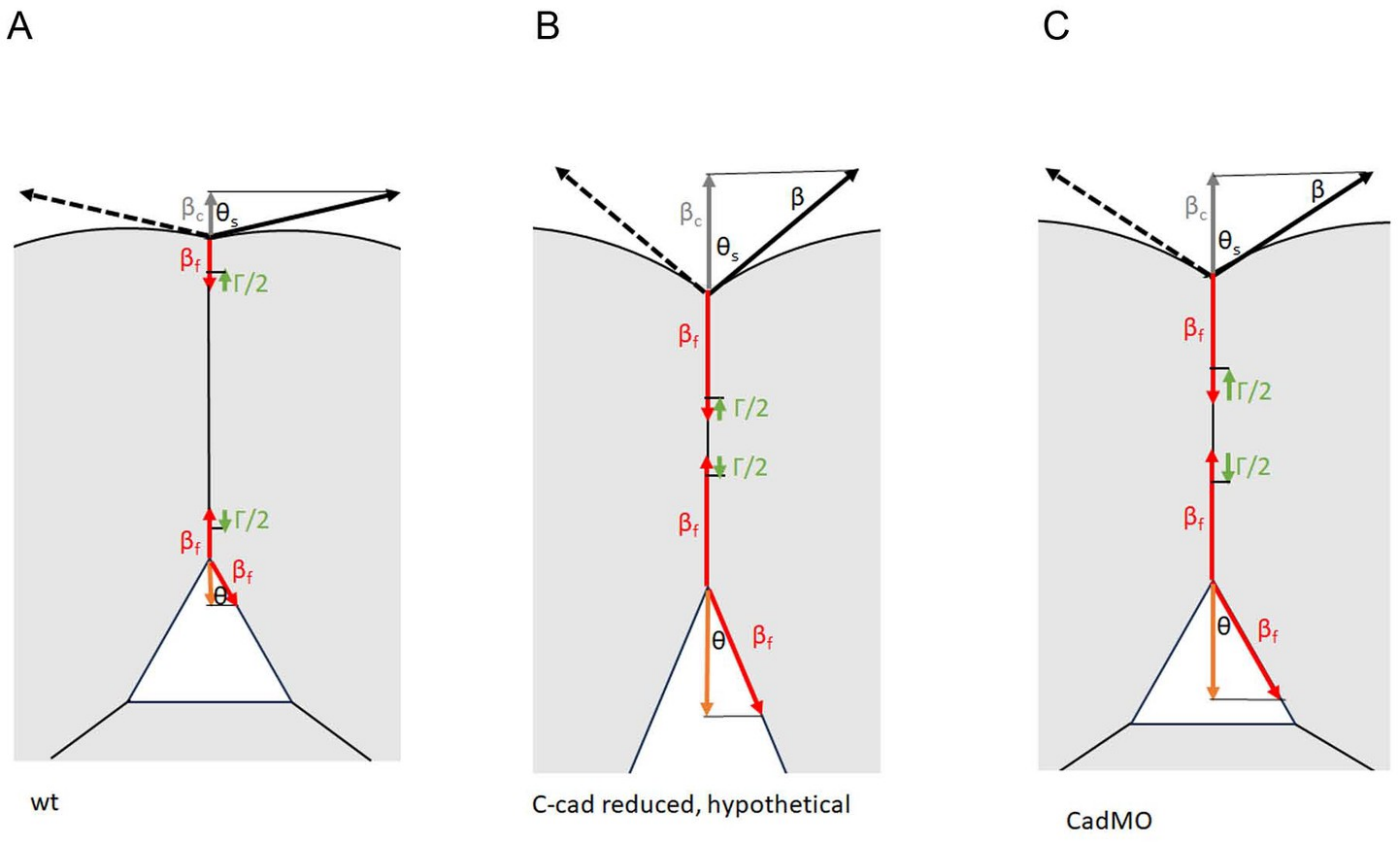
Diagrams schematically depicting the relationships between tensions (shown per cell) and contact angles at tissue surfaces (top) and at interstitial gaps (triangles). (A) In normal CM, cortical tension *β* at the tissue surface (black) is strongly reduced upon cell adhesion to tension *β*_*f*_ (red). Release of binding energy due to adhesion factor interactions at the narrow CM contacts generates an adhesion tension Γ/2 (green). Tensions *β*_*f*_ and Γ/2 balance the resultant tension *β*_*c*_ (grey); surface contact angle, *θ*_*s*_. The same tensions *β*_*f*_ and Γ/2 act at the transition to interstitial gaps, but at the gap surface not *β* but the much smaller *β*_*f*_ balance these tensions (orange), requiring a much smaller contact angle *θ*. (B) In C-cad morphants, tension *β* at the free surface remains; at contacts tension it is much less diminished. In the width range of normal CM, Γ/2 may remain the same, contact angle *θ* becomes smaller, and the relative adhesiveness *α* appears reduced. (C) When the average Γ/2 is increased with contact width much beyond the normal CM range, the contact angle *θ* at gaps can remain the same or even increase while a lowered angle *θ*_*s*_ at the surface still indicates the reduced overall adhesion strength (the difference *σ* = *β* − *β*_*c*_) due to C-cad depletion.

The average relative adhesiveness *α* at gaps in normal and in different adhesion factor-depleted CM is not correlated with contact abundance. To understand this unexpected result, we ask how relative adhesiveness *α* is related to a measure of absolute adhesion strength, the tissue surface tension *σ*, which has been determined for *Xenopus* gastrula tissues including the CM [16, 17, 38, 39]. It corresponds to the difference between the tensions at the free tissue surface, *β*, and at cell contacts, *β*_*c*_. *β* is essentially the tension generated by the contractile cell cortex, and *β*_*c*_ is determined by a cadherin-dependent downregulation of this cortical tension to a within-tissue level *β*_*f*_ and an oppositely oriented adhesion tension Γ/2, the binding energy released per unit area and per cell upon adhesion (Fig 9A) [26, 40]. Thus, *σ* = *β* − (*β*_*f*_ − Γ/2), with *β* − *β*_*f*_ being large compared to Γ/2 in gastrula tissues and cortex downregulation dominating adhesion strength [16, 25]. The contact angle *θ*_*s*_ at the tissue surface is given by cos *θ*_*s*_ = (*β*_*f*_ − Γ/2)/*β* (Fig 9A). Importantly, at the free surface of interstitial gaps, tension does not return to the tissue surface level but remains at *β*_*f*_ [25]. Tension at contacts around gaps is again *β*_*c*_ = *β*_*f*_ − Γ/2, and adhesion strength at gaps, relevant for the attachment and detachment of cells within the tissue, is *σ*_*i*_ = *β*_*f*_ − (*β*_*f*_ − Γ/2) = Γ/2, which in the gastrula is small compared to cortical tensions. Accordingly, tension equilibrium requires a contact angle *θ* much smaller than that at the tissue surface: cos *θ* = (*β*_*f*_ − Γ/2)/*β*_*f*_ (Fig 9A).

For the strength of C-cad mediated adhesion, quantitative data are available. Depleting C-cad reduces absolute adhesion strength *σ* in gastrula tissues by about half: the tension *β* at the tissue surface is less reduced at contacts within the tissue, i.e. *β*_*c*_ remains relatively high and thus the difference *σ* between a higher *β*_*c*_ and an unchanged *β* is smaller [16]. At an unaltered adhesion tension Γ/2, this would decrease contact angles *θ* at gaps and thus *α* (Fig 9B), which represents the situation of diminished *α* in the < 250 nm width range in C-cad morphants (see Fig 7B). However, most contacts are much wider in these morphants, and as *α* increases in proportion to *w*, the average *α* for *w* > 250 nm is much higher, implying that Γ/2 is also increasing linearly with *w*. The resulting overall increase of Γ/2 upon C-cad depletion tends to compensate for the diminished reduction of *β* at contacts and the higher *β*_*f*_ (Fig 9C). Quantitatively, from *β*_*c*_ = 0.06 mJ/m^2^ in normal CM [16], we calculate that *β*_*f*_ = 0.07 mJ/m^2^ and Γ/2 = 0.01 mJ/m^2^, values which are almost identical for ectoderm [25]. Assuming for C-cad morphants a similarly increased *β*_*c*_ = 0.15 mJ/m^2^ in CM as in the ectoderm, *β*_*f*_ would be 0.19 mJ/m^2^ and Γ/2, 0.04 mJ/m^2^. Thus, *β*_*f*_ is 2.5-fold higher but Γ/2 is increased 4-fold in morphants, suggesting overcompensation, as is in fact apparent from the contact angles (see Fig 1K). A similarly increased Γ/2 after C-cad depletion had also been found for the ectoderm [25] but had been left unexplained at the time as the width dependence of *α* had not yet been uncovered.

In summary, at gaps the effect of C-cad knockdown on cortical tensions is compensated by an increase in average adhesion tension Γ/2 due to the widening of contacts in morphants and a proportional increase of adhesiveness. Adhesion strength changes in FN and Has morphants have yet to be examined. The observed reduction of adhesiveness at low widths could in principle occur as in C-cad morphants (e.g. [41]). It could also be due to reduced adhesion tension Γ/2: if the observed increase in contact PCM volume was due to swelling and accompanied by a decrease in PCM density, less interaction between binding sites would take place per PCM-PCM interface area and less binding energy would be released. Adhesion in Syn-4 depleted CM appears very different and may require different concepts for its analysis.

### A mechanism of pericellular matrix-based cell-cell adhesion

To mediate cell-cell adhesion, PCM surfaces have been proposed to interact molecularly in a narrow contact zone by the limited interdiffusion of chain ends and the exchange of non-covalent binding interactions [6]. With only a thin slice at the surface of PCMs involved, adhesiveness would be independent of total PCM thickness and hence of contact width (Fig 8F). For *w* < 100 nm, this is indeed seen for the line tracing *α* frequency peaks, but for this line at *w* > 100 nm and for the whole low-boundary line, the linear increase of *α* with *w* implies that adhesive interaction increases with the thickness of the PCM, most simply achieved by the interpenetration of the PCMs of two cells. Complete interpenetration of the glycocalyx layers has been observed at erythrocyte-macrophage contacts [42]. In the gastrula, the observed change in LSM layer thickness at transitions between contacts and gaps is consistent with such a process.

Interpenetration of coherent PCM meshworks, molecule by molecule, over hundreds of nanometers, seems unlikely. To estimate the diameters of putative interpenetrating units in the contact plane, we assume that the units adhere on all their sides, and that the slope (Δ*α*/Δ*w*) of the *α*-*w* curve is thus proportional to the density of unit-unit contacts, i.e. to twice the inverse of the diameter *d* of a unit, Δ*α*/Δ*w* ∼ 2/*d*. Further, the slope should be proportional to the basic PCM-PCM adhesiveness *α*_0_, as seen in the absence of interpenetration, and thus Δ*α*/Δ*w* = 2*α*_0_/*d*. For calculations the lower-boundary regression line provides the best data. Here *α* → *α*_0_ for *w* → 0 (Fig 8F) and with *α*_0_ and Δ*α*/Δ*w* read off from the *α*-*w* curves (Fig 7), *d* can be calculated as between 130–235 nm, except for Syn-4MO (S2 Table). The main peaks from the *α* distributions provide only few, low resolution data points for *α*-*w* curves but between 100 nm and 600 nm the combined data are consistent with a linear increase, in proportion to the lower-boundary line. Lines corrected for contact angle distortion are constructed by proportionally increasing the original lines by a factor *k* and will thus also give the same *d* values. Minor peaks in the *α* frequency distributions may indicate other contact types in a certain width range, with higher basic adhesiveness.

The estimated *d* values are an order of magnitude larger than the deduced endothelial glycocalyx molecule spacing of ∼20 nm but agree with a 100 nm periodicity that reflects the size of whole glycocalyx bushes [43, 44], and with the sizes of minimal LSM units identified here. Interdigitation of such units is apparent in prechordal mesoderm where glycocalyx II bushes from opposite membranes intercalated in antiparallel fashion at the transition between interstitial gap and adhesive contact [4]. Contacts wider than 200 nm are not labeled with La^3+^ in the CM, but the linear *α*-*w* relationship is maintained, predicting that non-labeled PCM also consists of similarly sized structural building blocks, or PCM stubs, which can interdigitate for adhesion. In summary, the stub interdigitation model assumes a thin PCM-PCM interaction zone, determined by short-range molecular interactions, whose surface is increased by interdigitation equivalent to an effective large-scale folding, such that adhesion tension per cell surface area, Γ/2, increases with PCM width (Fig 8F).

Intercalation unit diameter *d* of Syn-4 morphants differs strongly from that of other morphants, suggesting a unique contact structure. Overall contact and LSM contact abundances, average relative adhesiveness *α*, and the *α* value distribution are also different. At the same time, cell shape differs fundamentally. In a single step, by the depletion of Syn-4, a spindly, serrate mesenchymal cell outline is attained which is common in migratory embryonic tissues of other vertebrates [45–48] but not in amphibian gastrulae. Remarkably, early gastrulation movements including the well-studied convergent extension of the CM are not affected by this transformation whose cellular basis remains to be elucidated. However, mild axis defects caused by Syn-4 depletion can be detected later in development, hours after gastrulation, and these are to a large extent characterized by defective neural tube extension and closure [14].

Shedding of whole plaques but also of isolated stubs suggests that stubs are capable of reversible lateral adhesion. This raises the question of how the interdigitation of stubs from opposite membranes is favored over their lateral association as plaques on the same membrane. Possibly, their antiparallel lateral attachment during interdigitation releases more binding energy than parallel contact. Being linked to the cortical cytoskeleton [44], stubs within plaques could also be actively separated to facilitate interdigitation, thus controlling the initiation of adhesion. Conversely, active lateral compression in existing contacts could promote de-adhesion and cell separation.

Generally, controlled deployment of PCM materials at contacts and in gaps and regulation of PCM height and density may determine contact abundance and adhesion, and be essential for proper cell movements. Overproduction of PCM in C-cad, FN and Has morphants could clog the extracellular space, render cell surfaces non-adhesive and impede cell migration and rearrangement in multiple gastrula regions. By contrast, the sparse PCM in Syn-4 morphants could permit accelerated movements which may depend on cadherin functions as gastrulation is inhibited in Syn-4/C-cad double morphants. Such general mechanical effects of adhesion factor depletion may be considered as alternative or complementing explanations for gastrulation defects which so far are often attributed to aberrant cell signaling alone. On the other hand, while upon adhesion factor depletion some contact types seem to be modified but remain adhesive, others become non-adhesive and still others may disappear. Such lowering of contact abundance without affecting the average adhesion strength of remaining contacts may also contribute to gastrulation defects.

## Supporting information

S1 Fig Frequency distributions of contact widths. (A–E) Contact width spectra for normal CM (A), from Barua et al. [4], and for CM morphants (B–E). Contact widths abundances were collected in 50 nm width bins. (A’) The difference spectrum comparing normal CM and ectoderm shows the signature of glycocalyx I (encircled), suggesting absence of this contact type in the CM. (B’–E’) Difference spectra comparing CM morphants to normal CM (the spectrum for normal CM subtracted bin by bin from respective morphant spectra).
(TIF)

S2 Fig Width frequency distributions of triple-layered (LSM-unlabeled-LSM) contacts. Wt, 101 measurements from 4 TEM images; FN knockdown (FNMO) 107 measurements from 8 TEM images; Has1 knockdown (Has1MO) 138 measurements from 8 TEM images. No triple-layered contacts were seen in C-cad or Syn-4 morphants.
(TIF)

S3 Fig Frequency distributions of *α* values at different widths. (A–E, A’–E’) Each 0–100 nm width bracket in Fig 8 is broken up into a 0–50 nm and a 50–100 nm bracket, respectively. (A’’–E’’, B’’’–E’’’) Distributions for larger widths brackets not shown in Fig 8. Treatments and width brackets indicated on top of each diagram. n, number of *α*-*w* data points.
(TIF)

S4 Fig Relative adhesiveness *α* plotted as a function of contact width *w* for combined data, to estimate a correction factor *k* for contact angle values. Dotted lines, regression lines for lowest (red) and highest values (green). Dashed lines, corrections for contact angle distortions due to random orientation of sectioning planes, as described in the Methods section. *k* = 4/3 is compatible with the intersection of the corrected lines just beyond the *α*-*w* distribution. Equations for lines are indicated as in Fig 7.
(TIF)

S1 Table Relative adhesiveness *α* at low (*w* < 250 nm) and high (*w* ≥ 250 nm) contact widths. Av, average of *α* values; SD, standard deviation; n, number of *α*-*w* data points, i.e. gap-contact transitions; p (MO/wt), p-values for significance morphants vs. untreated CM (wt); p (low/high), p-values for significance low vs. high contact widths.
(PDF)

S2 Table Regression line parameters. Regression lines delineating the lower boundaries of *α*-*w* distributions were determined as described in the main text. *α*_0_, intersection of regression line with *α* axis; Δ*α*/Δ*w*, slope of regression line; r, regression coefficient; *d*, interdigitation distance *d* = 2*α*_0_/(Δ*α*/Δ*w*). The calculated *d* is comparable to the measured average lengths of the shortest LSM units (“stubs”) in normal contacts (156 nm), FN morphants (128 nm) and Has1 morphants (111 nm) shown in Fig 6F–6H.
(PDF)

S3 Table Positions of first peak in *α* frequency distributions in consecutive width brackets. Frequencies of *α* were binned in 0.025 intervals of *α* and the position of the first peak is indicated as the mid-point of a bin, as read off from Fig 8 and S3 Fig, for width brackets 0–50 nm, 50–100 nm, 100–200 nm, 200–400 nm, 400–800 nm, and 800–1200 nm. The mid-points of these brackets are indicated.
(PDF)

S1 Dataset (ZIP)
