# Supplementary figures and images for: Cell contacts and pericellular matrix in the Xenopus gastrula chordamesoderm
Source: PLoS One. 2024 Feb 12;19(2):e0297420. doi: 10.1371/journal.pone.0297420 (PMC10861091; doi:10.1371/journal.pone.0297420)

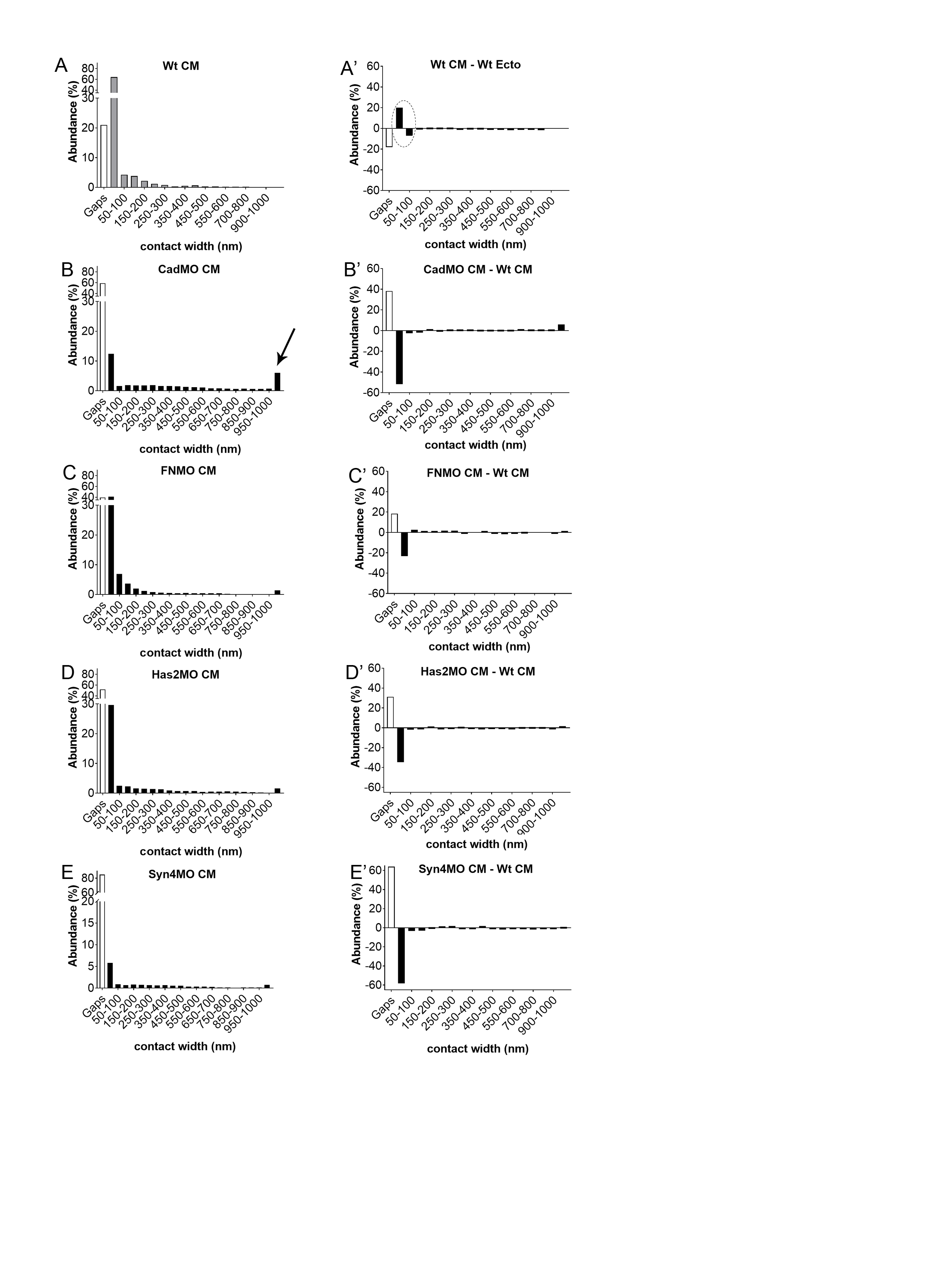

Supplement: S1 Fig — (A–E) Contact width spectra for normal CM (A), from Barua et al. [4], and for CM morphants (B–E). Contact widths abundances were collected in 50 nm width bins. (A’) The difference spectrum comparing normal CM and ectoderm shows the signature of glycocalyx I (encircled), suggesting absence of this contact type in the CM. (B’–E’) Difference spectra comparing CM morphants to normal CM (the spectrum for normal CM subtracted bin by bin from respective morphant spectra). (TIF) [file pone.0297420.s001.tif]

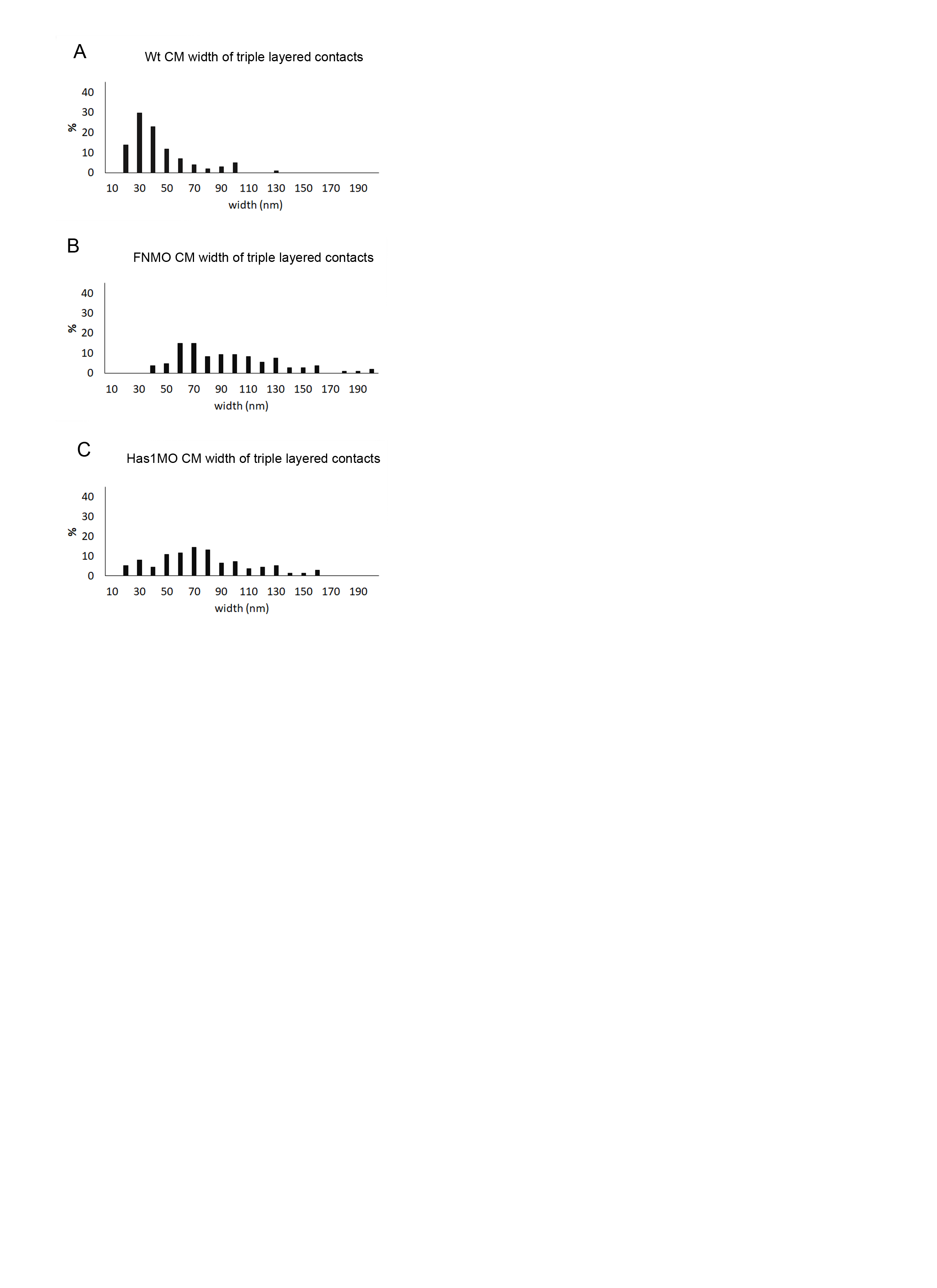

Supplement: S2 Fig — Wt, 101 measurements from 4 TEM images; FN knockdown (FNMO) 107 measurements from 8 TEM images; Has1 knockdown (Has1MO) 138 measurements from 8 TEM images. No triple-layered contacts were seen in C-cad or Syn-4 morphants. (TIF) [file pone.0297420.s002.tif]

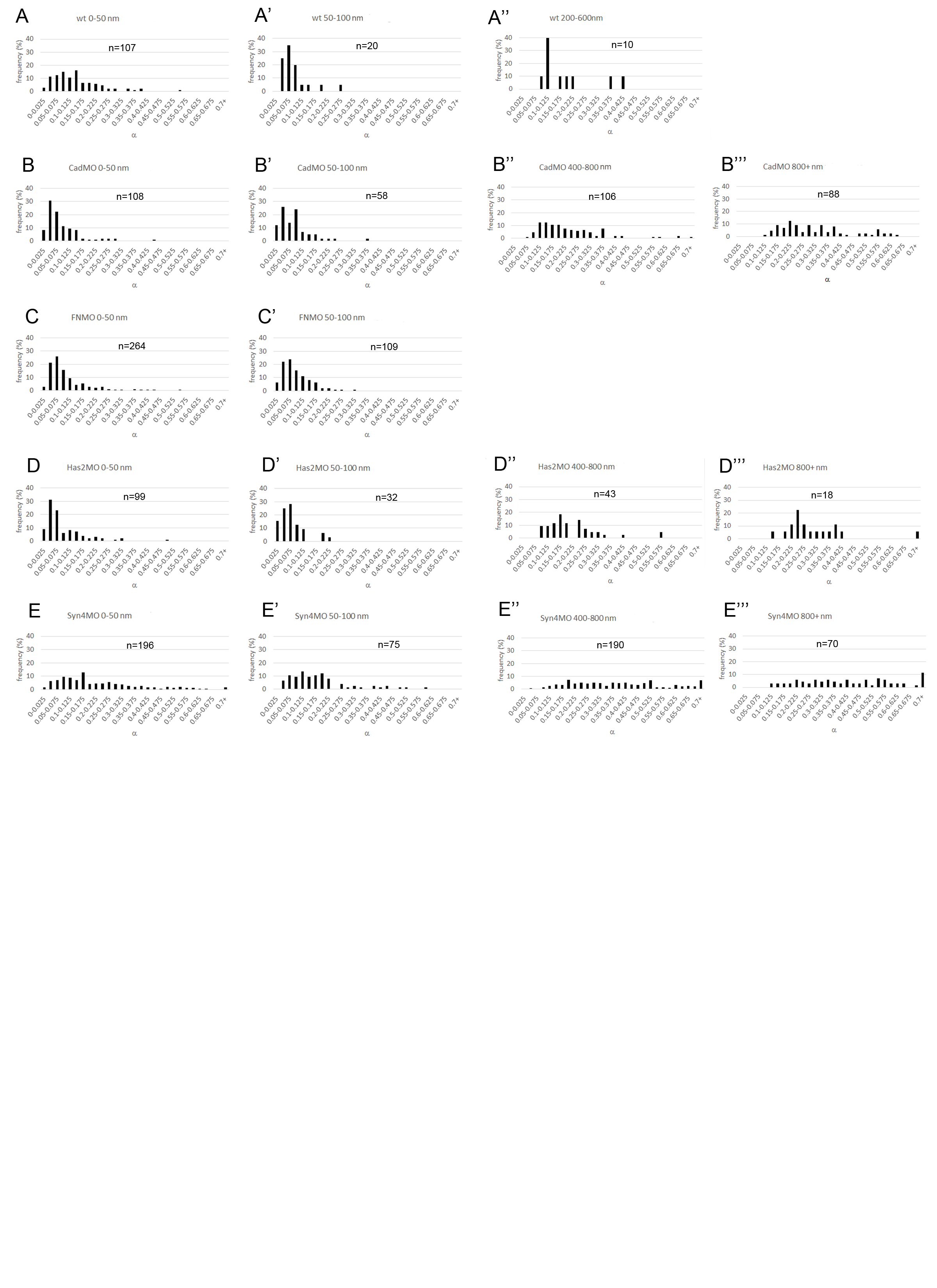

Supplement: S3 Fig — (A–E, A’–E’) Each 0–100 nm width bracket in Fig 8 is broken up into a 0–50 nm and a 50–100 nm bracket, respectively. (A’’–E’’, B’’’–E’’’) Distributions for larger widths brackets not shown in Fig 8. Treatments and width brackets indicated on top of each diagram. n, number of α-w data points. (TIF) [file pone.0297420.s003.tif]

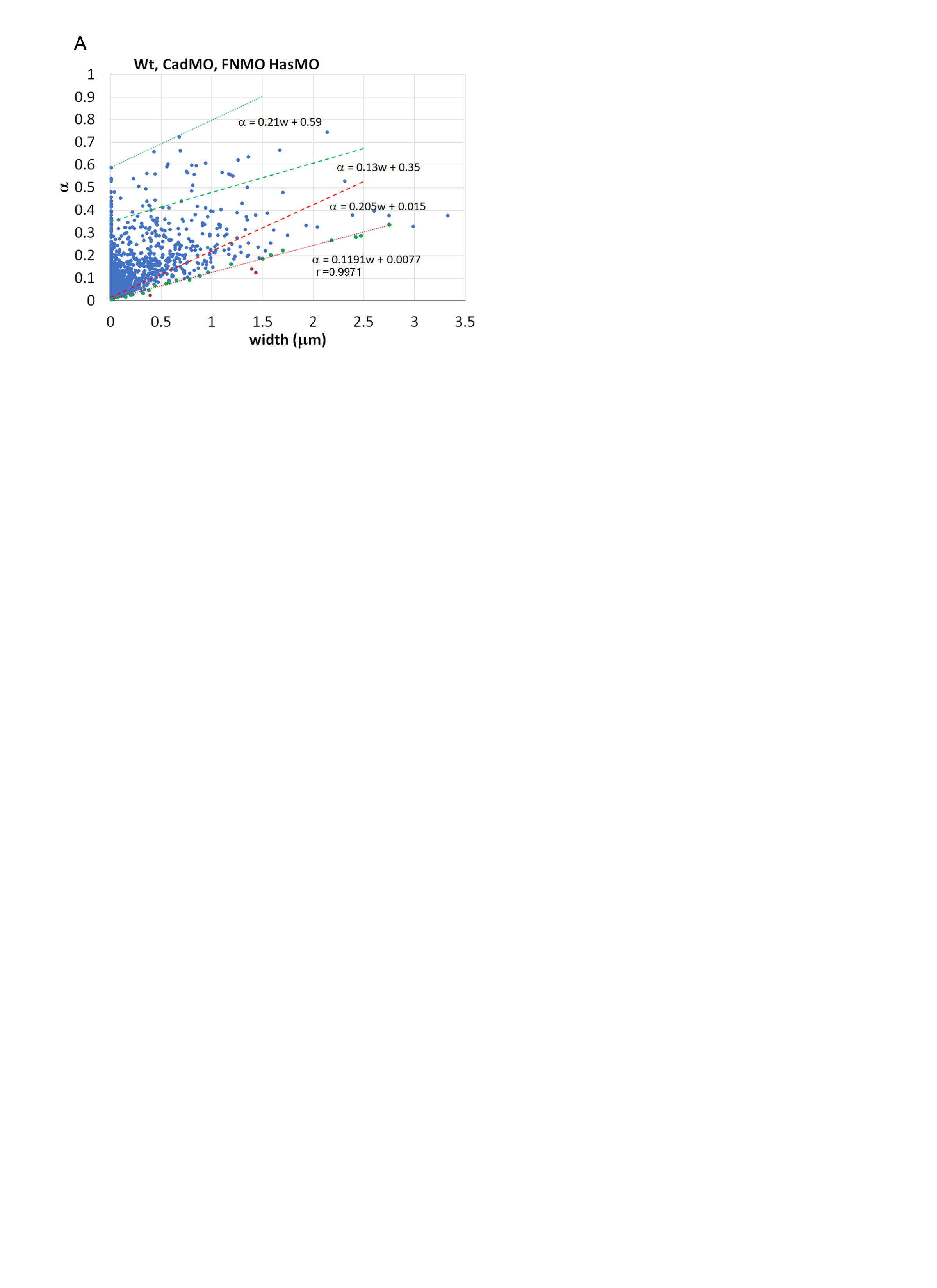

Supplement: S4 Fig — Dotted lines, regression lines for lowest (red) and highest values (green). Dashed lines, corrections for contact angle distortions due to random orientation of sectioning planes, as described in the Methods section. k = 4/3 is compatible with the intersection of the corrected lines just beyond the α-w distribution. Equations for lines are indicated as in Fig 7. (TIF) [file pone.0297420.s004.tif]

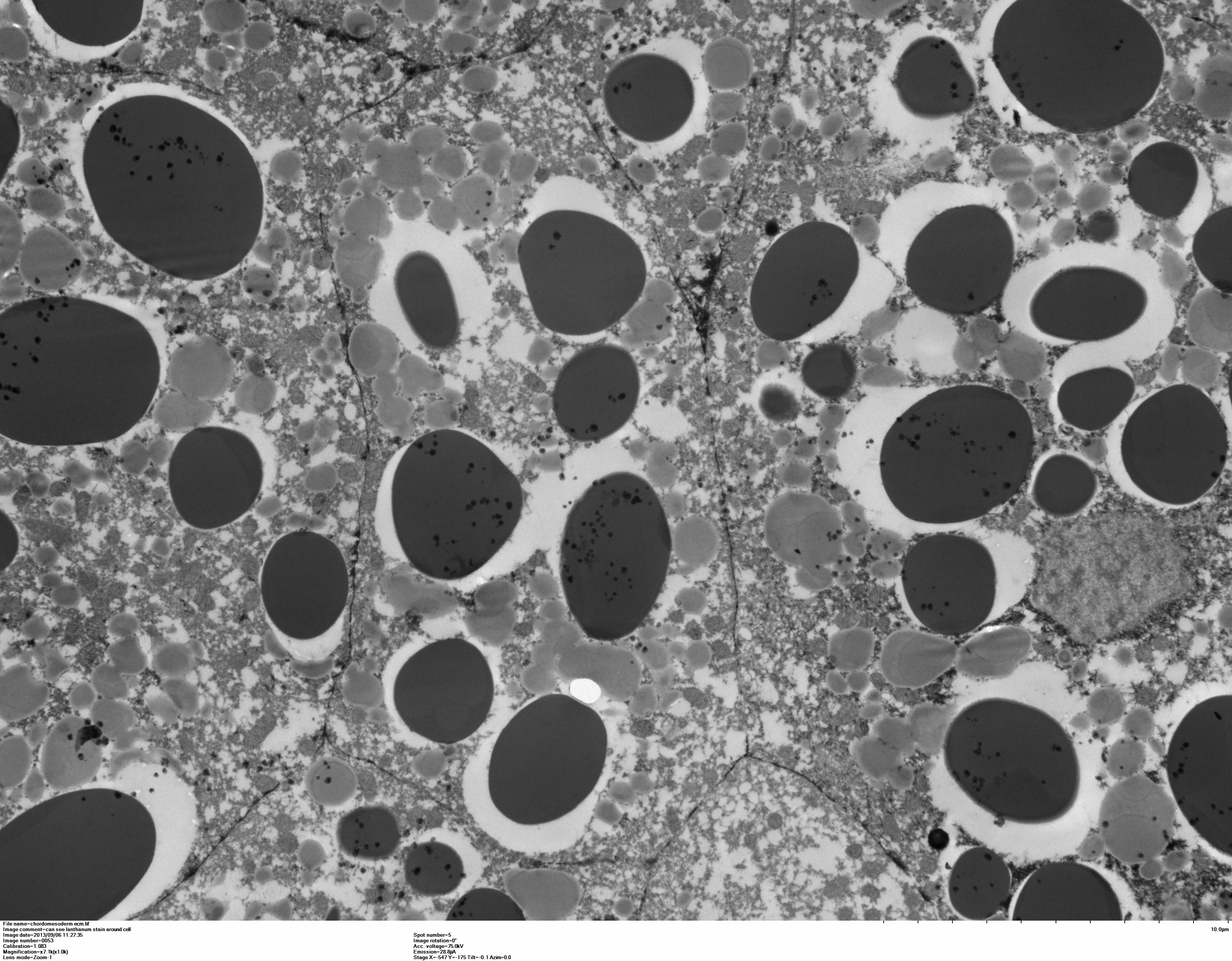

Supplement: S1 Dataset — (ZIP) [file pone.0297420.s008.zip › wt La1.tif]

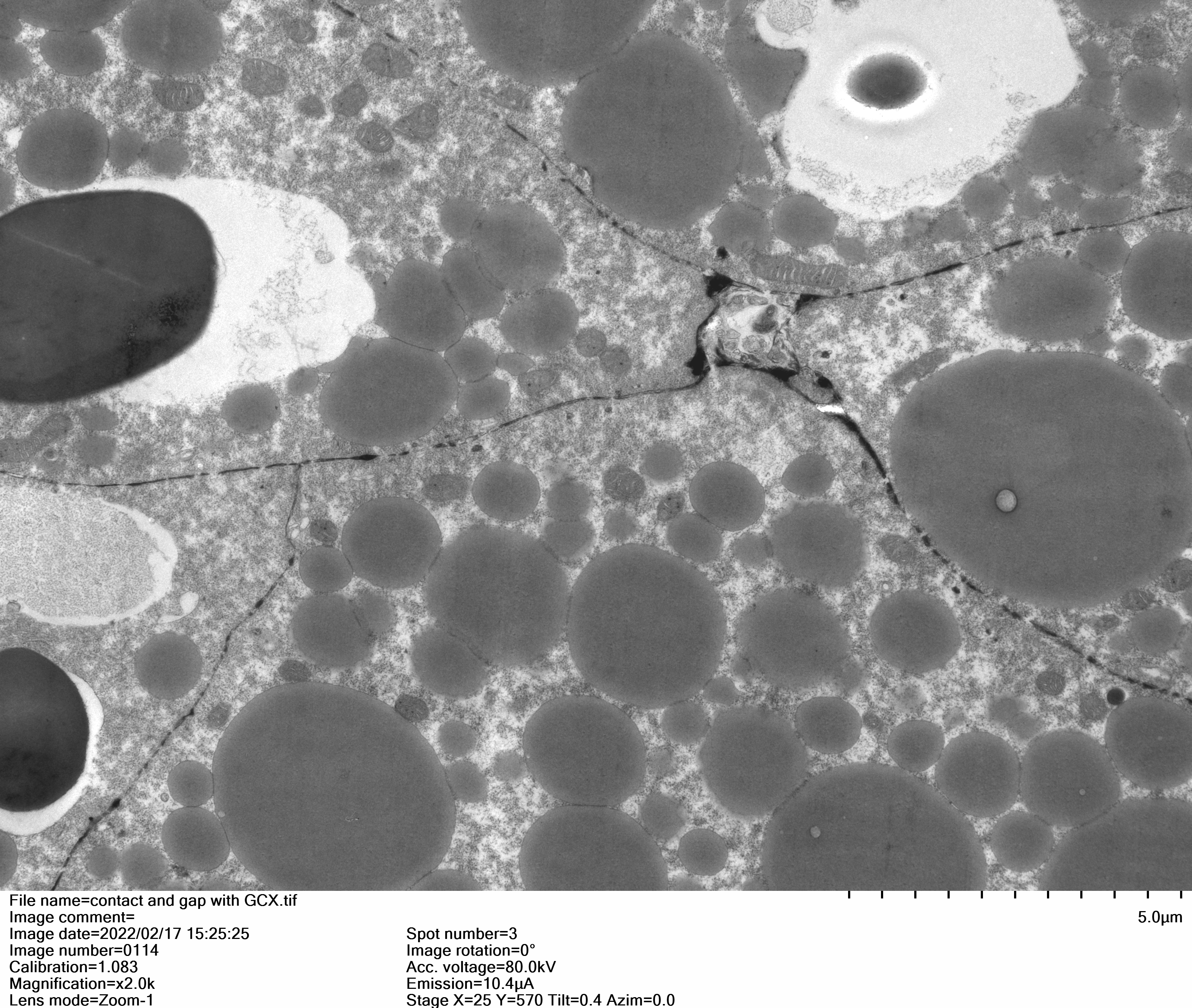

Supplement: S1 Dataset — (ZIP) [file pone.0297420.s008.zip › wt La2.tif]

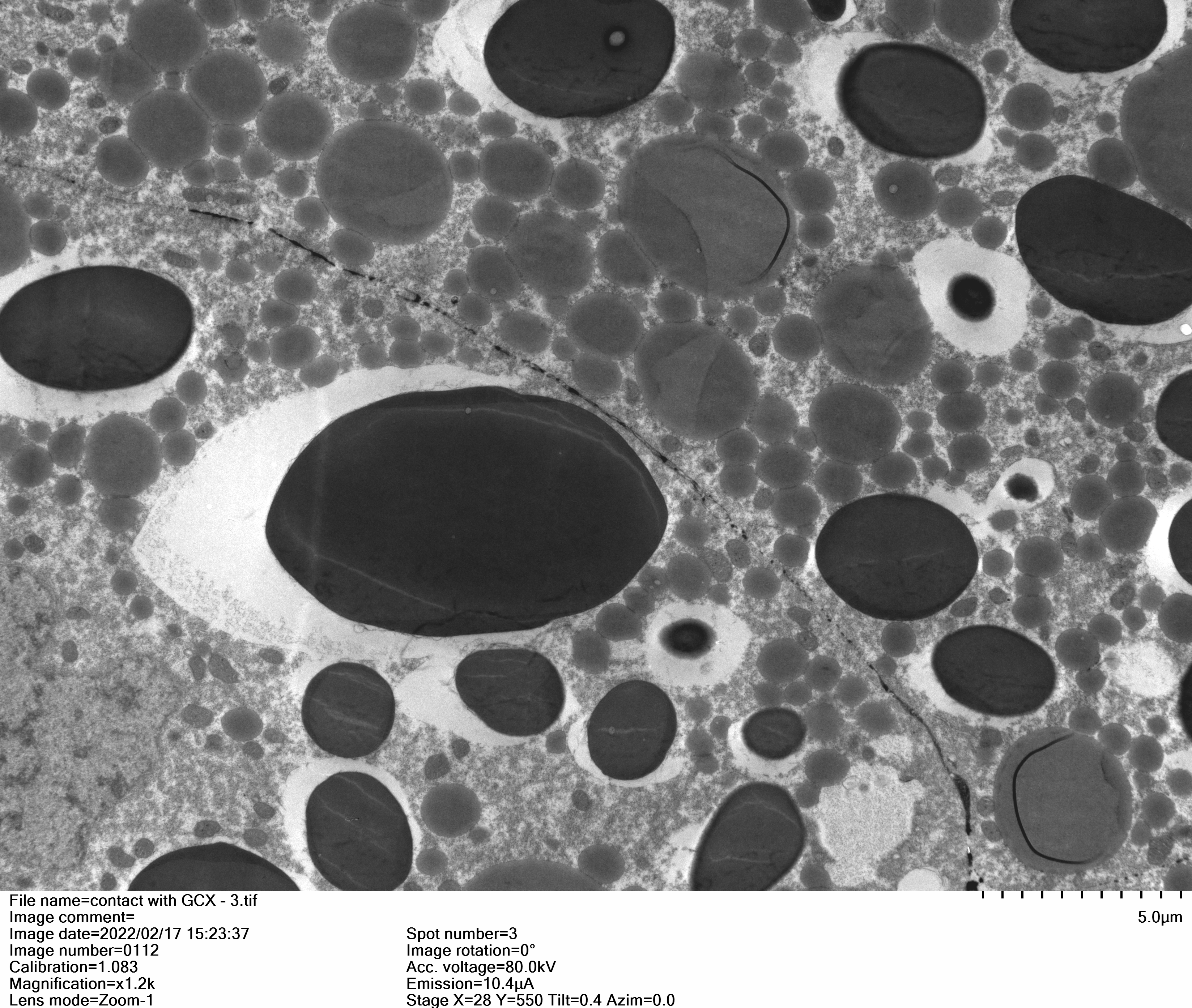

Supplement: S1 Dataset — (ZIP) [file pone.0297420.s008.zip › wt La3.tif]

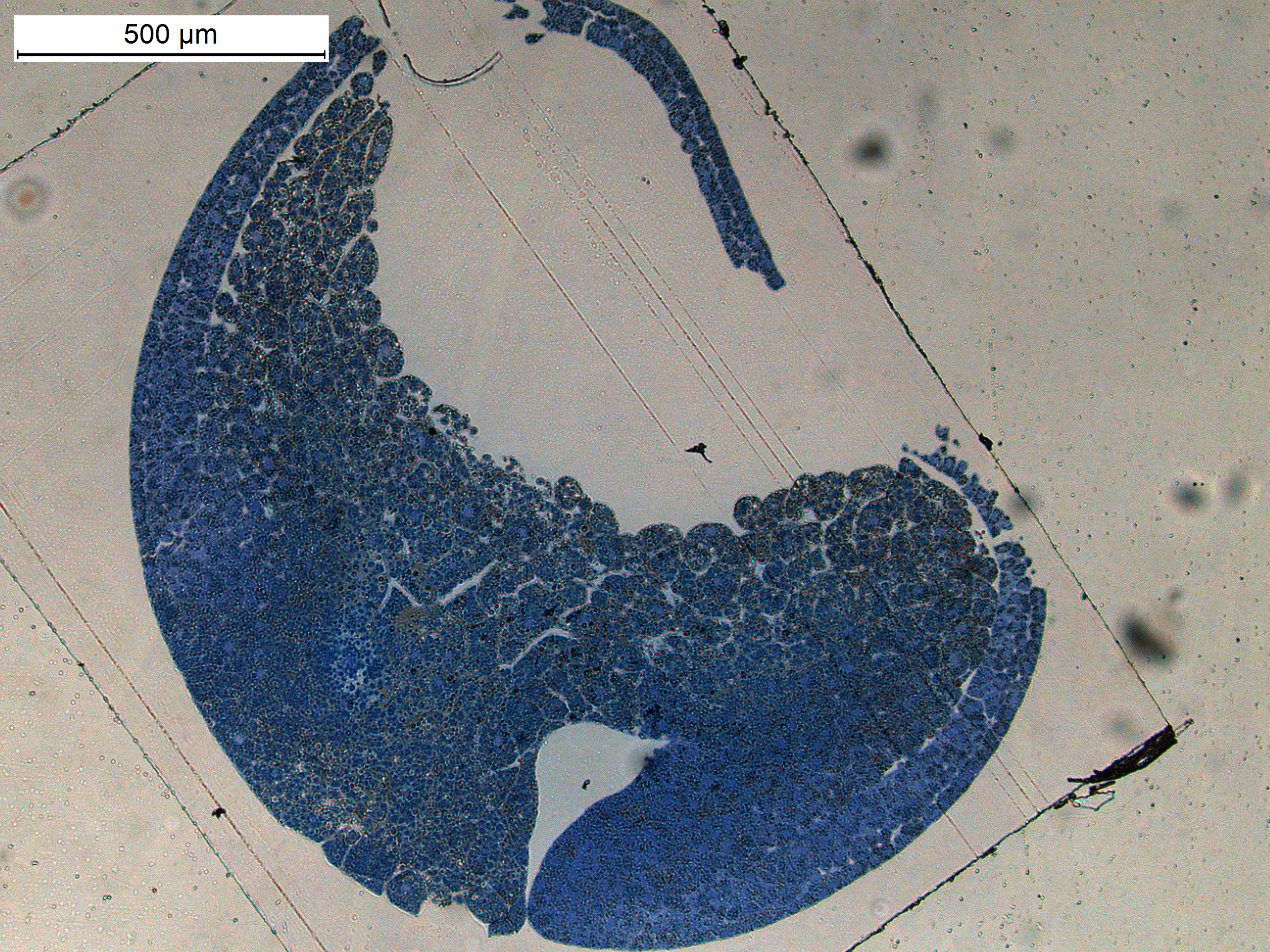

Supplement: S1 Dataset — (ZIP) [file pone.0297420.s008.zip › XWT2.tif]

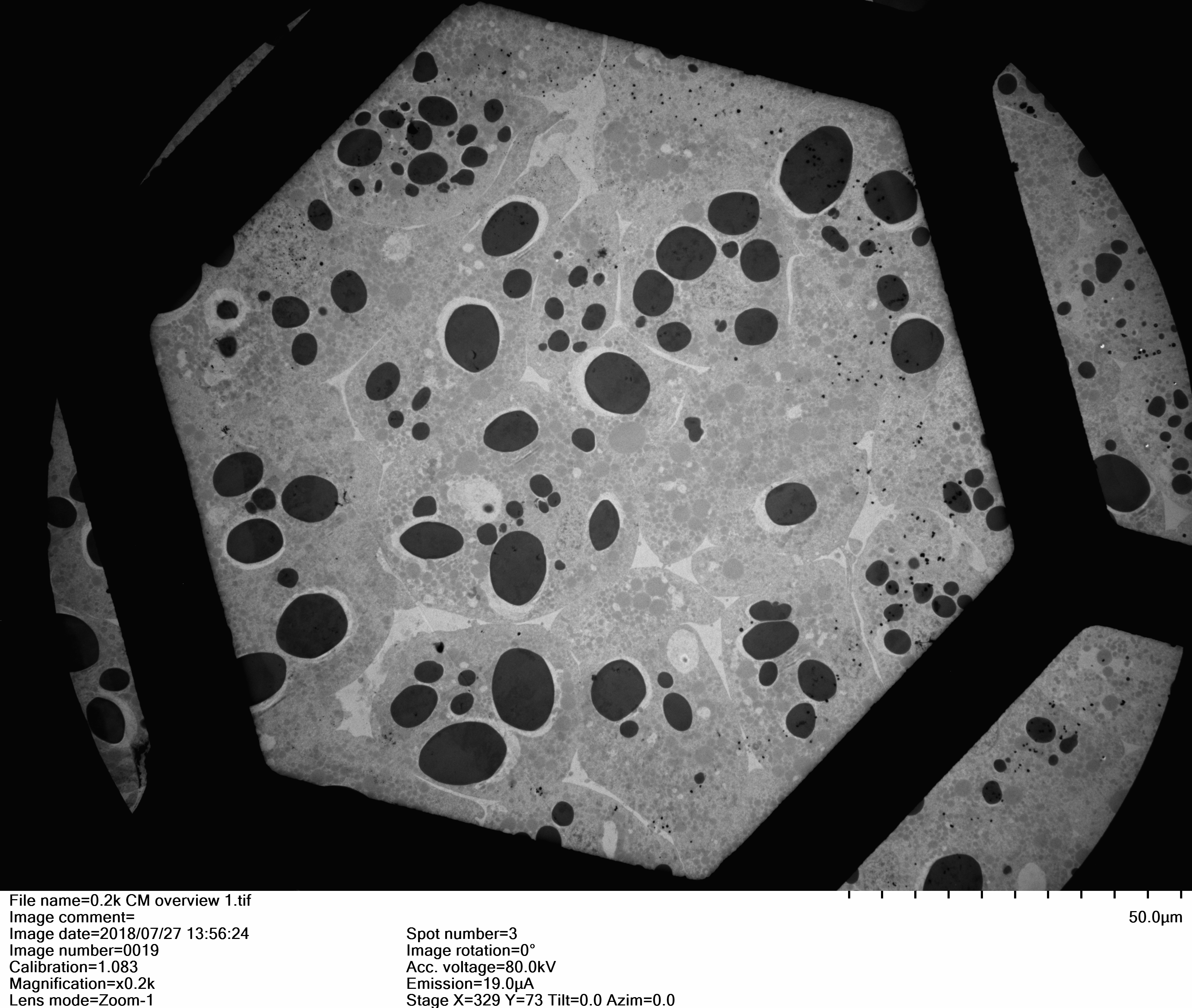

Supplement: S1 Dataset — (ZIP) [file pone.0297420.s008.zip › 0.2k CM wt.tif]

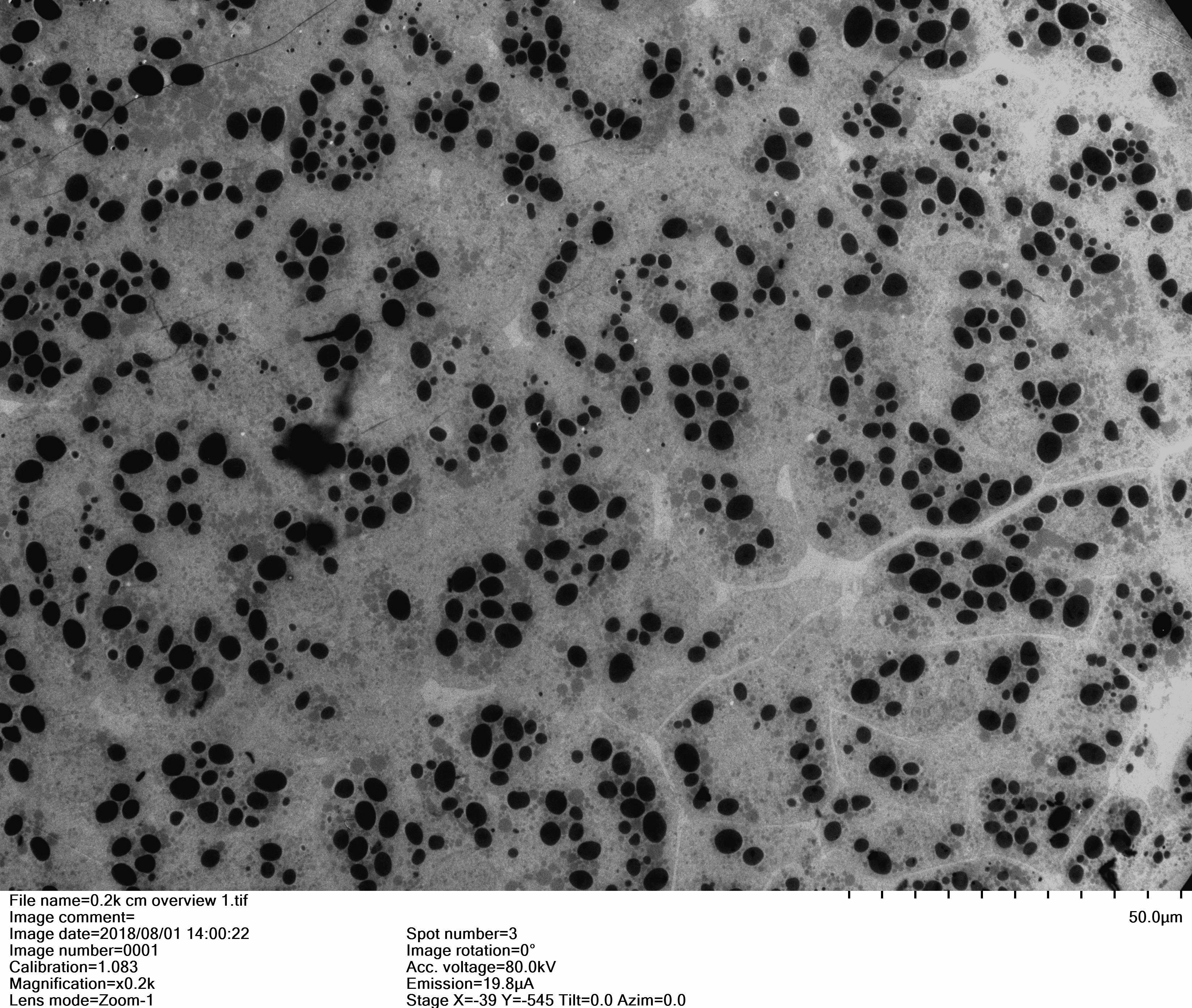

Supplement: S1 Dataset — (ZIP) [file pone.0297420.s008.zip › 0.2k cm wt2.tif]

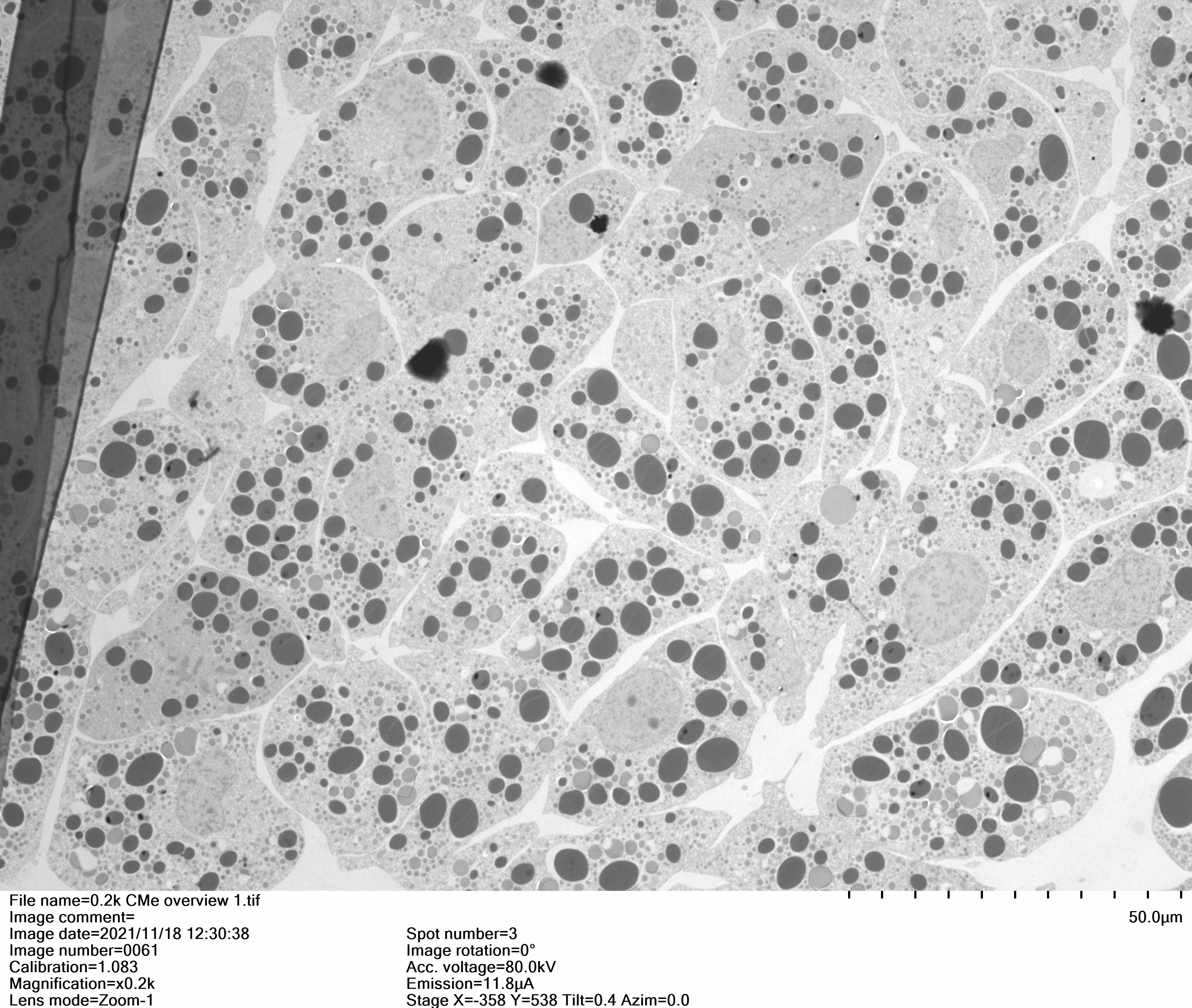

Supplement: S1 Dataset — (ZIP) [file pone.0297420.s008.zip › 0.2k CMe CadMO.tif]

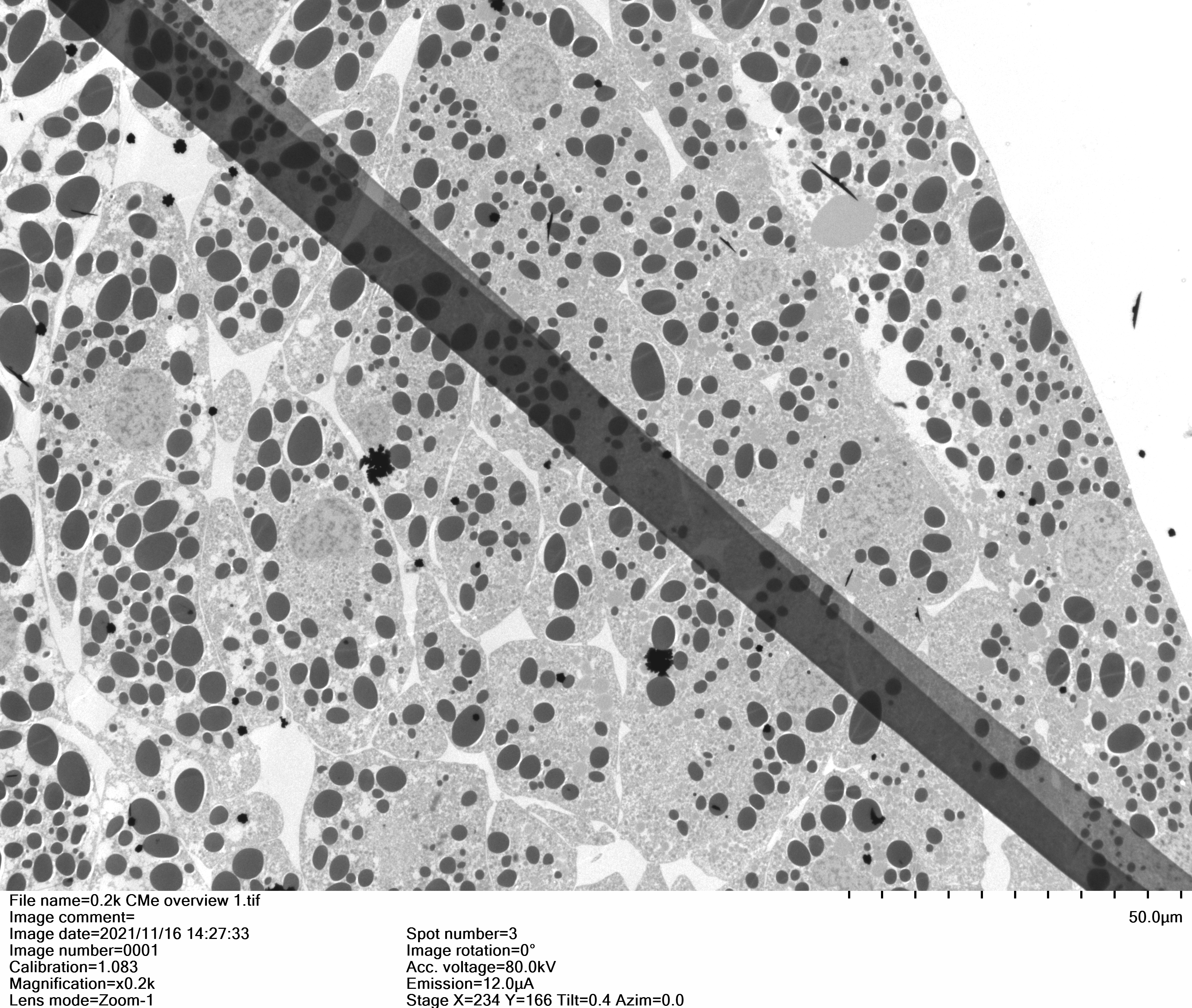

Supplement: S1 Dataset — (ZIP) [file pone.0297420.s008.zip › 0.2k CMe FNMO1.tif]

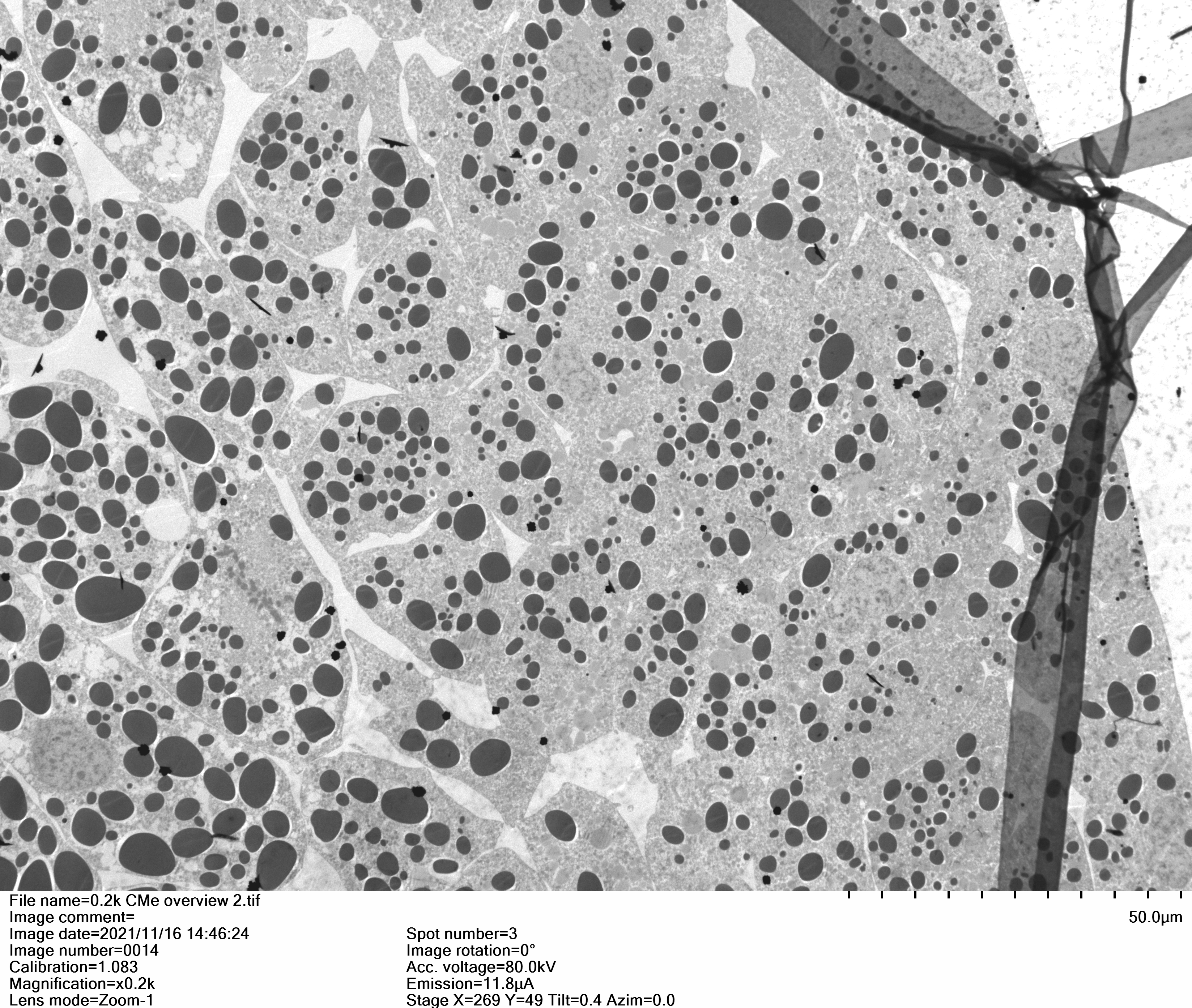

Supplement: S1 Dataset — (ZIP) [file pone.0297420.s008.zip › 0.2k CMe FNMO3.tif]

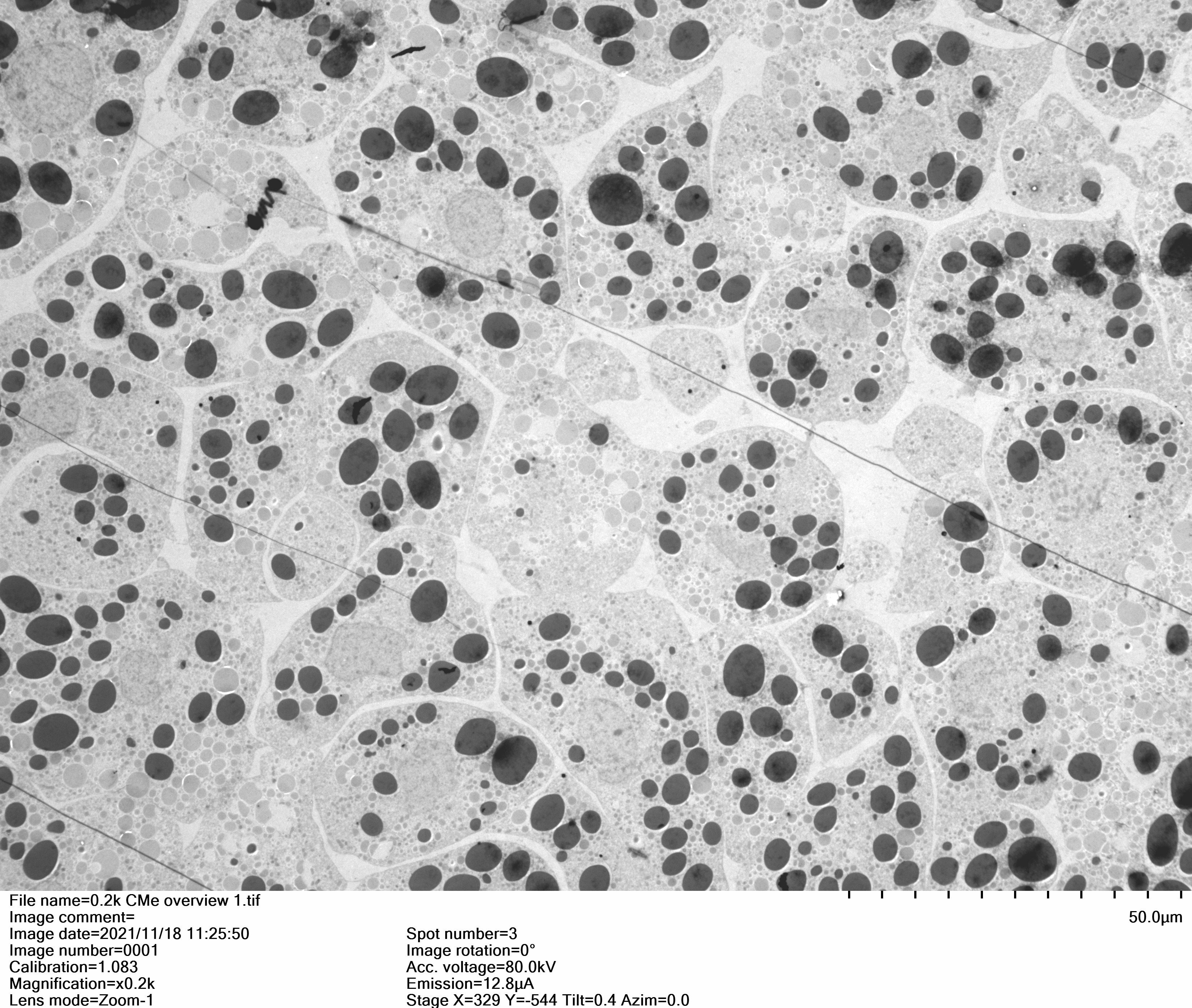

Supplement: S1 Dataset — (ZIP) [file pone.0297420.s008.zip › 0.2k CMeb CadMO.tif]

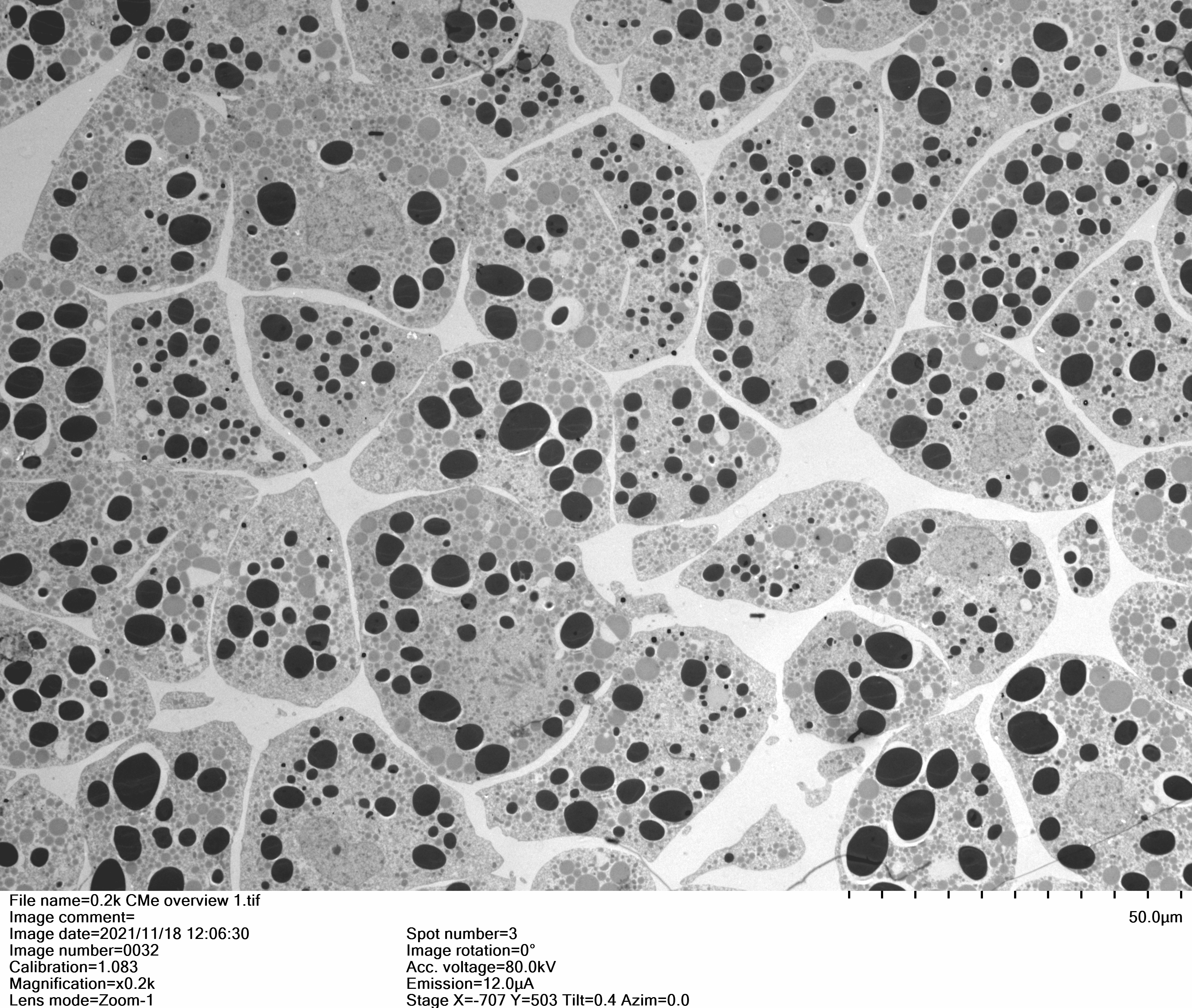

Supplement: S1 Dataset — (ZIP) [file pone.0297420.s008.zip › 0.2k CMec CadMO.tif]

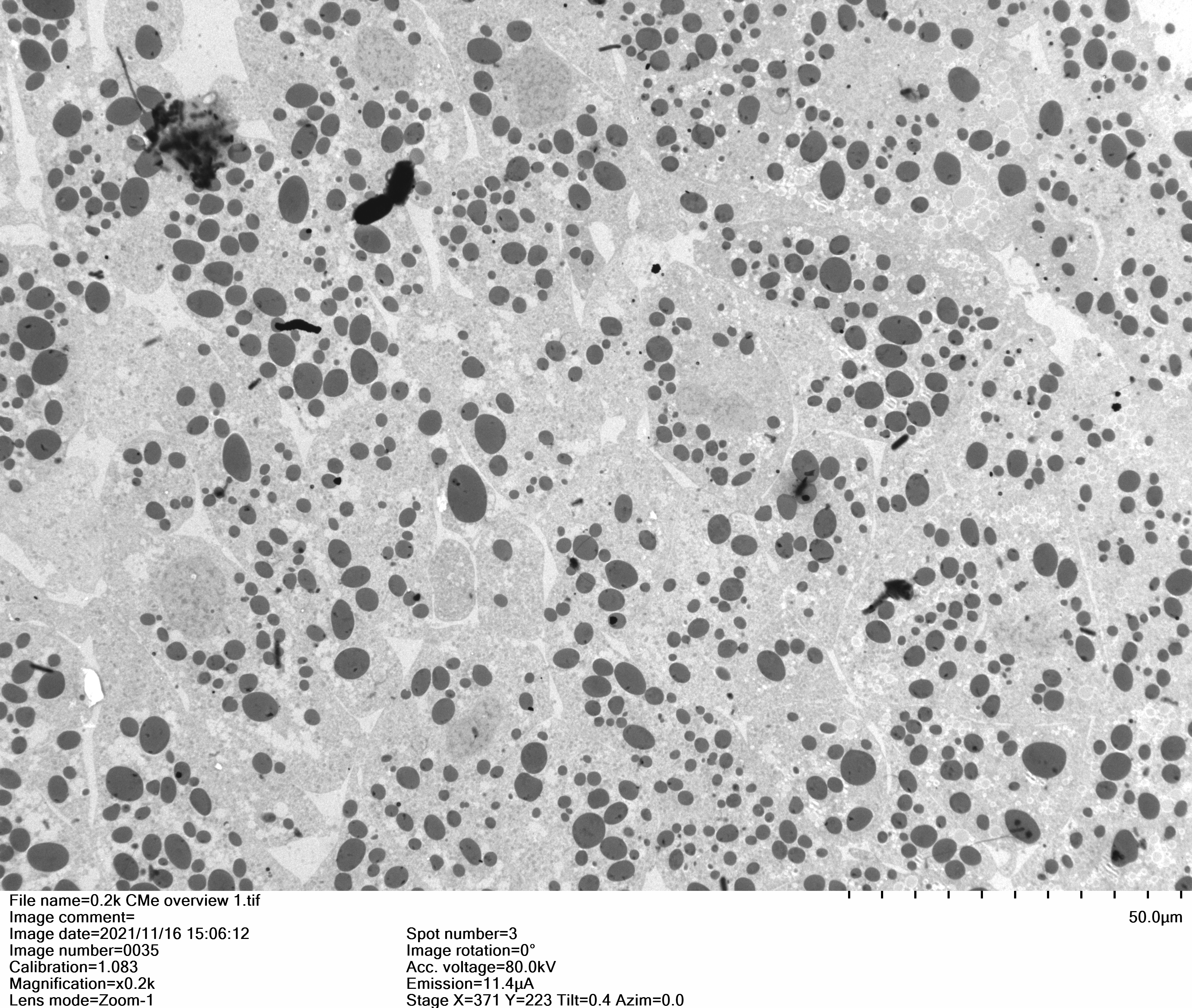

Supplement: S1 Dataset — (ZIP) [file pone.0297420.s008.zip › 0.2k FNMO2.tif]

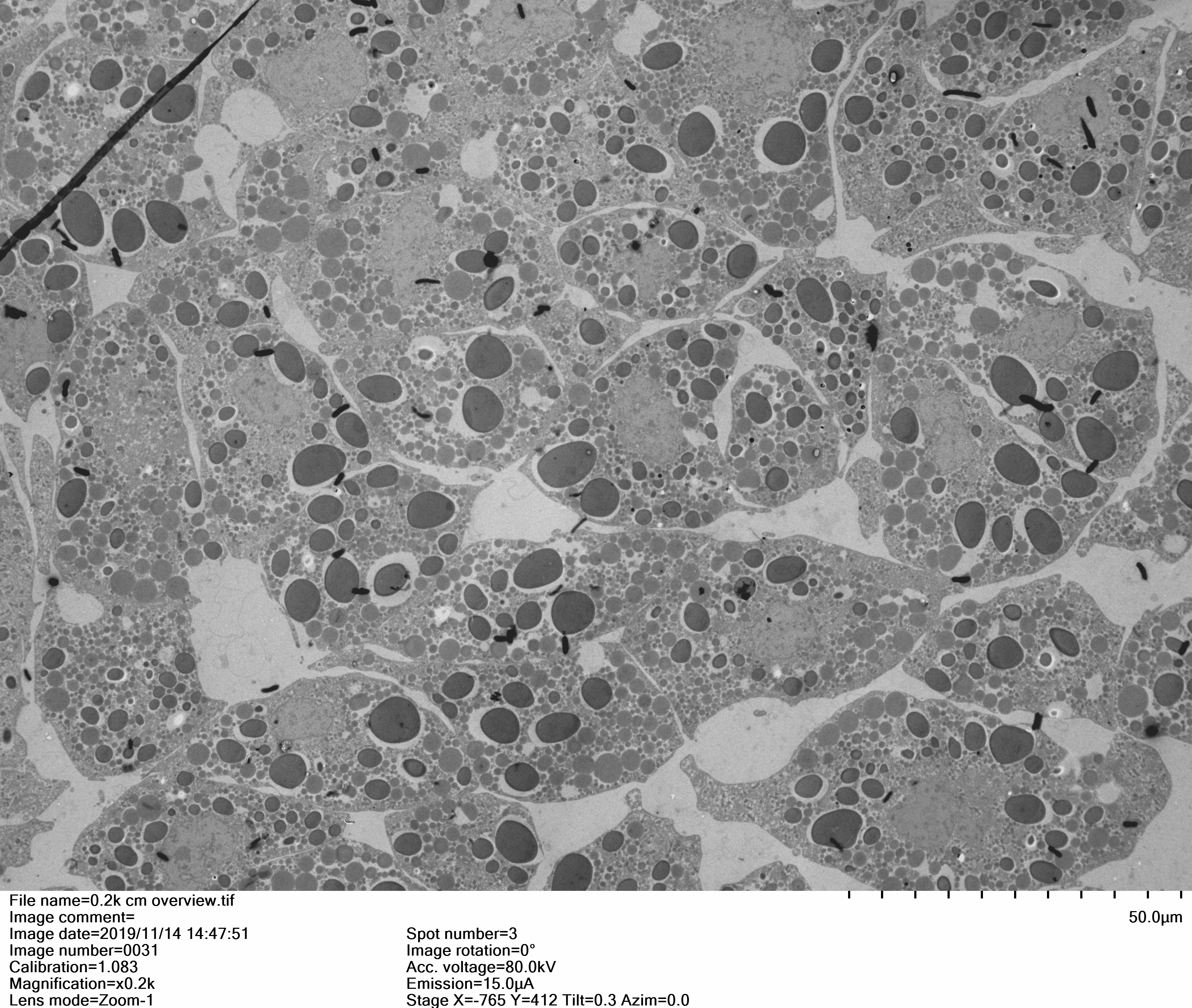

Supplement: S1 Dataset — (ZIP) [file pone.0297420.s008.zip › 0.2k Has1MO.tif]

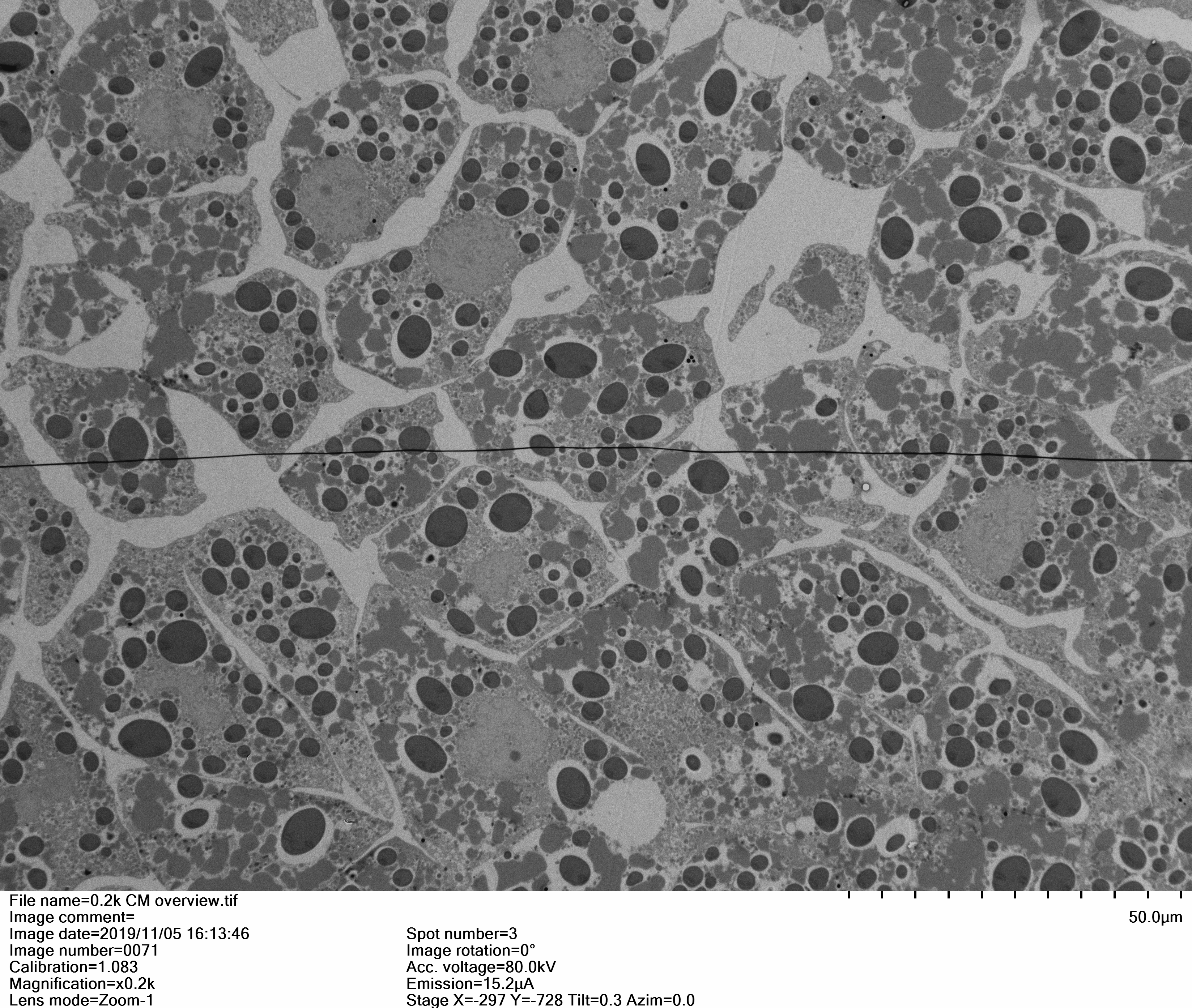

Supplement: S1 Dataset — (ZIP) [file pone.0297420.s008.zip › 0.2k Has1MO2.tif]

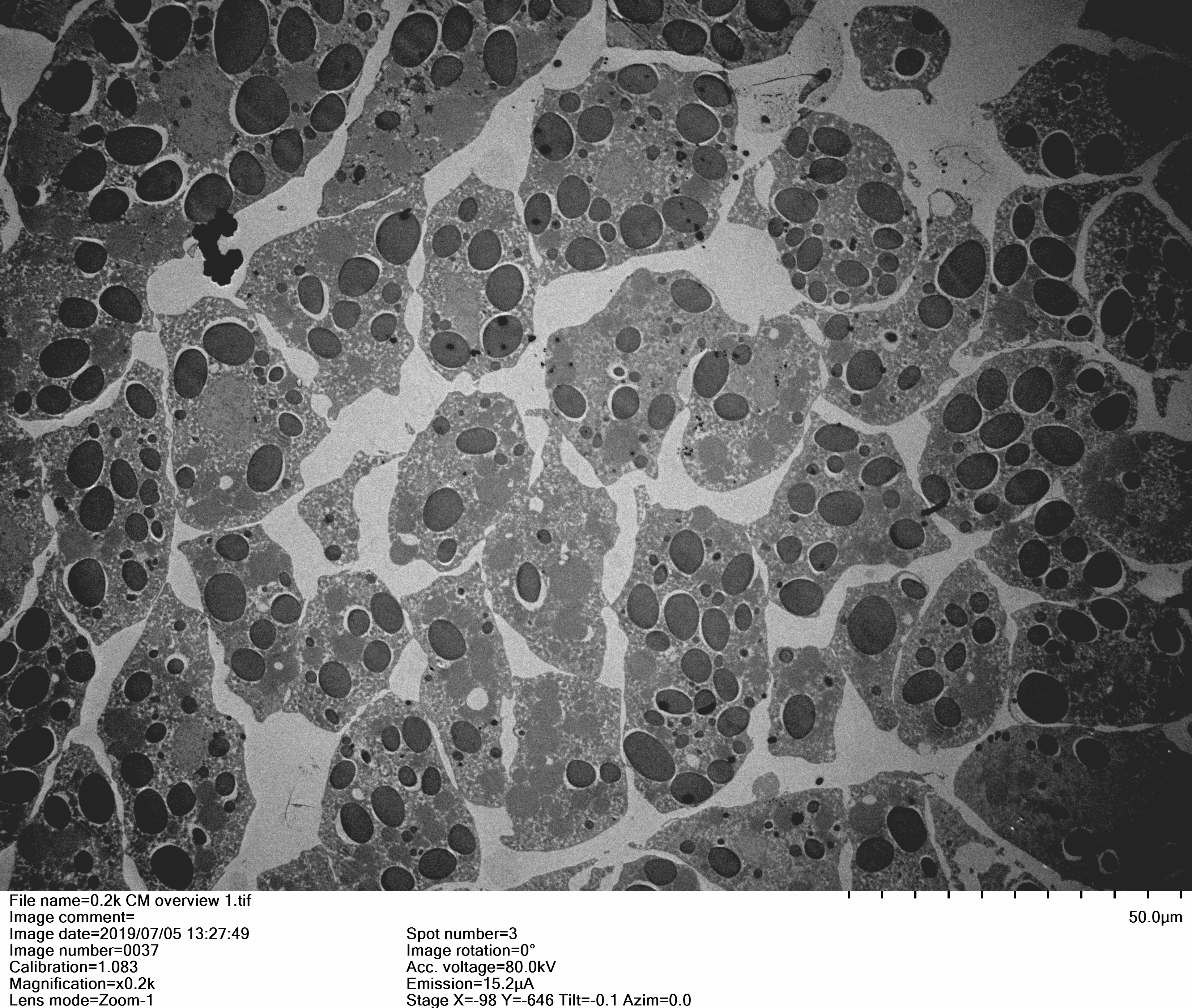

Supplement: S1 Dataset — (ZIP) [file pone.0297420.s008.zip › 0.2k Syn4MO2.tif]

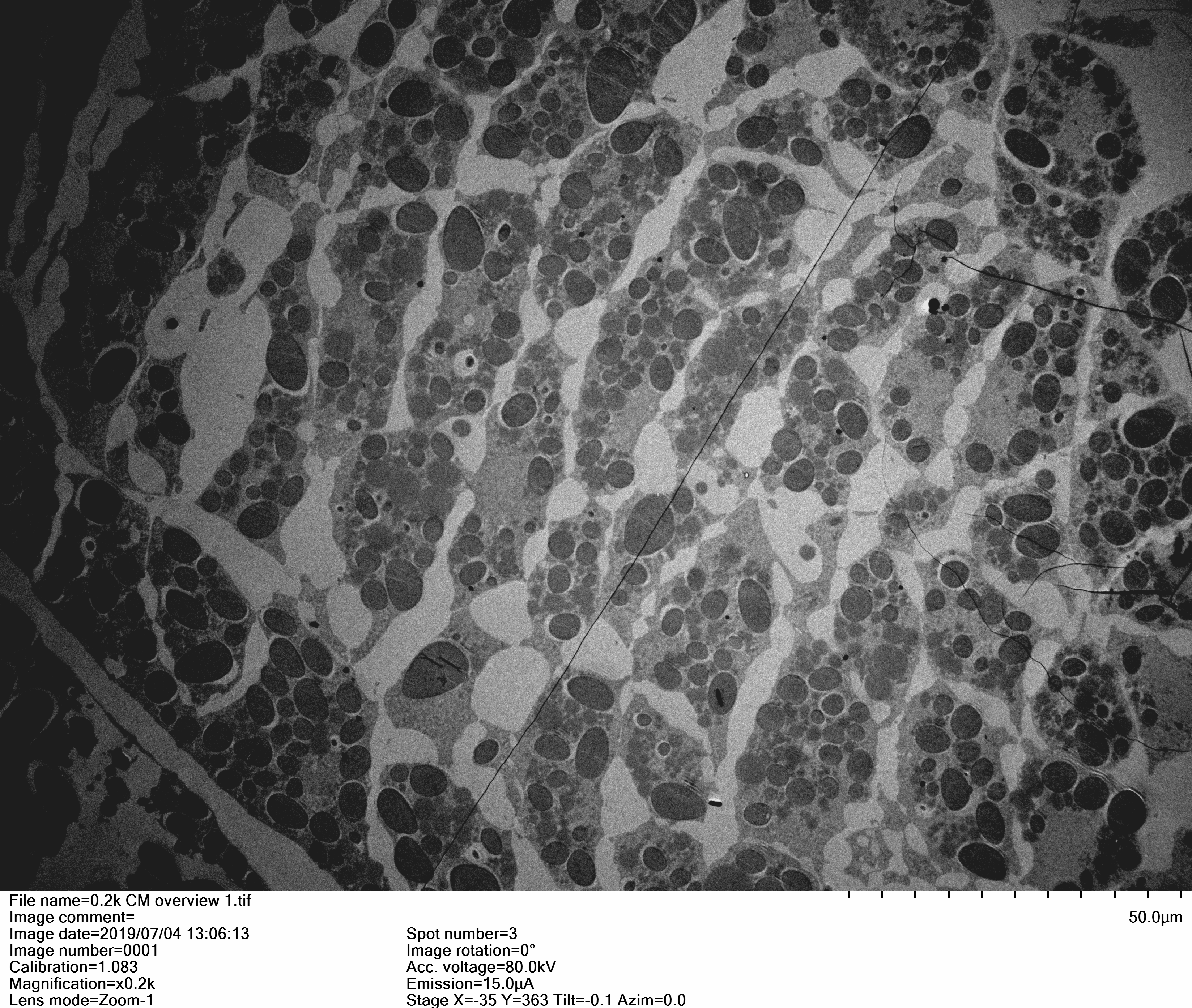

Supplement: S1 Dataset — (ZIP) [file pone.0297420.s008.zip › 0.2k Syn4MO3.tif]

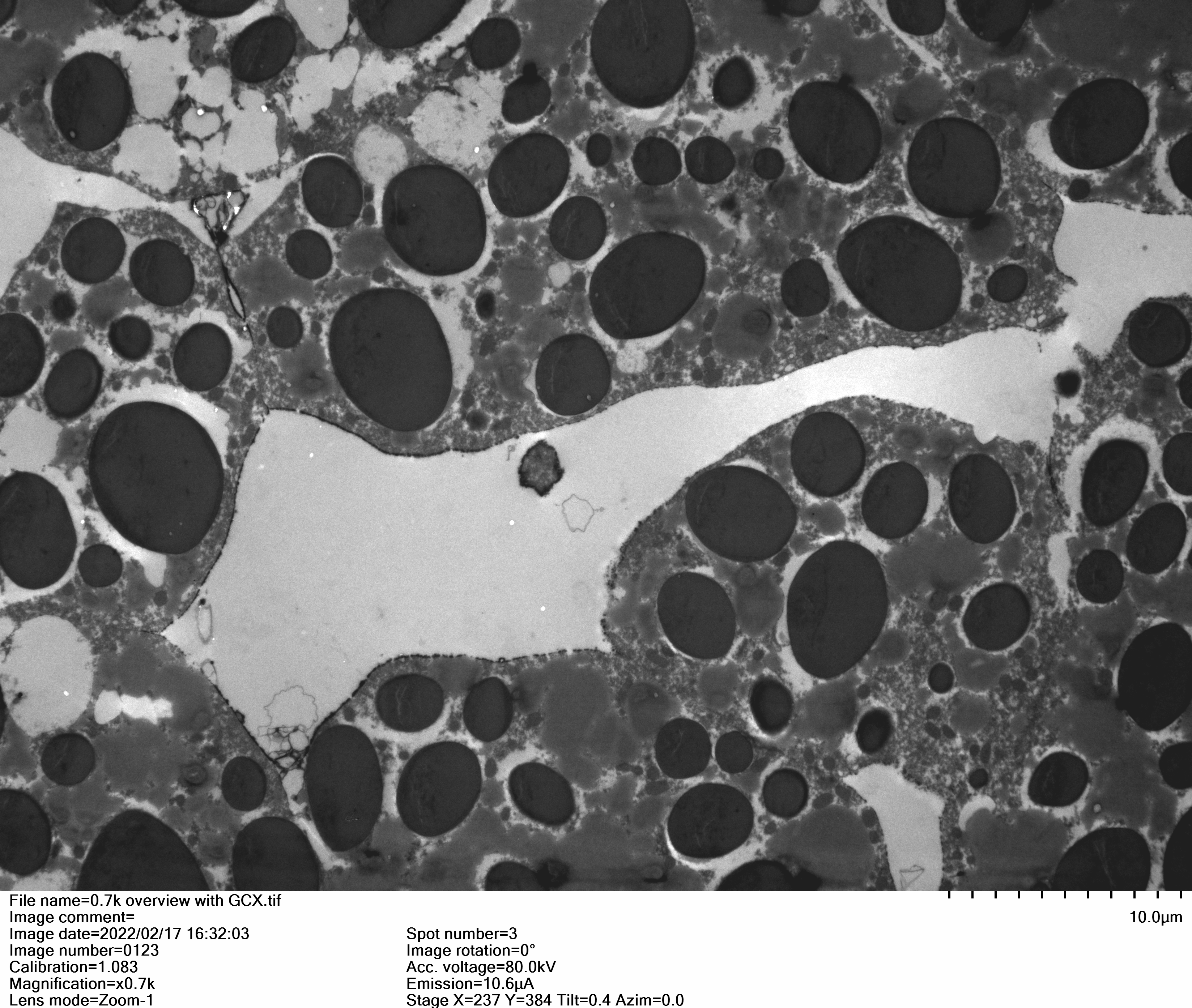

Supplement: S1 Dataset — (ZIP) [file pone.0297420.s008.zip › 0.7k CadMO La.tif]

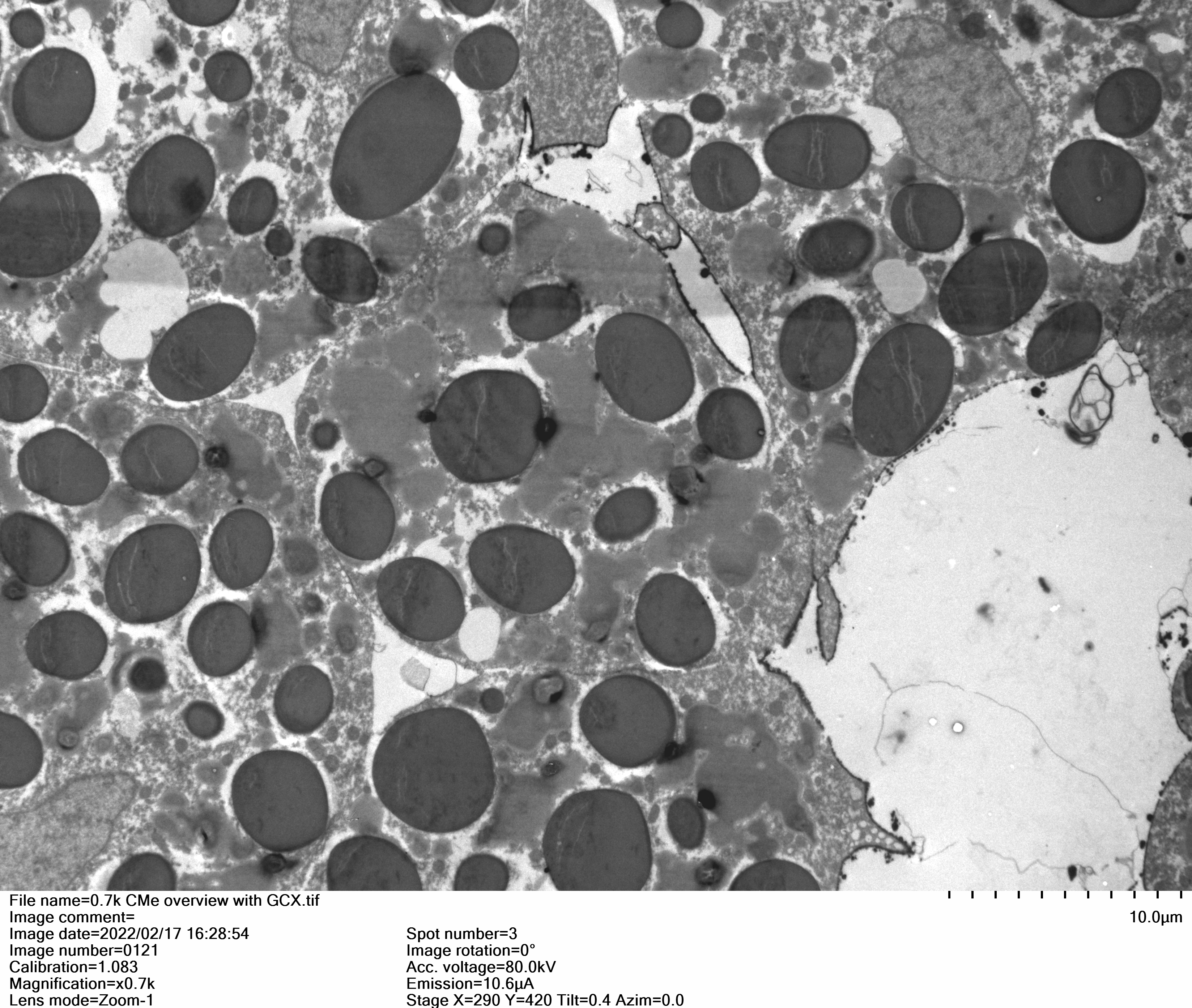

Supplement: S1 Dataset — (ZIP) [file pone.0297420.s008.zip › 0.7k CMe CadMO La.tif]

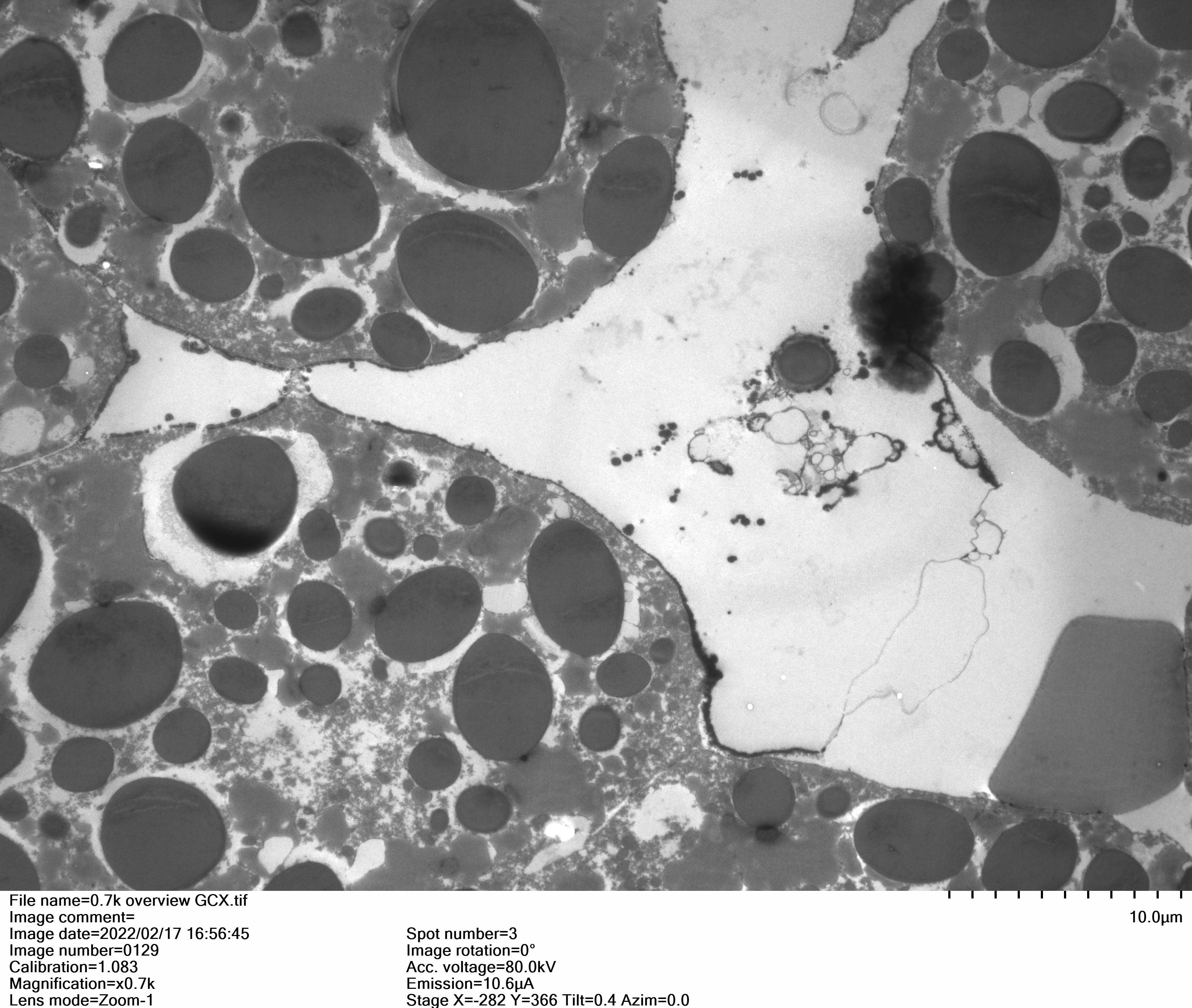

Supplement: S1 Dataset — (ZIP) [file pone.0297420.s008.zip › 0.7k FNMO La.tif]

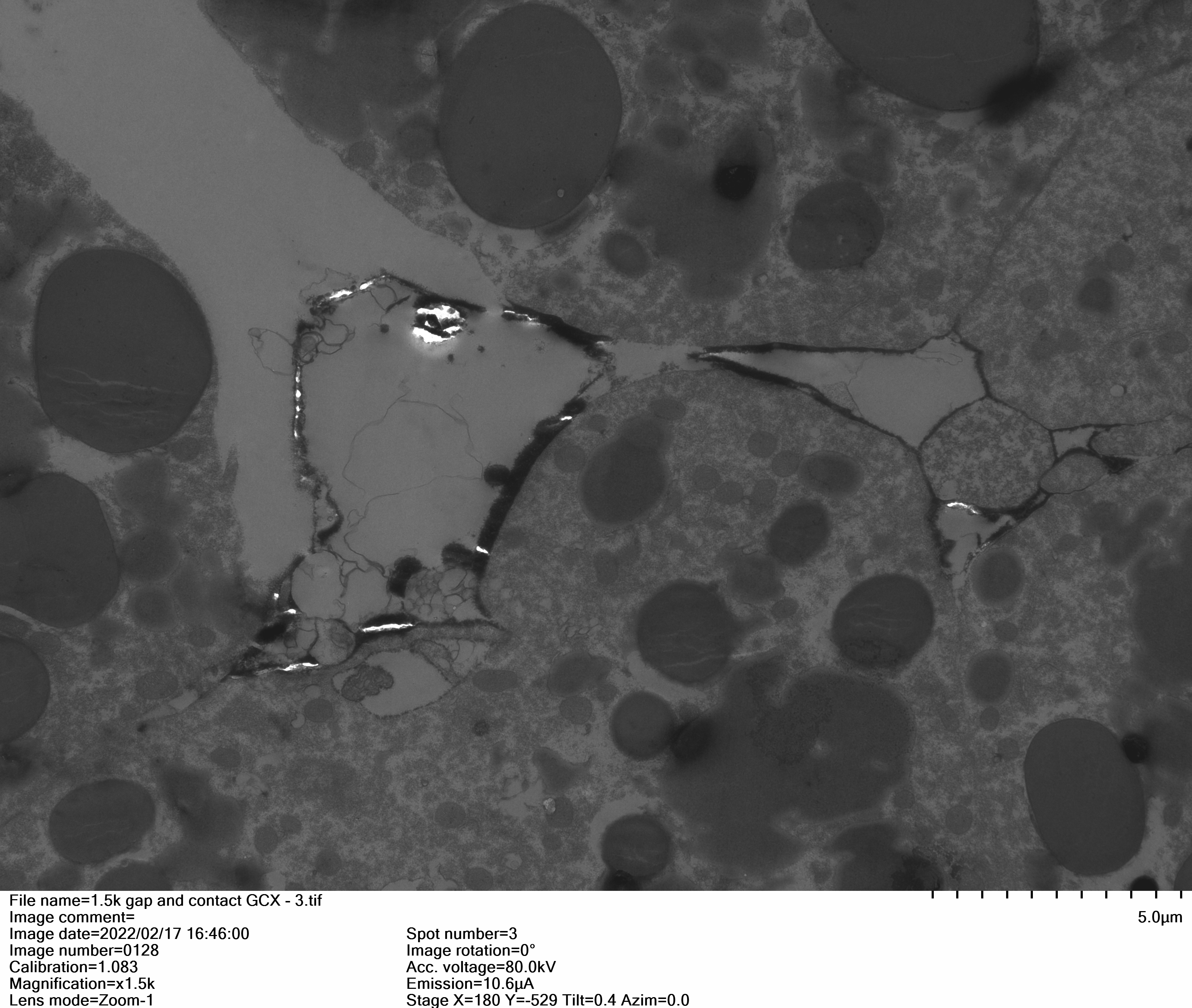

Supplement: S1 Dataset — (ZIP) [file pone.0297420.s008.zip › 1.5k CadMO La2.tif]

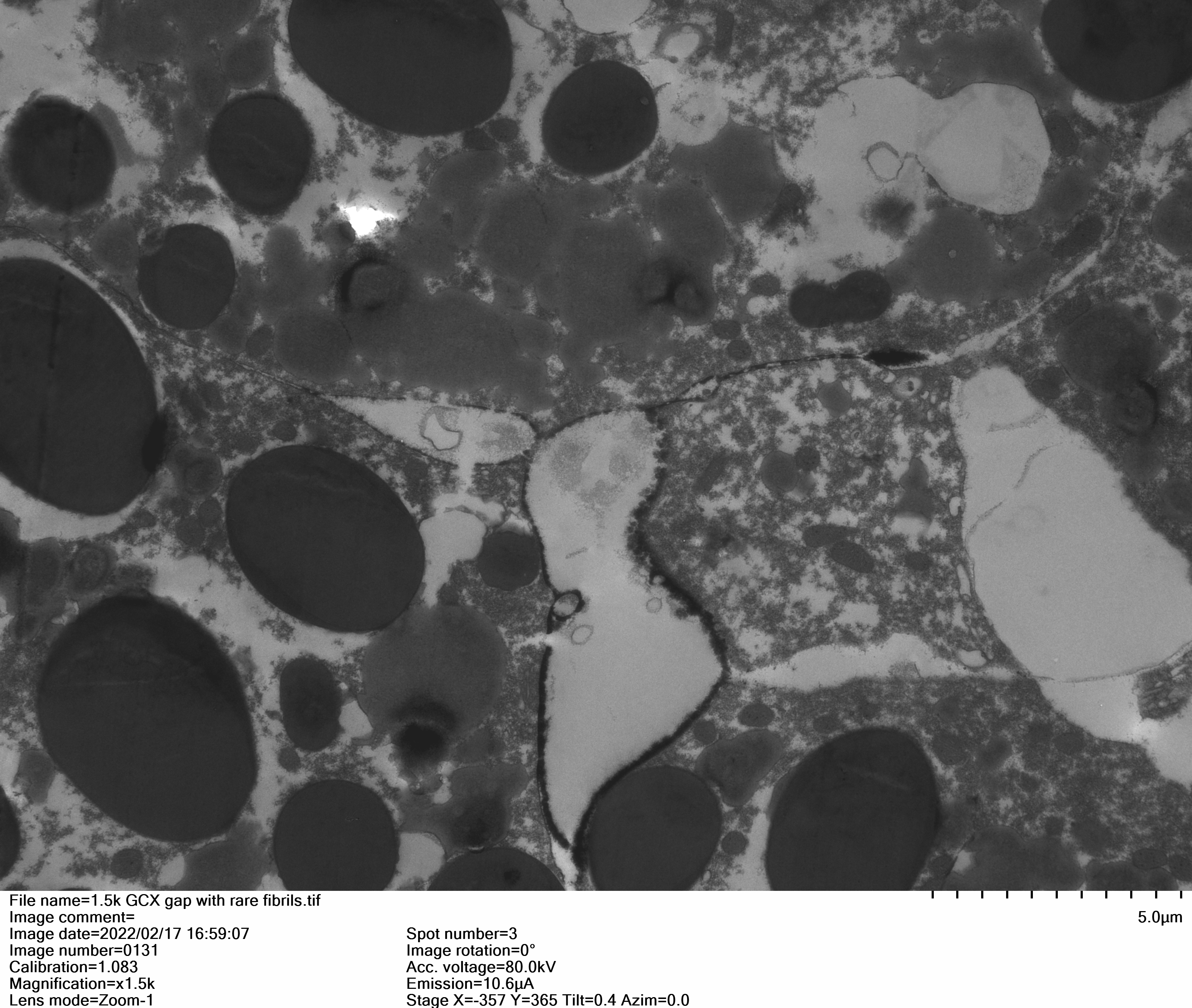

Supplement: S1 Dataset — (ZIP) [file pone.0297420.s008.zip › 1.5k FNMO La2.tif]

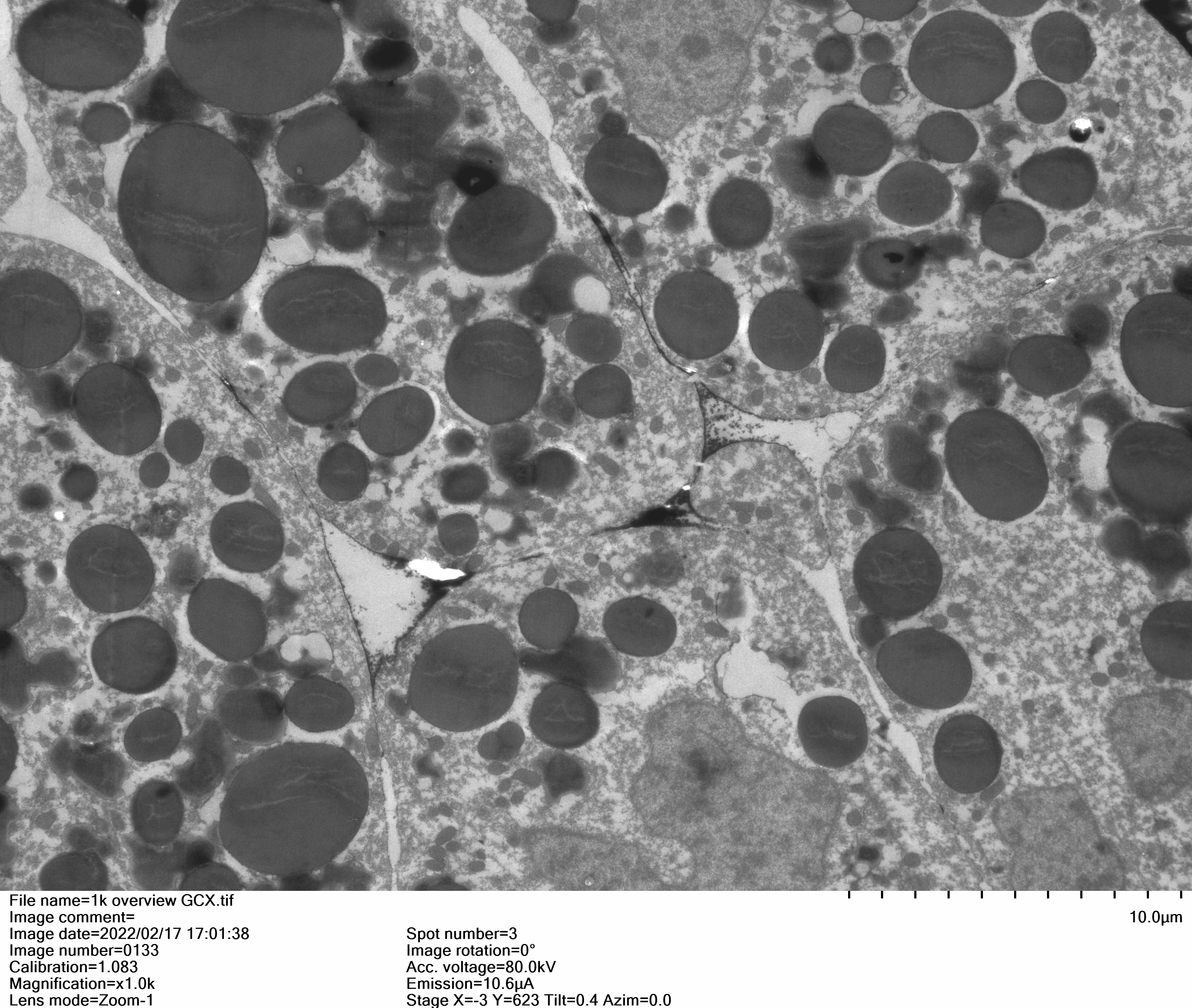

Supplement: S1 Dataset — (ZIP) [file pone.0297420.s008.zip › 1k FNMO La3.tif]

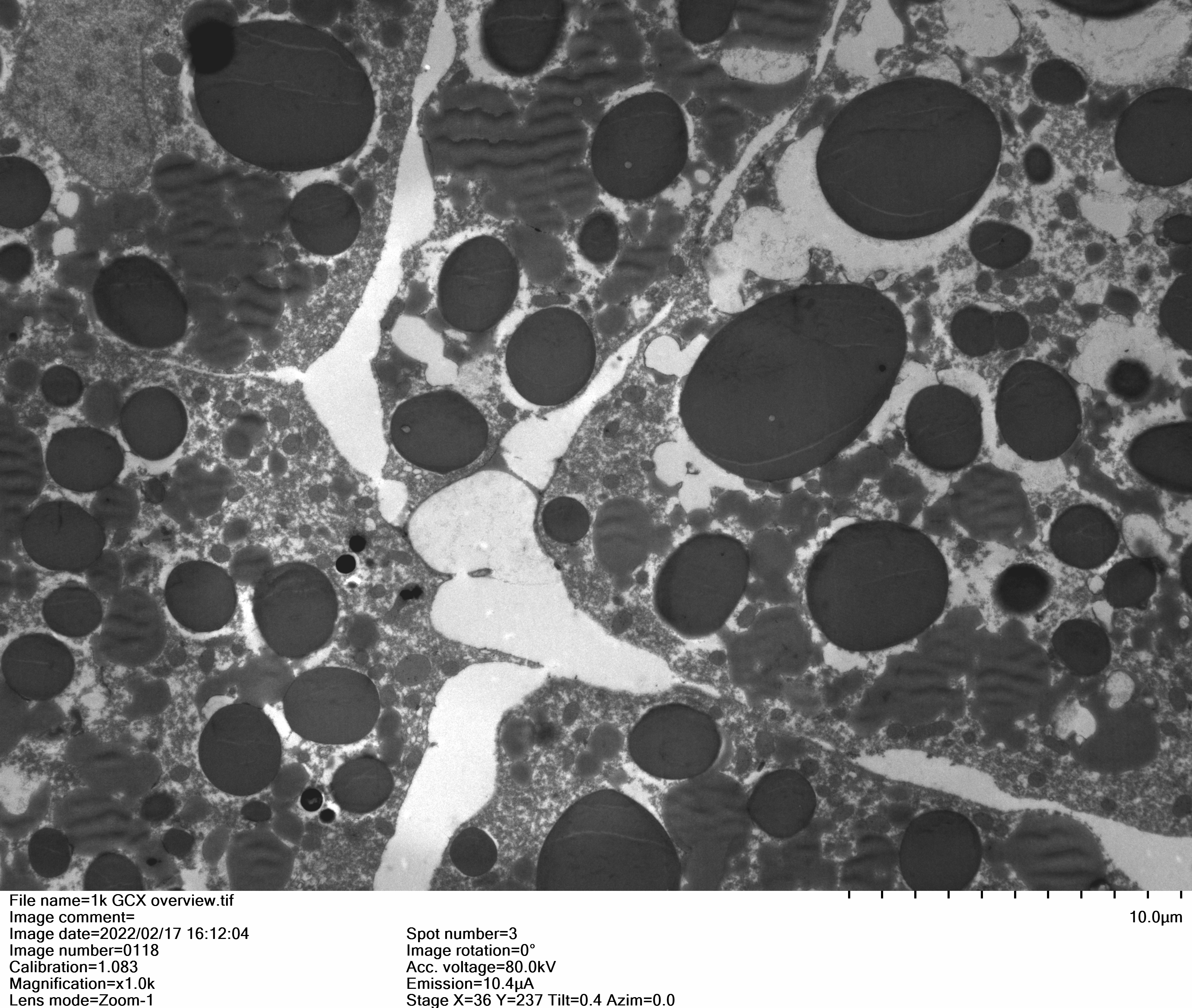

Supplement: S1 Dataset — (ZIP) [file pone.0297420.s008.zip › 1k Syn4MO La1.tif]

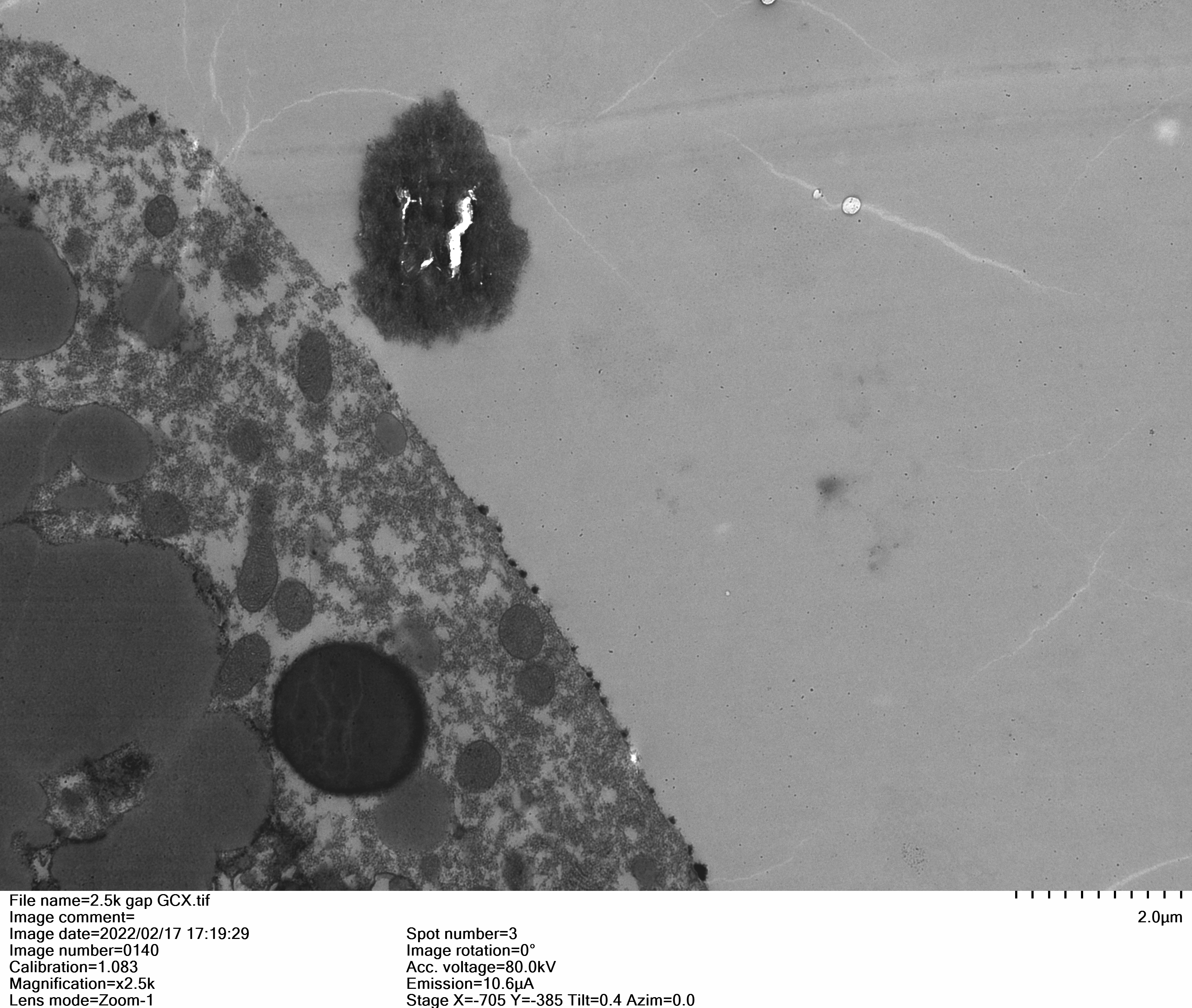

Supplement: S1 Dataset — (ZIP) [file pone.0297420.s008.zip › 2.5k Syn4MO La2.tif]

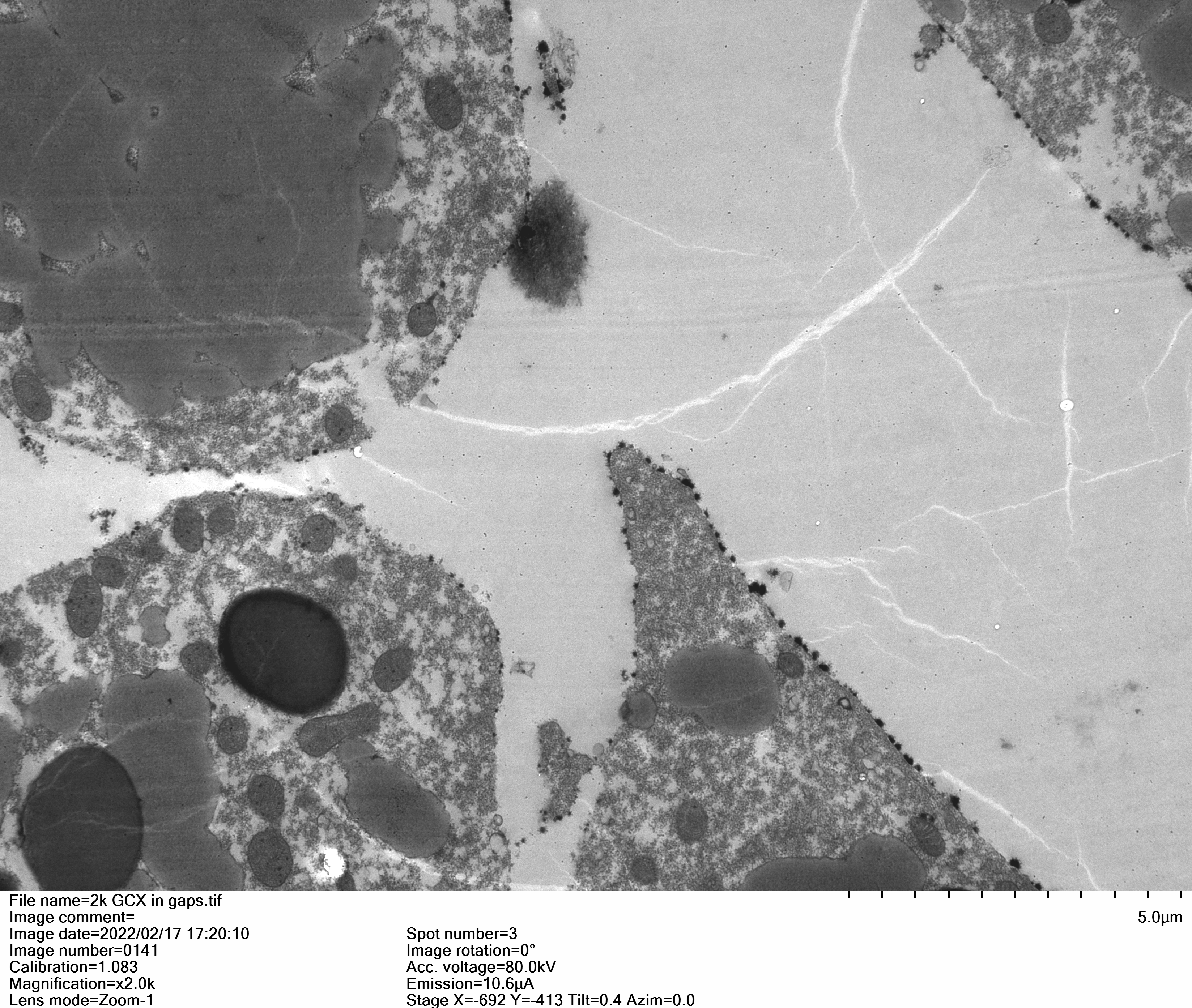

Supplement: S1 Dataset — (ZIP) [file pone.0297420.s008.zip › 2k Syn4MO La3.tif]

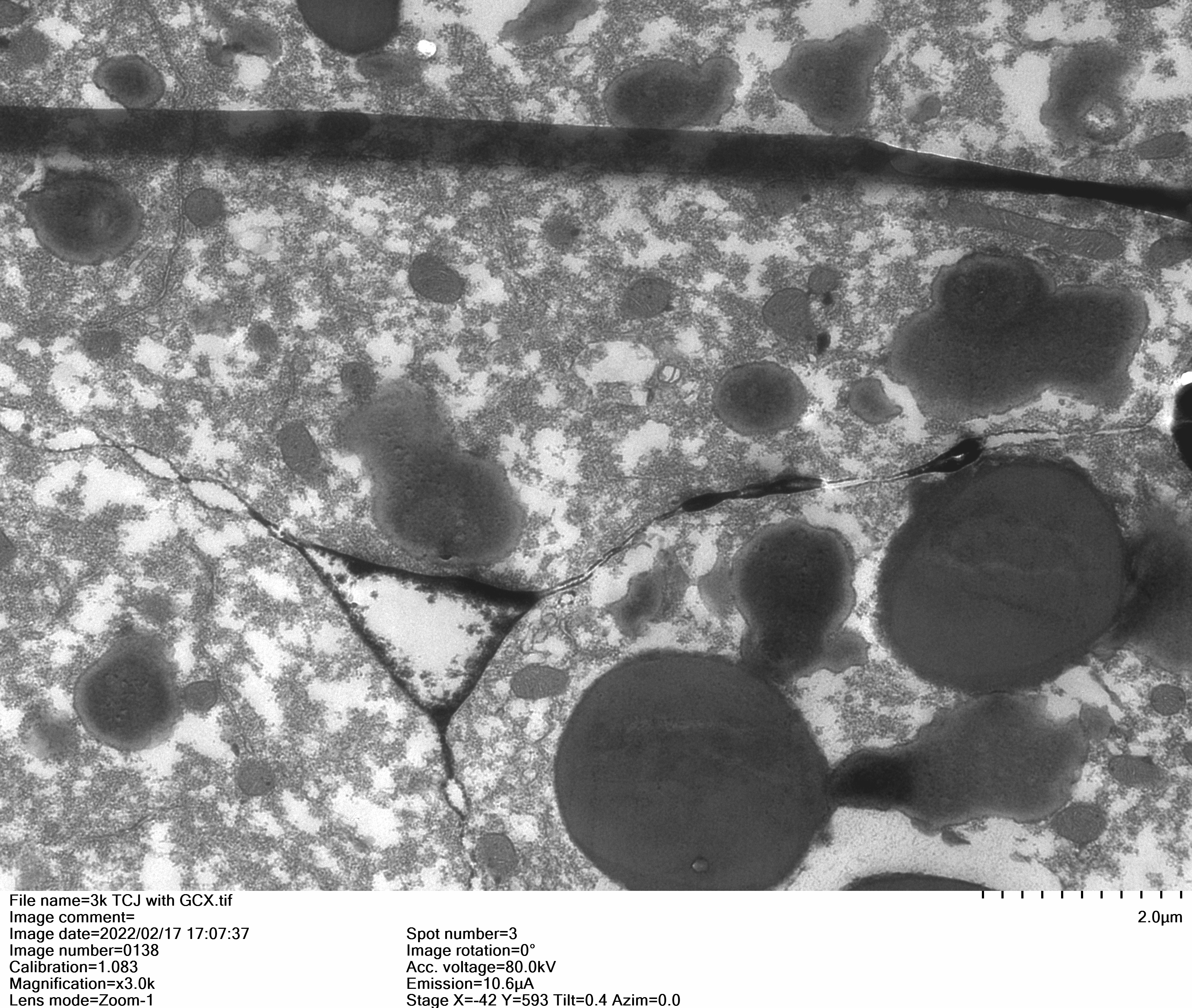

Supplement: S1 Dataset — (ZIP) [file pone.0297420.s008.zip › 3k FNMO La4.tif]

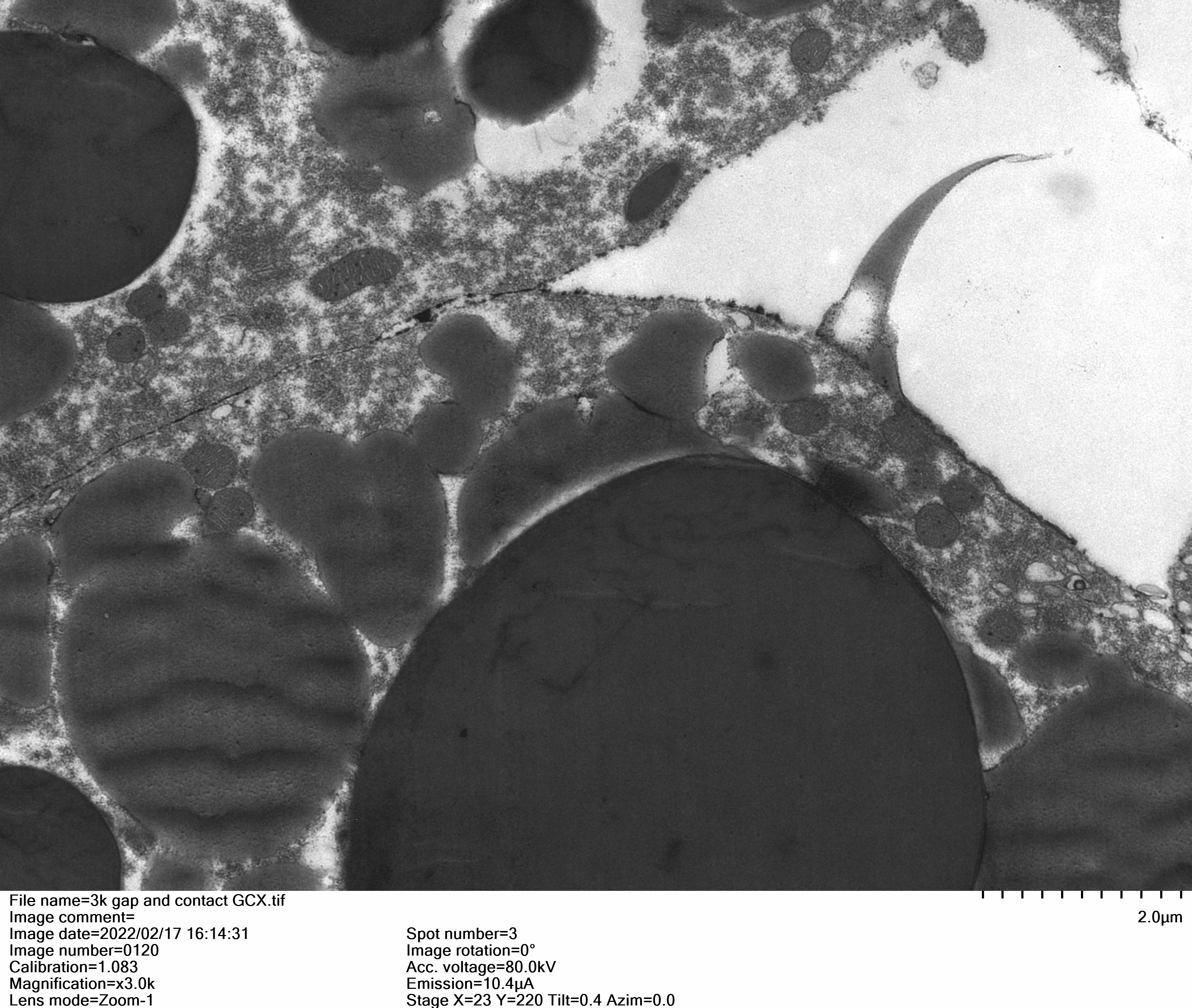

Supplement: S1 Dataset — (ZIP) [file pone.0297420.s008.zip › 3k Syn4MO La4.tif]

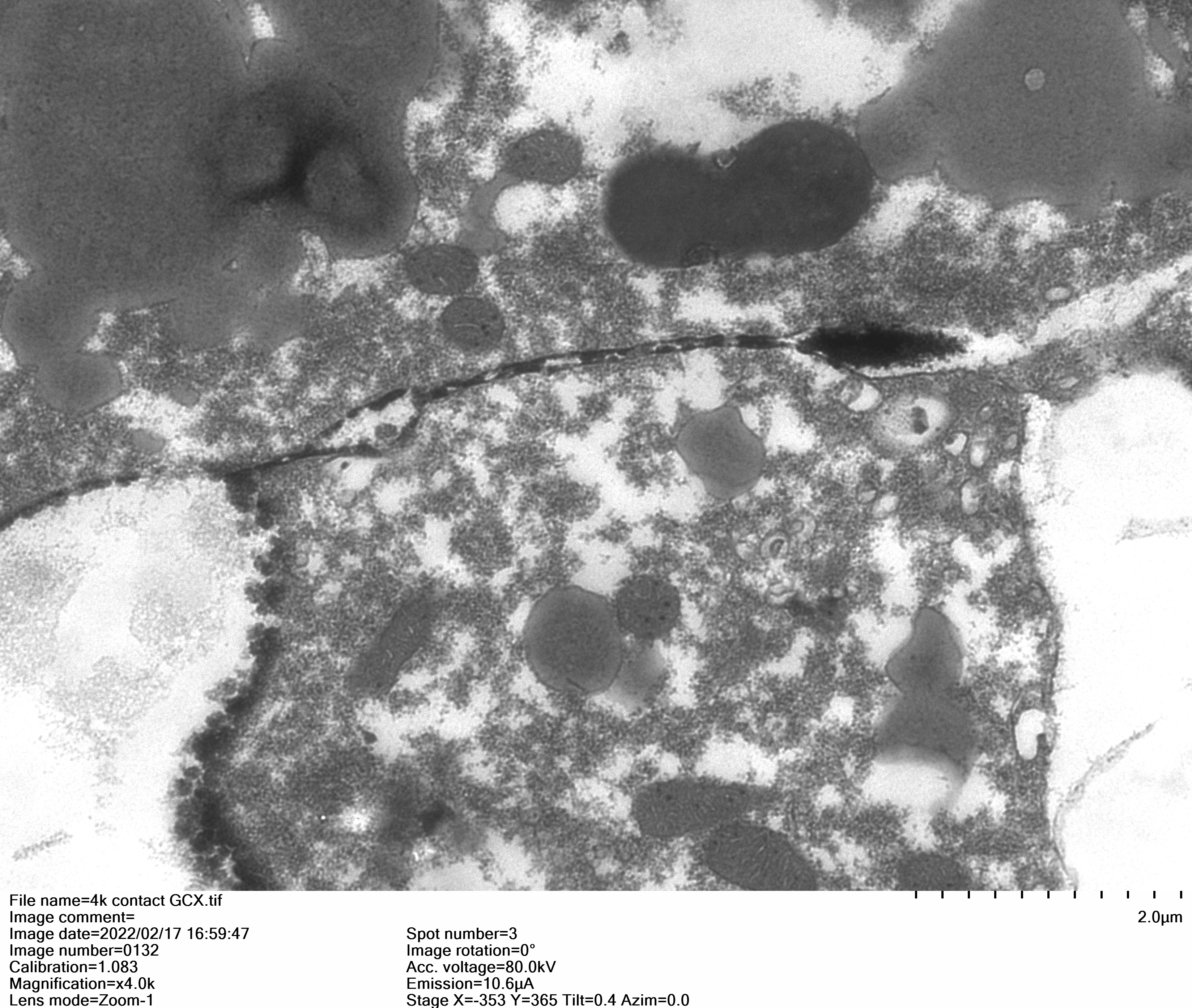

Supplement: S1 Dataset — (ZIP) [file pone.0297420.s008.zip › 4k FNMO La5.tif]

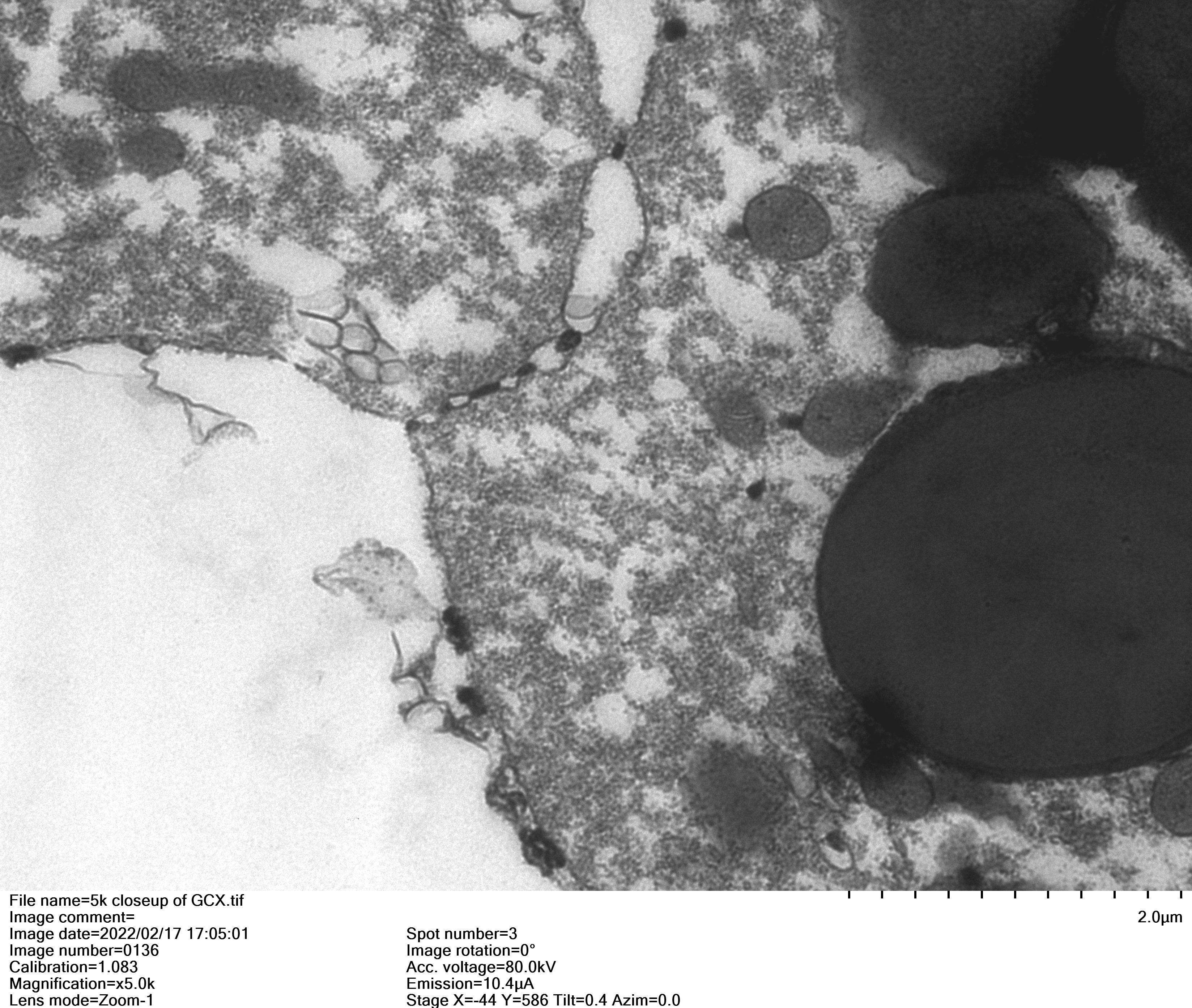

Supplement: S1 Dataset — (ZIP) [file pone.0297420.s008.zip › 5k Syn4MO La5.tif]

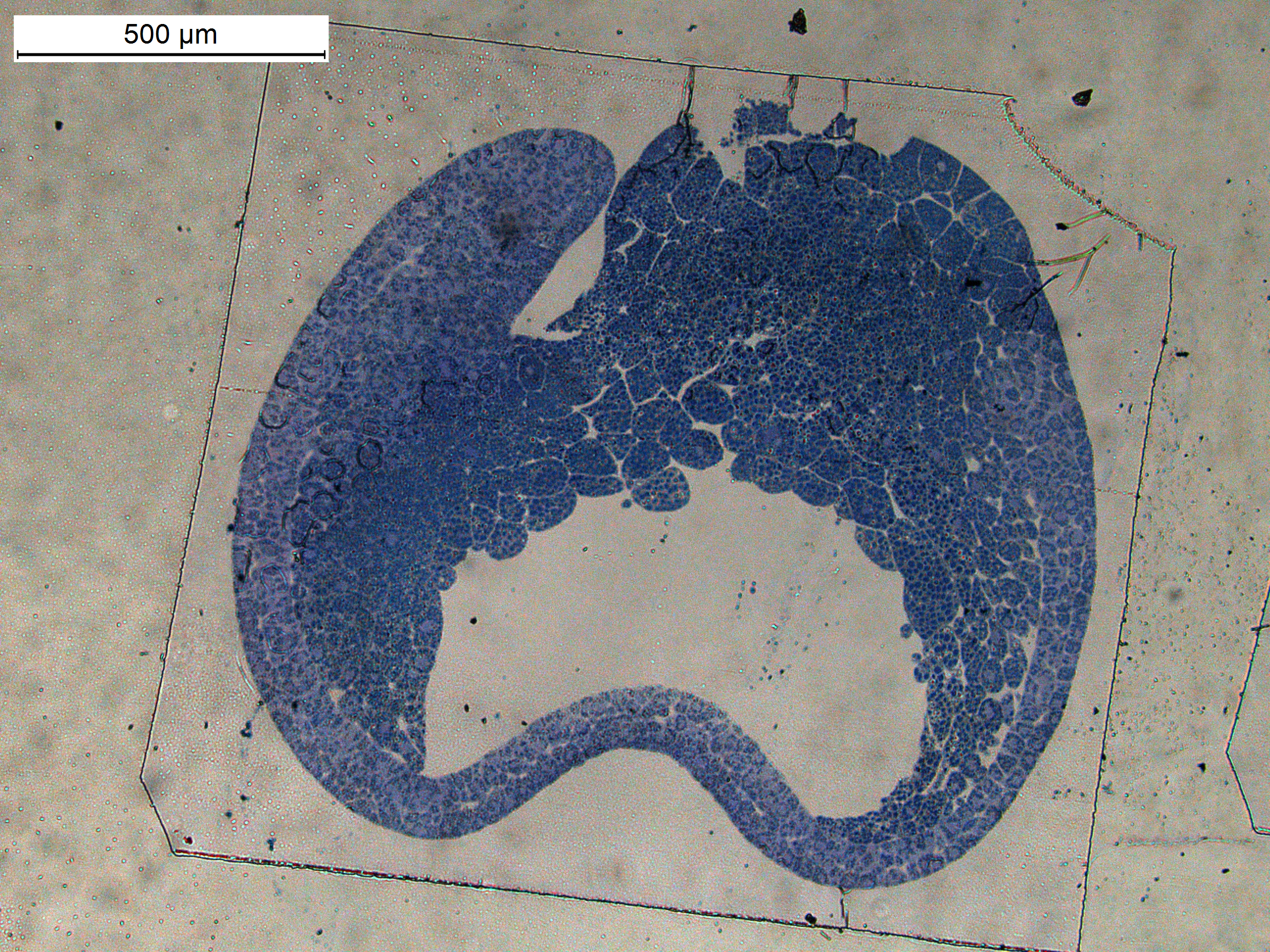

Supplement: S1 Dataset — (ZIP) [file pone.0297420.s008.zip › 8WT1A.tif]

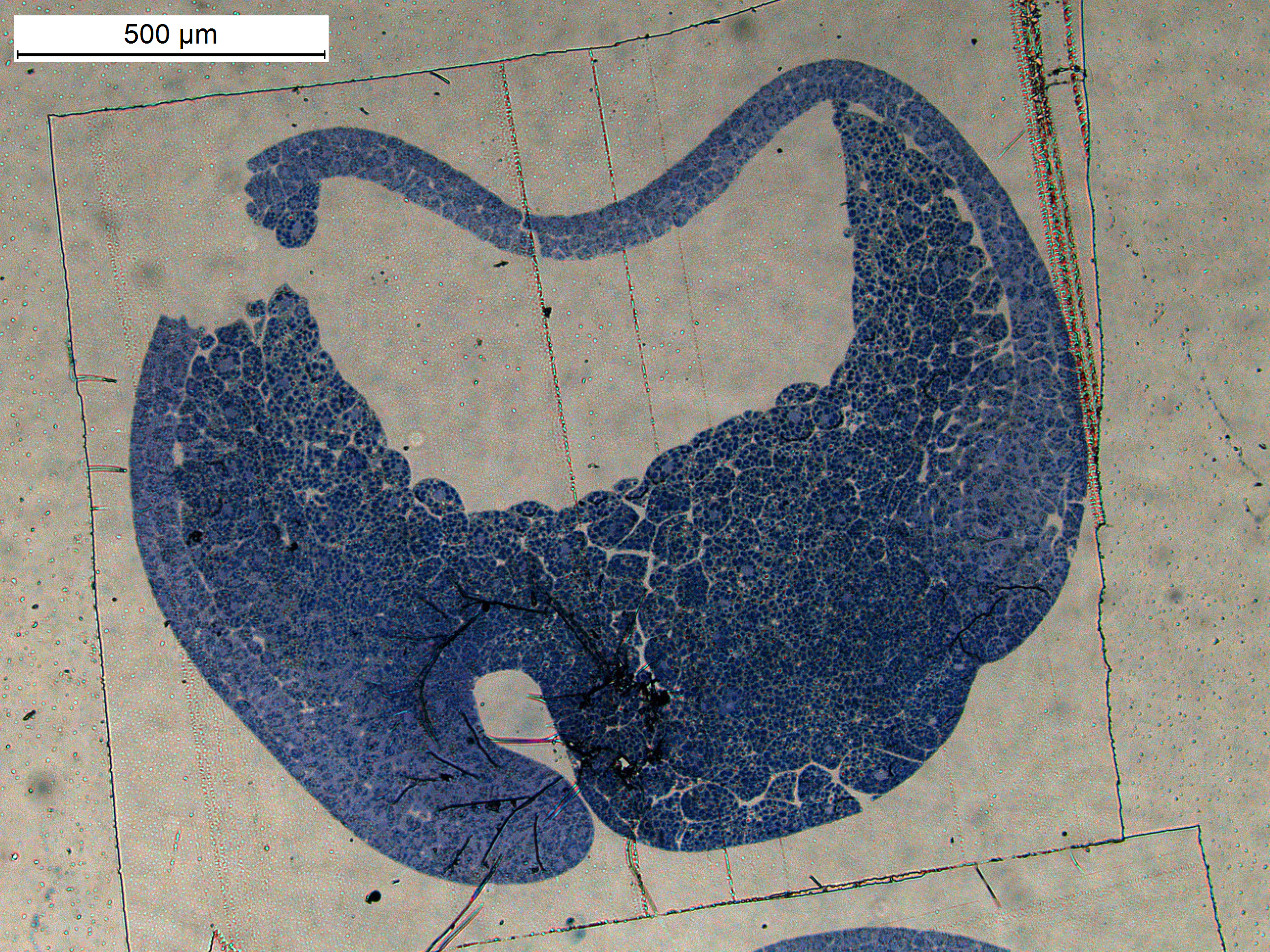

Supplement: S1 Dataset — (ZIP) [file pone.0297420.s008.zip › 8WT6A.tif]

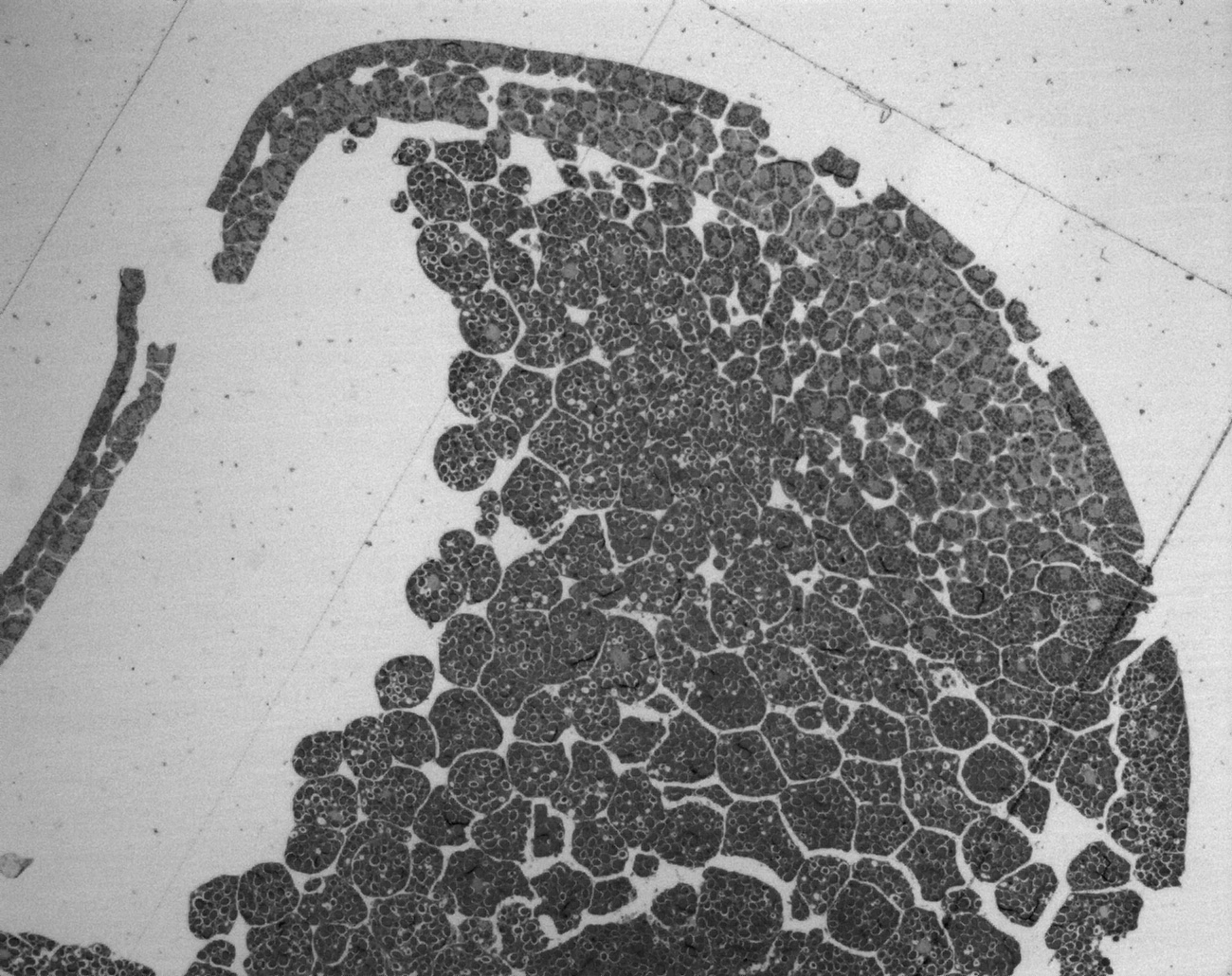

Supplement: S1 Dataset — (ZIP) [file pone.0297420.s008.zip › CadMO thick1.jpg]

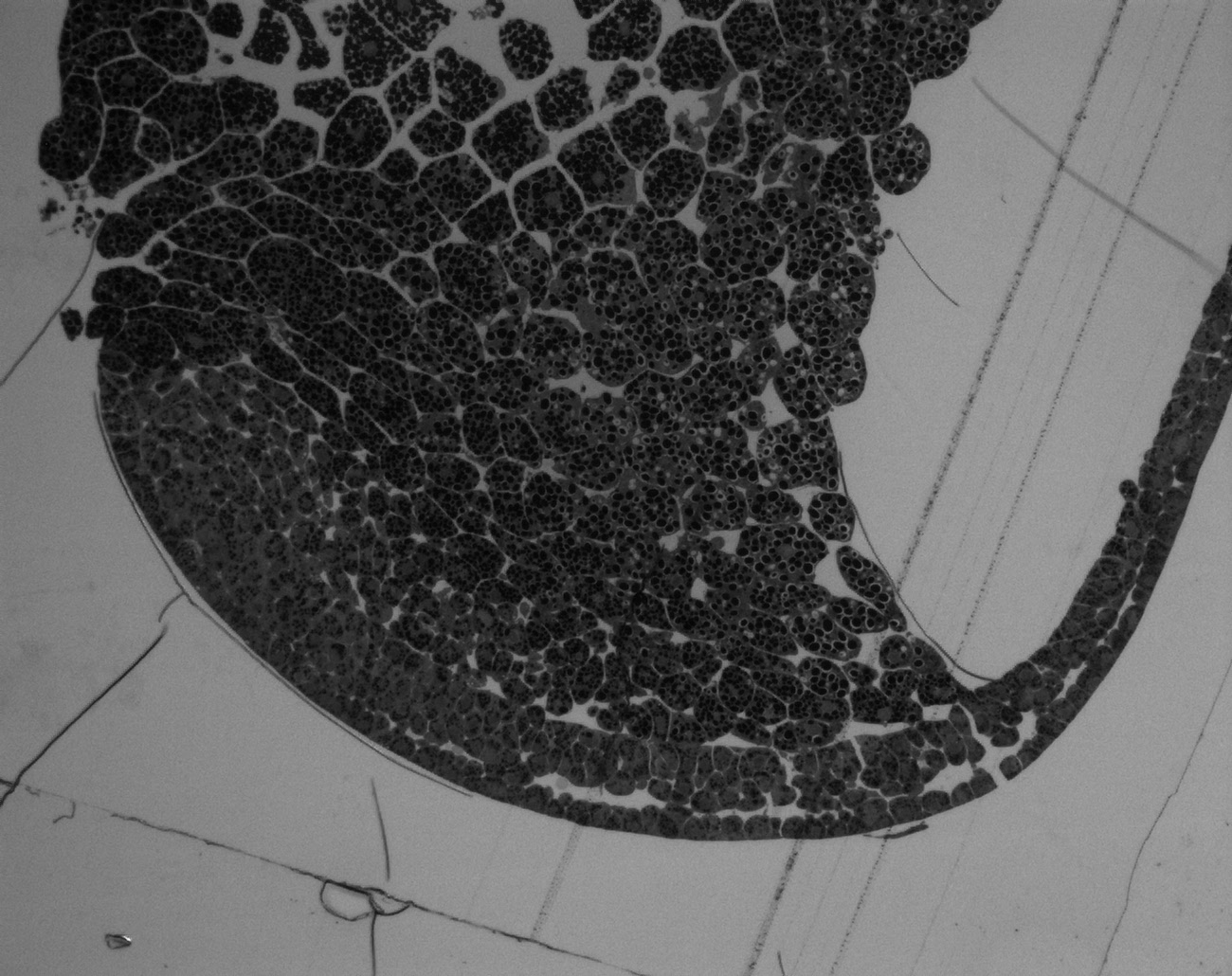

Supplement: S1 Dataset — (ZIP) [file pone.0297420.s008.zip › CadMO thick2.jpg]

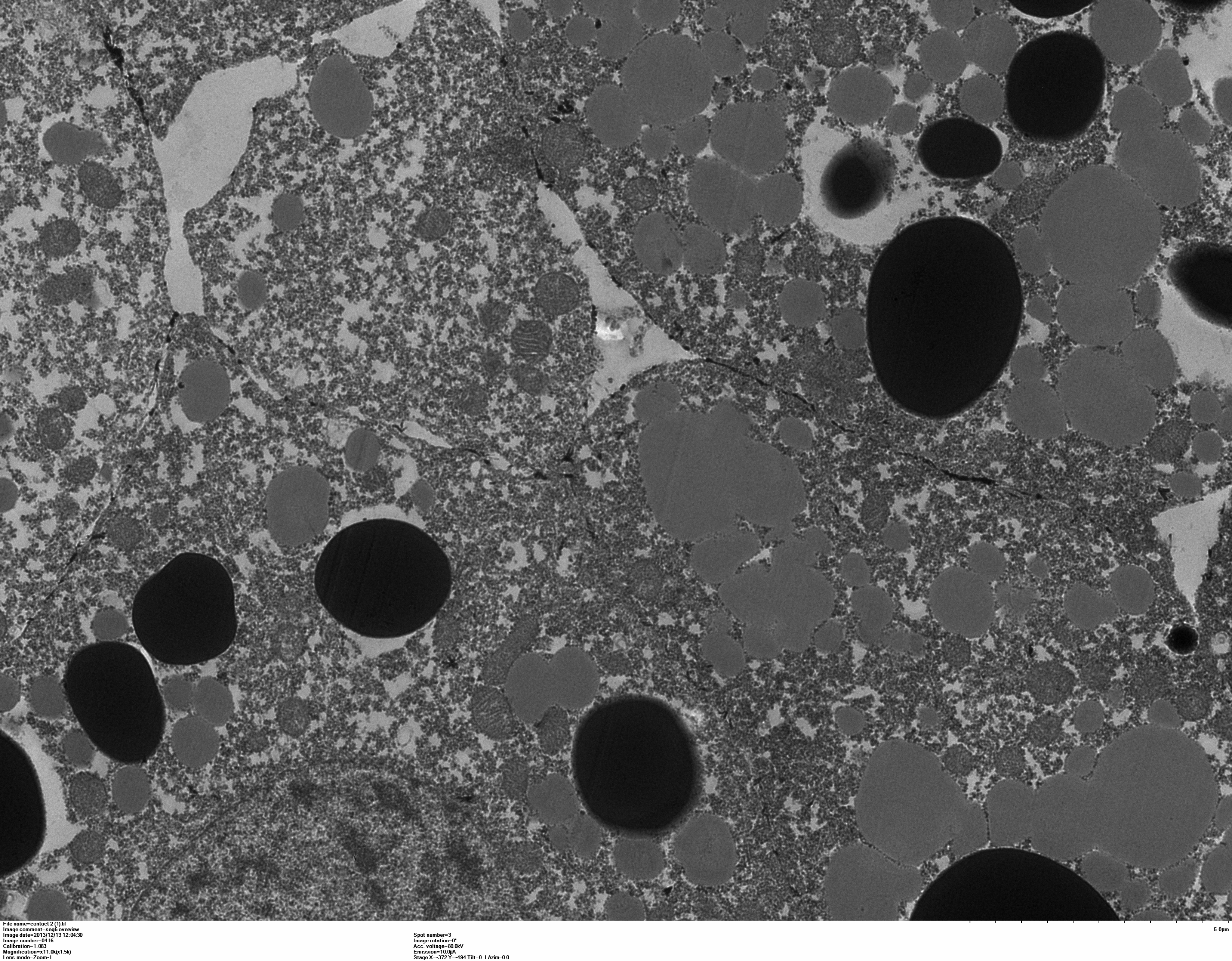

Supplement: S1 Dataset — (ZIP) [file pone.0297420.s008.zip › ecto La1.tif]

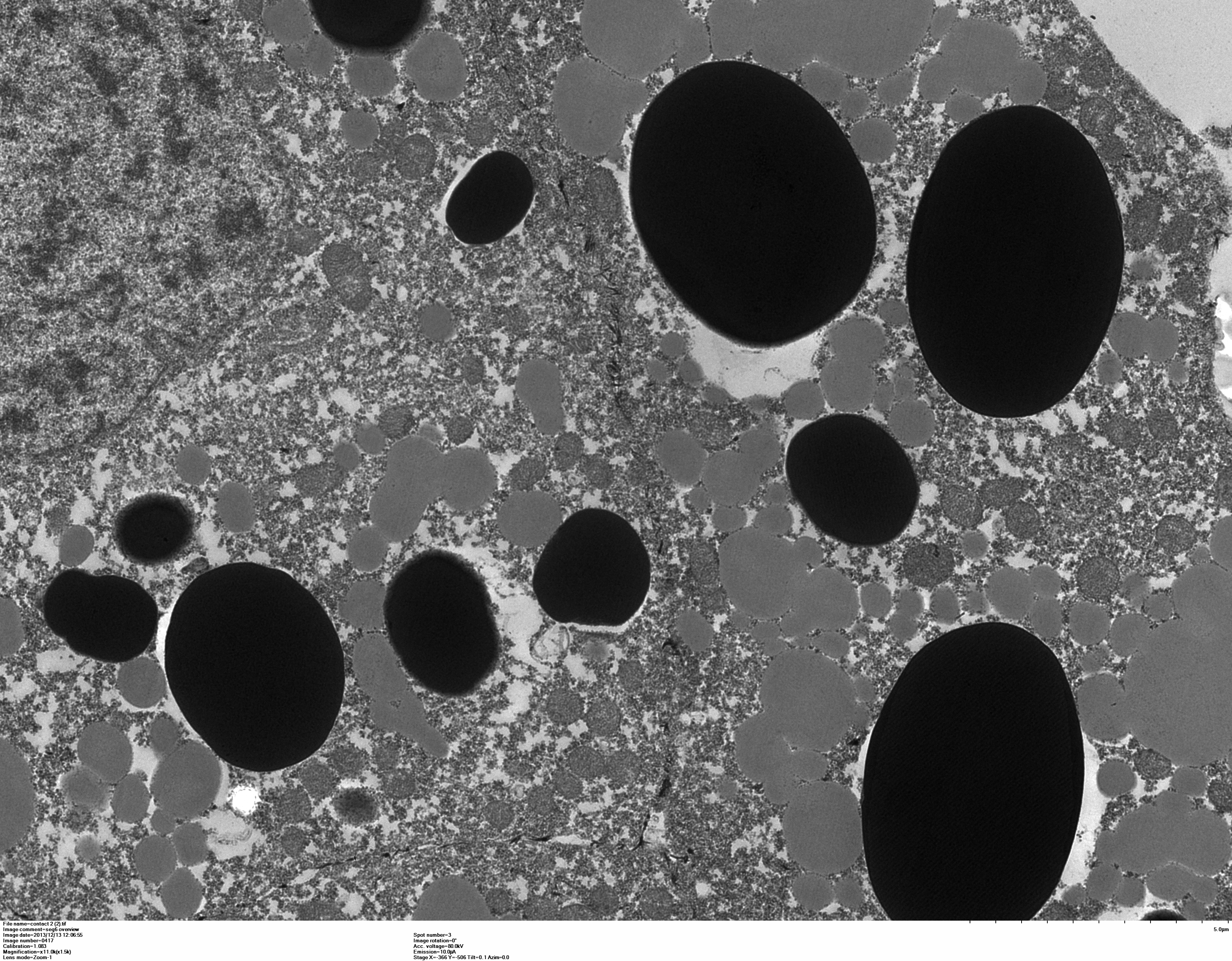

Supplement: S1 Dataset — (ZIP) [file pone.0297420.s008.zip › ecto La2.tif]

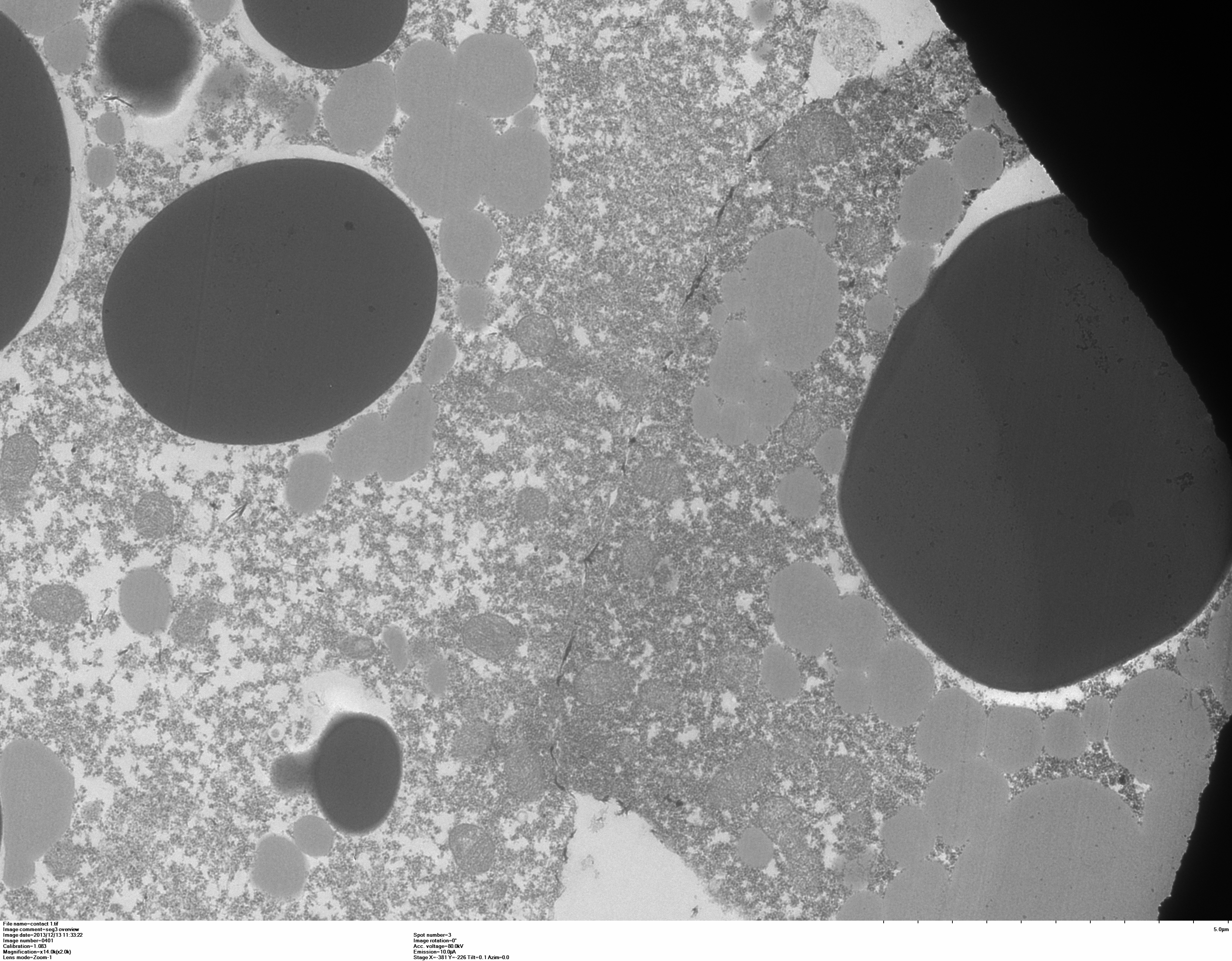

Supplement: S1 Dataset — (ZIP) [file pone.0297420.s008.zip › ecto La3.tif]

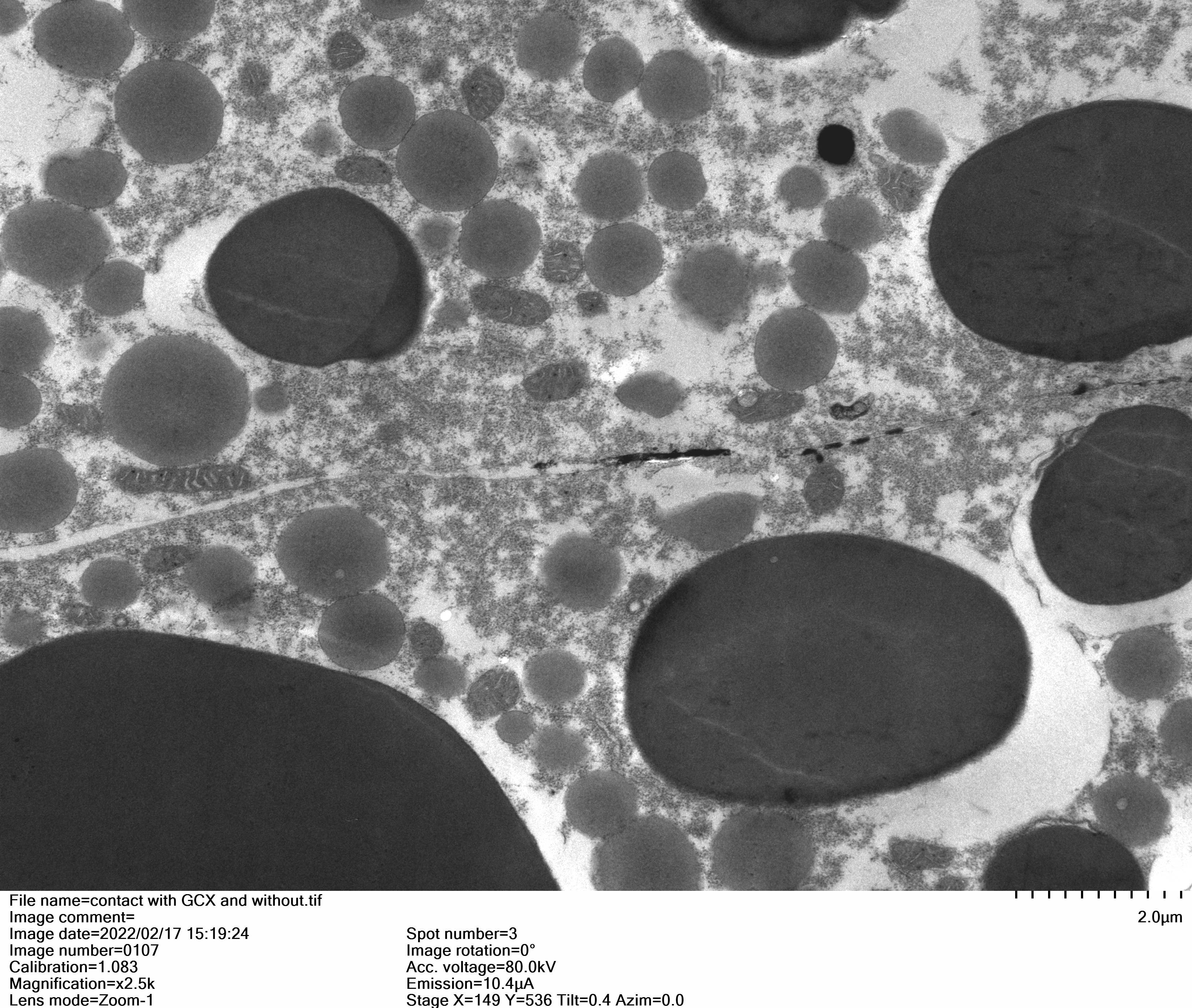

Supplement: S1 Dataset — (ZIP) [file pone.0297420.s008.zip › Has1MO La2.tif]

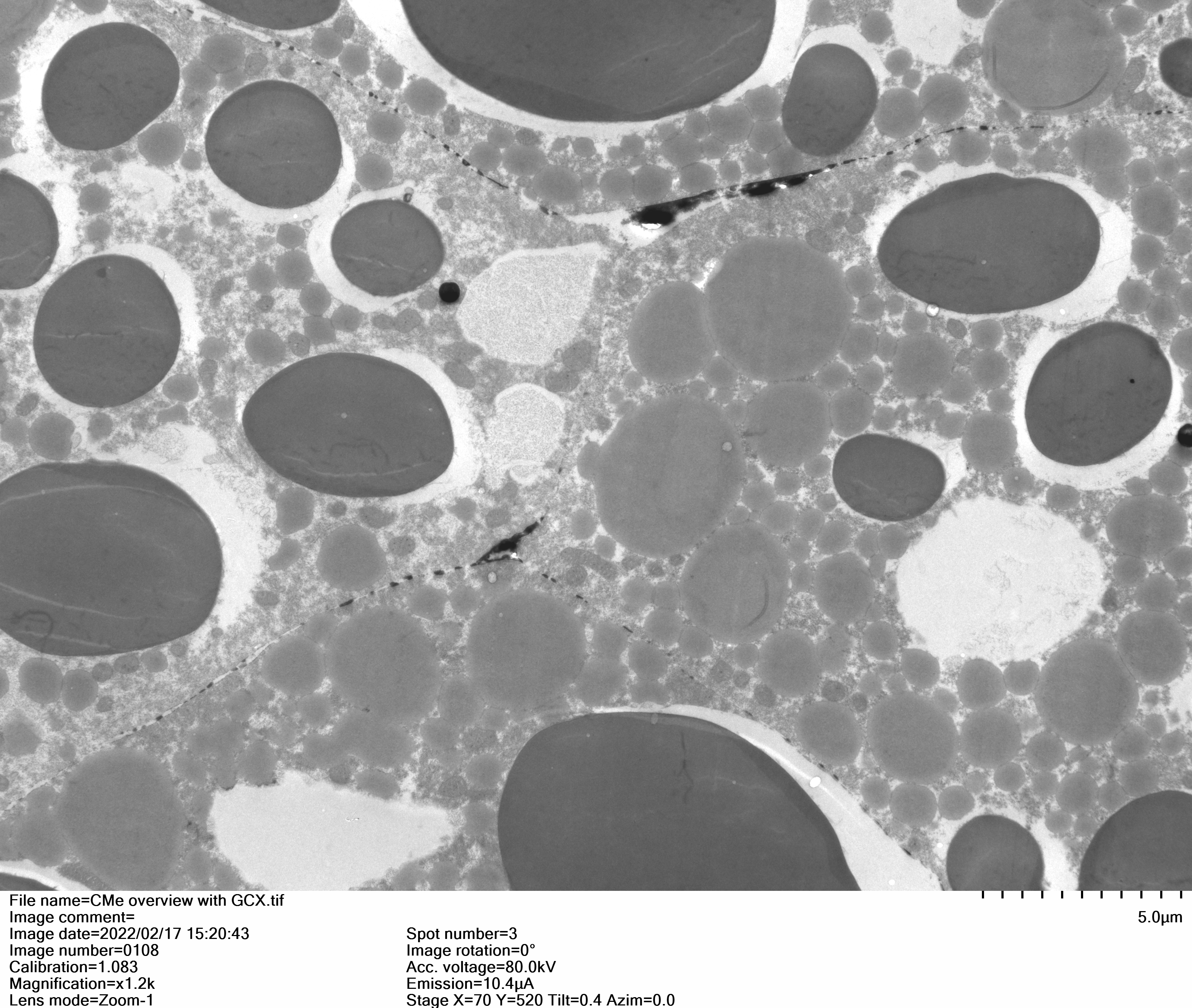

Supplement: S1 Dataset — (ZIP) [file pone.0297420.s008.zip › Has1MO La3.tif]

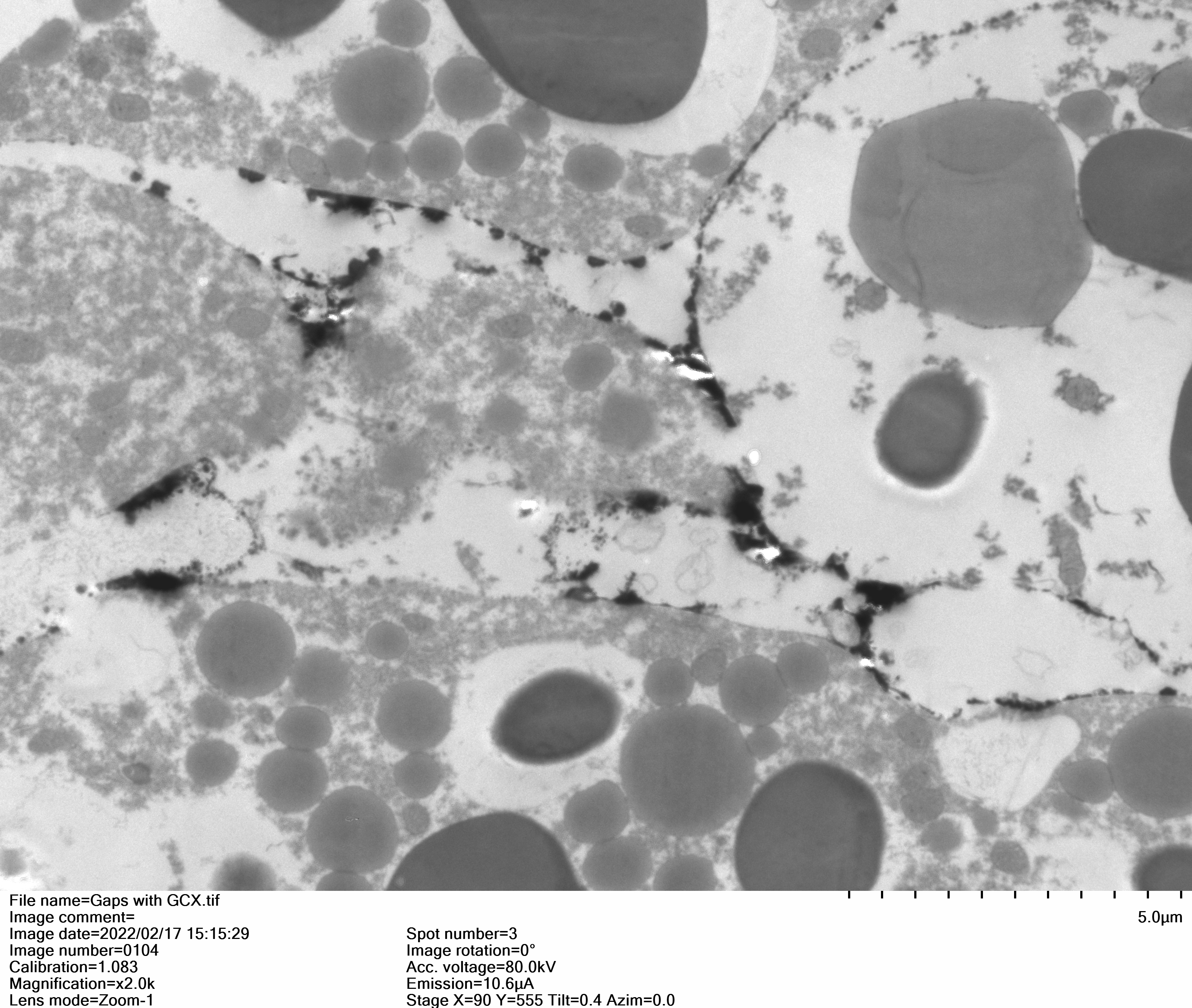

Supplement: S1 Dataset — (ZIP) [file pone.0297420.s008.zip › Has1MO La4.tif]

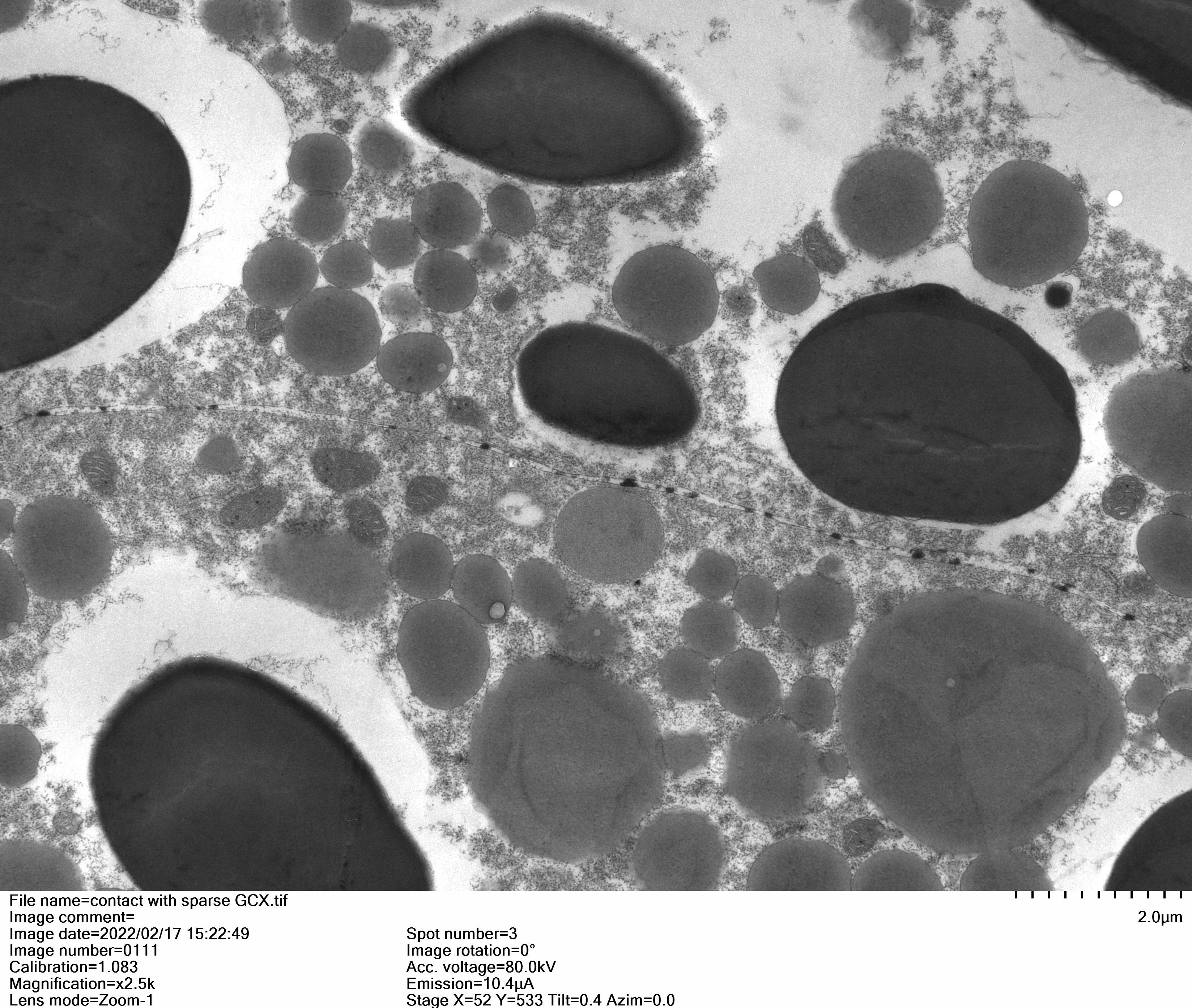

Supplement: S1 Dataset — (ZIP) [file pone.0297420.s008.zip › Has1MO La5.tif]
