# Supplementary material for: Cell contacts and pericellular matrix in the Xenopus gastrula chordamesoderm
Source: PLoS One. 2024 Feb 12;19(2):e0297420. doi: 10.1371/journal.pone.0297420 (PMC10861091; doi:10.1371/journal.pone.0297420)
Supplement: S1 Table — Av, average of α values; SD, standard deviation; n, number of α-w data points, i.e. gap-contact transitions; p (MO/wt), p-values for significance morphants vs. untreated CM (wt); p (low/high), p-values for significance low vs. high contact widths. (PDF) [file pone.0297420.s005.pdf]

S1 Table

Relative adhesiveness α at low (w < 250 nm) and high (w ≥ 250 nm) contact widths.

	Contact width < 250 nm					Contact width ≥ 250 nm			
	Av	SD	n	p(MO/wt)		Av	SD	n	p (low/high)
Wt	0.1326	0.0898	143			0.2260	0.1380	6	0.0158
CadMO	0.0966	0.0786	259	0.0001		0.2495	0.1380	249	0.0001
FNMO	0.1030	0.0871	476	0.0004		0.1853	0.1244	53	0.0001
Has2MO	0.0842	0.0693	165	0.0001		0.2040	0.1234	91	0.0001
Syn4MO	0.2339	0.1611	423	0.0001		0.3806	0.1834	408	0.0001

Av, average of α values; SD, standard deviation; n, number of α–w data points, i.e. gap-contact transitions; p (MO/wt), p-values for significance morphants vs. untreated CM (wt); p (low/high), p-values for significance low vs. high contact widths.
