# Supplementary material for: Cell contacts and pericellular matrix in the Xenopus gastrula chordamesoderm
Source: PLoS One. 2024 Feb 12;19(2):e0297420. doi: 10.1371/journal.pone.0297420 (PMC10861091; doi:10.1371/journal.pone.0297420)
Supplement: S2 Table — Regression lines delineating the lower boundaries of α-w distributions were determined as described in the main text. α0, intersection of regression line with α axis; Δα/Δw, slope of regression line; r, regression coefficient; d, interdigitation distance d = 2α0/(Δα/Δw). The calculated d is comparable to the measured average lengths of the shortest LSM units (“stubs”) in normal contacts (156 nm), FN morphants (128 nm) and Has1 morphants (111 nm) shown in Fig 6F–6H. (PDF) [file pone.0297420.s006.pdf]

S2 Table

Positions of first peak in α frequency distributions in consecutive width brackets.

Center of width bracket	25 nm	75 nm	150 nm	300 nm	600 nm	1000 nm
Wt	0.088	0.063	0.088	0.133		
CadMO	0.038	0.038	0.088	0.133	0.138	0.163
FNMO	0.063	0.063	0.063	0.063	0.163	
Has2MO	0.038	0.063	0.038	0.063	0.163	
Syn4MO	0.088	0.063	0.088	0.113	0.138	0.213
Average morphants	0.057	0.057	0.069	0.088	0.151	

Frequencies of α were binned in 0.025 intervals of α and the position of the first peak is indicated as the mid-point of a bin, as read off from Fig 8 and S3 Fig, for width brackets 0-50 nm, 50-100 nm, 100-200 nm, 200-400 nm, 400-800 nm, and 800-1200 nm. The mid-points of these brackets are indicated.
