# Supplementary material for: Cell contacts and pericellular matrix in the Xenopus gastrula chordamesoderm
Source: PLoS One. 2024 Feb 12;19(2):e0297420. doi: 10.1371/journal.pone.0297420 (PMC10861091; doi:10.1371/journal.pone.0297420)
Supplement: S3 Table — Frequencies of α were binned in 0.025 intervals of α and the position of the first peak is indicated as the mid-point of a bin, as read off from Fig 8 and S3 Fig, for width brackets 0–50 nm, 50–100 nm, 100–200 nm, 200–400 nm, 400–800 nm, and 800–1200 nm. The mid-points of these brackets are indicated. (PDF) [file pone.0297420.s007.pdf]

S3 Table

Regression line parameters.

	α_0	$\Delta\alpha/\Delta w$ [μm^{-1}]	r	d [nm]
wt	0.017	0.261	0.935	130
CadMO	0.013	0.114	0.988	227
FNMO	0.014	0.119	0.978	235
HasMO	0.009	0.102	0.972	172
Syn4MO	0.023	0.074	0.960	625

Regression lines delineating the lower boundaries of α – w distributions were determined as described in the main text. α_0, intersection of regression line with α axis; $\Delta\alpha/\Delta w$, slope of regression line; r, regression coefficient; d, interdigitation distance $d = 2\alpha_0/(\Delta\alpha/\Delta w)$. The calculated d is comparable to the measured average lengths of the shortest LSM units ("stubs") in normal contacts (156 nm), FN morphants (128 nm) and Has1 morphants (111 nm) shown in Fig 6F-H.
